# Category learning drives cortical attentional gain to diagnostic dimensions of perceptual information

**DOI:** 10.1371/journal.pbio.3004076

**Published:** 2026-11-12

**Authors:** Sahil Luthra, Raha N. Razin, Adam T. Tierney, Lori L. Holt, Frederic Dick

**Affiliations:** 1 Department of Psychology, Stony Brook University, Stony Brook, New York, United States of America; 2 Department of Experimental Psychology, Division of Psychological and Language Sciences, University College London, London, United Kingdom; 3 Department of Psychological Sciences, Birkbeck, University of London, London, United Kingdom; 4 Department of Psychology, The University of Texas at Austin, Austin, Texas, United States of America; Universidad de Salamanca, SPAIN

## Abstract

Humans and other animals develop remarkable perceptual and cognitive specializations for identifying, differentiating, and acting on classes of ecologically important signals. This expertise is flexible enough to support diverse perceptual judgments: a voice, for example, simultaneously conveys what a talker says, as well as myriad cues about her identity and state. Expert perception across complex signals thus involves discovering and learning regularities that best inform diverse perceptual judgments, as well as weighting this information flexibly as task demands change. Here, we test whether this process may involve attentional gain. We use two prospective auditory category learning tasks to relate a complex, entirely novel soundscape to four classes of ‘alien identity’ and two classes of ‘alien size.’ Identity, but not size, categorization requires discovery and learning of patterned acoustic input situated in one of two simultaneous, non-overlapping frequency bands. This allows us to capitalize on the coarsely segregated, frequency-band-specific channels tiling auditory cortex. We use fMRI to ask whether category-relevant perceptual information present in one frequency band is prioritized relative to simultaneous, uninformative information in the other frequency band. Only among participants expert at alien identity categorization do we observe neural prioritization of the identity-diagnostic frequency band. This prioritization persists even when the diagnostic information becomes irrelevant in the size categorization task. Tellingly, the neural selectivity evoked implicitly in the identity categorization task aligns with that in an independent task, where activation is driven by explicit and sustained selective attention to pure tones in one or the other frequency band. Additionally, the learning trajectories taken to achieve expert-level categorization leave fingerprints on the patterns of neural activity associated with the diagnostic dimension. In all, this indicates that learning categories can drive attentional gain to category-diagnostic input dimensions.

## Abstract 2

How do humans learn which dimension of perceptual input to prioritize during learning? This human neuroimaging study shows that learning new, complex sound categories causes the auditory cortex to prioritize the representation of information that is most important for identifying those sounds, even when the person is focused on a different task.

## Introduction

Learners of all ages discover structured perceptual input to achieve their goals, and even simple sights and sounds convey rich information that can flexibly support multiple behaviors. A single utterance simultaneously conveys a talker’s age [1] and socioeconomic class [2], whether she is a stranger or a loved one [3], and if she is requesting her *bill* or her *pill* [4]. A glimpse of her face communicates her trustworthiness [5], emotion [6], and identity [7]. Acoustic characteristics of her footsteps can convey her gender [8], identity [9], health, and weight [10]. These underlying patterns are carried across multiple perceptual dimensions and – especially in the case of sound – often require integration of information across time. Successful perception thus requires that we discover and learn distinct input patterns that best inform specific judgments. Flexibility in weighting the most diagnostic patterns of perceptual cues or dimensions for the task at hand is crucial for effective face and voice identification, object recognition, and at least in humans, speech comprehension.

Prominent theories of categorization have posited that learning may drive attention to be directed toward diagnostic perceptual dimensions in a task-dependent manner (e.g., [11–13]). These learned attentional biases are proposed to weight perceptual dimensions most relevant to oft-performed tasks and behaviors [14–16], and to language-specific cues [17–19]. For complex skills like spoken language and object recognition, it can take years or even decades to learn which perceptual dimensions are informative for a given behavioral goal, and to optimally weight or attend to them (e.g., Creel et al. [20–25]). Learners also progress along different trajectories to reach equivalent performance outcomes [26,27]. Indeed, distinct perceptual and neural strategies are reflected in developmental neuroimaging studies that demonstrate that there can be quite different weightings of neural activation across brain regions, even among children or adults who perform similarly on a task (e.g., Brown et al. [28]; Stiles et al. [29]; Krishnan et al. [30]; Yates et al. [31,32]). In auditory categorization tasks, both mouse [33,34] and human [35–37] learners exhibit idiosyncratic patterns of behavior over short-term training. These individual differences may in turn reflect where participants are positioned in a longer-term learning trajectory, over which diagnostic input dimensions are discovered and tuned through experience.

Previous functional MRI work has shown that short-term category training can shape distributed visual (Folstein et al. [12,38]) and auditory (Ley et al. [39]; but cf. Kanber et al. [22]) cortical representations. Here, we establish whether learning a new behavior across an informationally rich perceptual space (a) drives distinct patterns of cortical activity that are spatially predictable given the cortical mapping of the category-diagnostic dimension, and (b) does so in a way that is consistent with acquired attention to the perceptual dimensions most diagnostic to the task. By examining cohorts of learners who reach equivalent levels of expertise in this new domain, we also ask whether patterns of cortical activity reflect the distinct trajectories learners take to achieve expertise. To do so, we adopt a prospective learning approach in which naïve adults learn to categorize entirely novel sounds drawn from a complex, multidimensional perceptual space composed of over 36,000 sounds that vary across complex, not-readily-verbalizable acoustic dimensions [40]. Both the sound exemplars and the categories we have defined across them are entirely novel to listeners, as are the visual icons used during training.

The alien sounds possess information in two simultaneous, non-overlapping frequency bands, with each band having highly similar and complex acoustic information. Each of four aliens’ identities is conveyed by spectrotemporal patterns within one or the other of these frequency bands, with the other band conveying simultaneous but non-diagnostic acoustic information. This allows us to take advantage of the tonotopic mapping of auditory cortex, where we expect acoustic information in each frequency band to be preferentially processed in different spatial subregions of the multiple tonotopic maps that lie across the superior temporal lobe.

Within these maps, functional MRI studies have shown that stable, stimulus-driven voxelwise frequency selectivity can be established across much of auditory cortex [41]. Voxels’ relative frequency preference can also be mapped by *explicit attention to specific frequency bands* within a complex, dynamic, and full-spectrum acoustic scene [42–45]. For example, the degree to which activity in cortex can be similarly driven by attention to a particular frequency band, and ‘bottom up’ acoustic stimulation in that same frequency band can be assessed by constructing ‘concordance maps’ [43]. These maps quantify the similarity of stimulus- and attention-driven tuning of the subset of voxels within small parcellation units within auditory cortex. Such concordance maps allow one to make group-level inferences about *where* there are consistent relationships between stimulus- and attention-driven frequency tuning in auditory cortex, while taking into account the known cross-participant variability in the exact position, size, and orientation of tonotopic maps relative to gyral morphology [41]. We use this analytic approach to ask whether, like explicit attention, the presence of category-diagnostic information in voxels’ preferred frequency band drives relatively greater activation compared to when category-diagnostic information is conveyed via the less-preferred frequency band. Such cross-task similarity would support hypotheses that learning and expertise implicitly drive attention to be directed toward category-informative dimensions [46–48].

As noted above, many naturally occurring sounds convey information about multiple types or categories of information, and this information is conveyed along different acoustic dimensions [49]. To model this, we created all sound exemplars to convey both ‘*alien identity*’ via variable, but statistically structured acoustic patterns embedded within one of two frequency bands, and to simultaneously convey *‘alien size*’ via perceived loudness. Here, lower amplitude sounds are diagnostic of ‘small aliens’ and higher amplitude sounds are diagnostic of ‘big aliens’. Our main group of participants trained over five days to learn alien identity (across spectrotemporal patterns) and also trained to categorize the same sound inventory according to alien size (across amplitude). Another smaller group trained *only* on alien size (conveyed via amplitude) thereby not gaining expertise in classifying alien identity according to the patterned spectrotemporal information embedded in one of the two frequency bands.

This approach allows us to answer several central, yet open, questions: Is category-relevant perceptual information prioritized relative to simultaneously presented category-irrelevant information? If so, does this selectivity persist when the diagnostic dimension is irrelevant to the task at hand? Do emergent patterns of prioritization align closely with neural activity associated with explicit, sustained attention to the dimension as conveyed by different sounds? Finally, do these patterns of neural activity relate to the distinct trajectories that learners take in reaching expert-level categorization?

## Results

A group of young adults (N = 95, Fig 1A) trained for 5 days to develop expertise in rapidly and accurately categorizing complex, multidimensional sounds they had never previously encountered (Fig 2). In the main ‘alien identity’ training task, four sound categories were associated with the identity of four distinct ‘alien’ images (Fig 1C). Each sound possessed acoustic energy in two non-overlapping acoustic frequency bands (~80-750 Hz and ~1000-9000 Hz), each populated with three 400-ms ‘chirps’ played one after another and varying in frequency contour (Fig 2A). In the *category-diagnostic* band, these acoustically variable chirps possessed an underlying regularity that defined category membership (alien identity). Specifically, each of the three chirps in the category-diagnostic band was derived from the pitch contours extracted from a different instance of the same Mandarin tone category (see **Methods-Stimuli**). Simultaneous and temporally aligned chirps in the other, *non-diagnostic* frequency band were also acoustically variable but possessed no coherent regularity and were not predictive of alien identity (Fig 2B). Each of the three chirps in the non-diagnostic band was derived from pitch contours extracted from three different Mandarin tone categories (see **Methods-Stimuli**).

**Fig 1:**
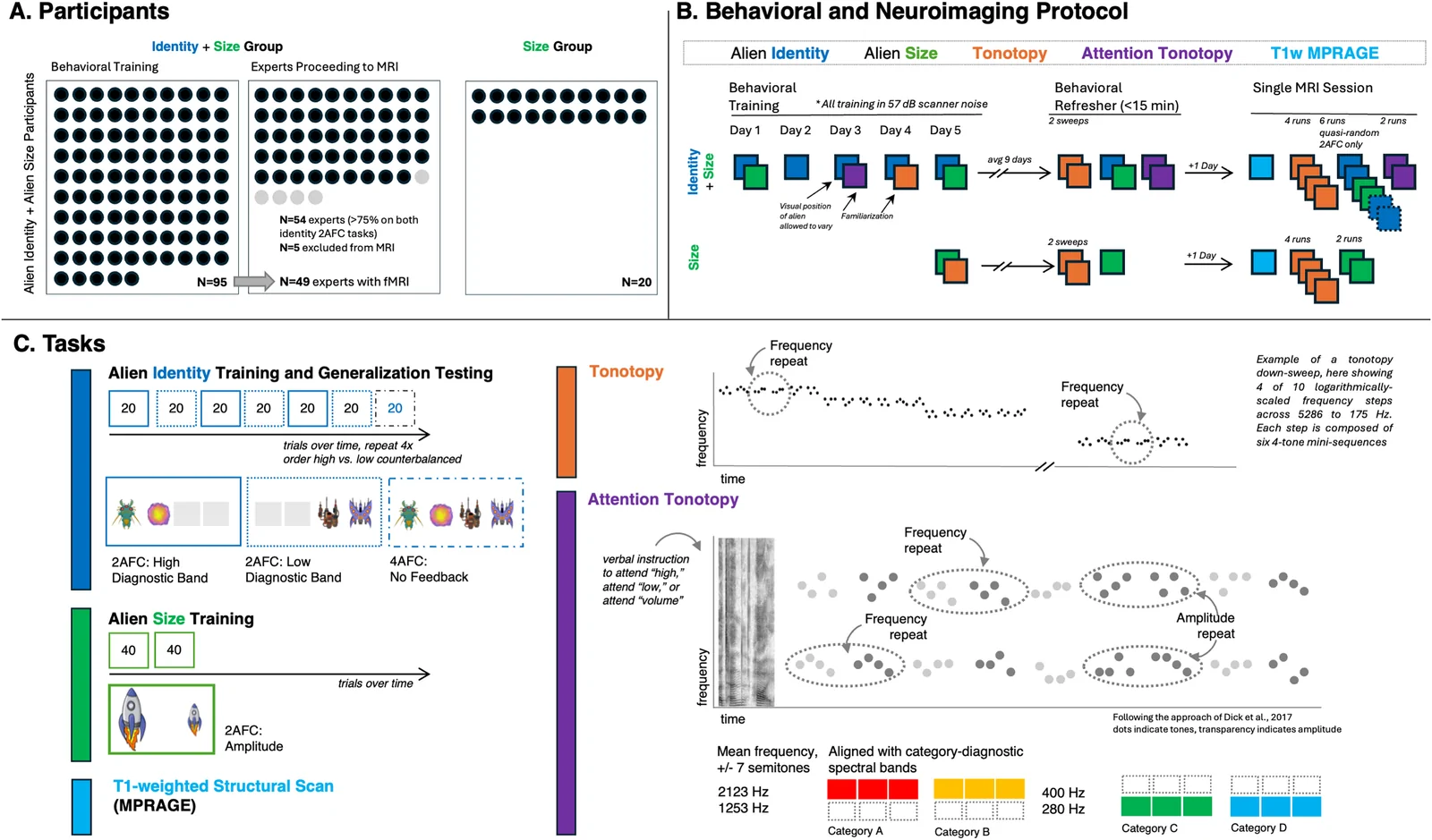
Experimental Approach. (**A**) **Participants**. An *Alien Identity+Size* group of participants (N = 95) trained to achieve expert performance on both alien identity and alien size categorization tasks. Across five days of training with feedback, they learned to associate sound exemplars with four alien identities on a two-alternative forced choice (2AFC) categorization task blocked by high- and low-frequency diagnostic frequency bands (see Fig 2). The N = 54 *Alien Identity+Size* participants who achieved >75% accuracy by Day 5 were recruited as categorization experts for a single fMRI session (5 participants omitted due to motion or noncompliance, for a final fMRI N = 49). A separate *Alien Size* group (N = 20) trained only on the size judgment task *without* training on alien identity, and participated in a single fMRI session. **(B, C)** **Behavioral and Neuroimaging Protocol, Tasks.** Panel **B** summarizes the behavioral and neuroimaging protocol, with Panel **C** as a color-coded legend that schematizes the component tasks. Tasks are described in detail in **Methods-Procedure**. Stimuli are illustrated in Fig 2 and described in **Methods-Stimuli**.

**Fig 2:**
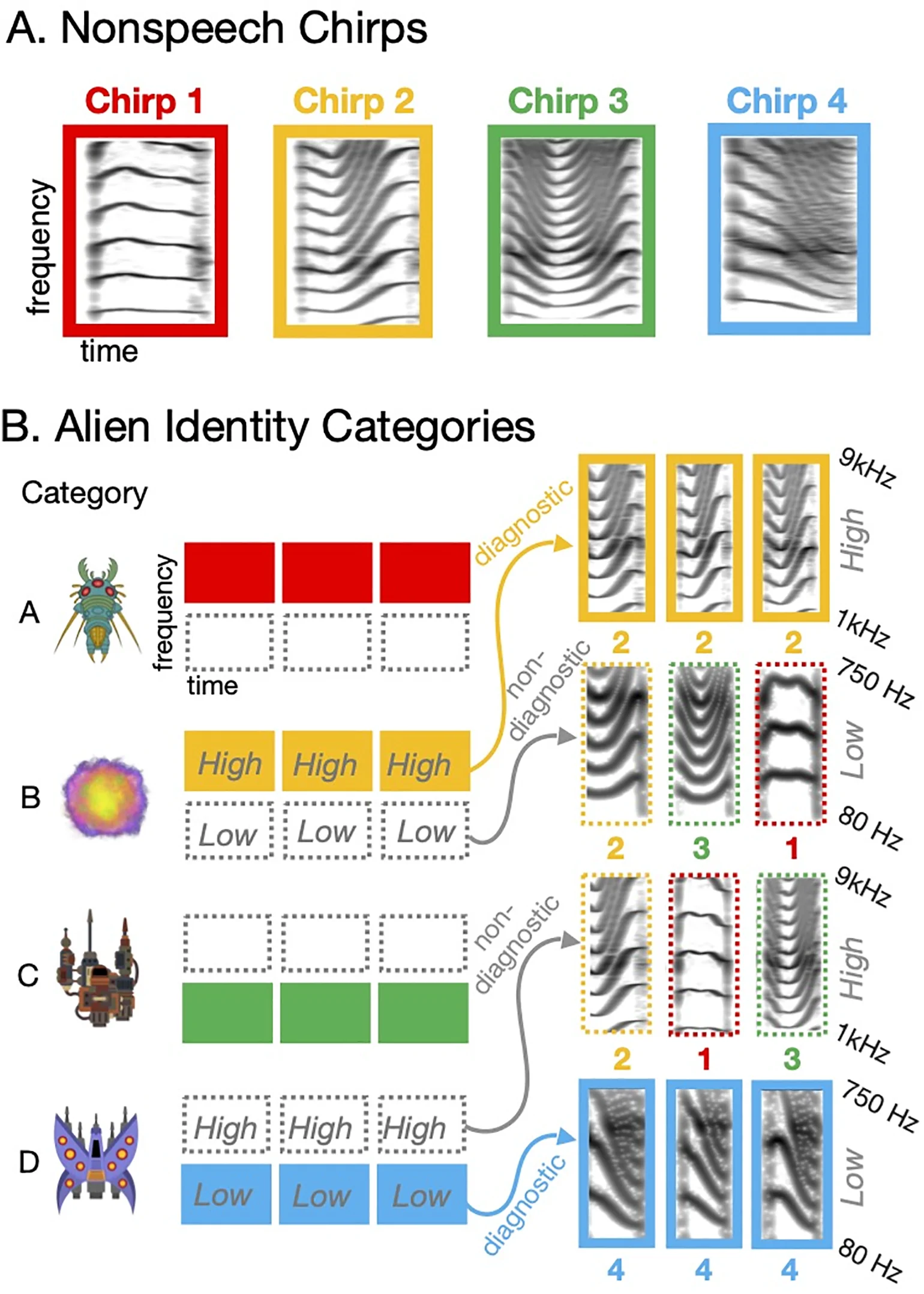
Stimuli. (**A**) **Nonspeech Chirps**. We extracted the fundamental frequency (F0) contour from natural speech recordings of single-syllable words varying in Mandarin lexical tone contour across four (2 female) native Mandarin speakers. This yielded 80 acoustically unique chirps from each of the four classes of Mandarin lexical tone contour (roughly: level, rising, dipping or falling; see Obasih et al. [40]). Spectrograms show one representative chirp from each class (indicated by the colored frame). **(B) Alien Identity Categories.** We next shifted each nonspeech chirp into two non-overlapping frequency bands (low: ~ 80-750 Hz, high: ~ 1,000-9,000 Hz), creating two sets of chirps that served as building blocks for constructing novel auditory category exemplars. Each category exemplar was built from 6 unique chirps (3 high, 3 low, presented simultaneously), represented as rectangles. Color indicates the chirp class shown in **(A)**, with three unique chirps that share the same underlying regularity (derived from a single Mandarin tone category). This *category-diagnostic* pattern conveys alien identity through its association with feedback in training. Three simultaneous chirps (uncolored rectangles) from three different randomly selected chirp classes (derived from different Mandarin tone categories) varied such that there was no structured regularity across the triplet; this frequency band was irrelevant to alien identity. For Categories A and B, the high frequency band was diagnostic; for Categories C and D the low frequency band was diagnostic. The insets on the right show a detailed example from Category B (high-band-diagnostic, Chirp 2 structure) and Category D (low-band-diagnostic, Chirp 4 structure) as time x frequency spectrograms with color frames indicating chirp class for visualization. Spectrotemporal modulation characteristics were extremely similar across simultaneously presented low and high frequency bands and between category-diagnostic and non-diagnostic chirp triplets within the bands. (Note that the spectrotemporal characteristics of the chirps displayed in the figure (such as harmonic density and pitch contour) vary considerably across exemplars; for full details, see Obasih et al. [40] and the full set of experimental materials available online.).

These stimulus characteristics impose several key demands on learning: (1) because chirp triplets appear simultaneously in two distinct frequency bands but statistically structured patterns diagnostic of alien identity occur in only *one* band, listeners must discover the relevant regularity in the context of competing sounds; (2) chirp triplets within the category-diagnostic frequency band are acoustically variable yet possess an underlying spectrotemporal regularity that maps onto alien identity through feedback; (3) because this regularity differs across aliens, listeners must not only identify the diagnostic frequency band but also learn to map specific spectrotemporal patterns within each frequency band onto alien identity; (4) overall amplitude varies across exemplars, such that learning alien identity requires generalization across identity-irrelevant amplitude variation; (5) learning takes place under adverse listening conditions, with sounds embedded in scanner noise throughout, including during behavioral training outside the scanner.

These demands capture central challenges of learning real-world auditory categories. Speech, for example, is characterized by multiple simultaneous acoustic dimensions that may be relevant to one task (did I hear ‘*bear*’ or ‘*pear*’?) yet irrelevant to another performed across the same signal (was the speaker frightened?). Speech is also typically learned in naturalistic conditions that include task-irrelevant ambient noise.

These learning demands allow us to test whether, in experienced listeners, category-relevant perceptual information is prioritized over simultaneously presented but uninformative information. We map frequency selectivity across auditory cortex to identify cortical regions sensitive to the frequency bands populated by category-diagnostic versus non-diagnostic chirp triplets. Because spectrotemporal modulation is highly similar across the simultaneously presented low- and high-frequency bands, we expect comparable bottom-up acoustic activation in corresponding frequency-selective regions of auditory cortex. We ask whether learning how frequency-band-specific patterns map onto alien identity in the presence of competing sounds in the opposing band may lead to an exaggerated neural response in the identity-diagnostic band relative to the non-diagnostic band. We further ask whether neural responses elicited by these alien sounds differ between expert listeners and listeners naïve to alien identity (the *Alien Size* group). Finally, we test whether expertise-related modulation of neural activity parallels patterns of frequency-selective attention observed during overt, voluntary attention to a specific band, thereby directly testing the hypothesis that learning and expertise implicitly bias attention toward category-informative dimensions.

### Category expertise develops across training, with generalization to novel exemplars

Alien identity categorization training involved a two-alternative forced-choice (2AFC) task, with trials blocked by categories with high- versus low-frequency category-diagnostic information. Trial-by-trial feedback indicated the correct alien identity category (Fig 1C, see **Methods-Procedure**). After each training block, a four-alternative forced-choice (4AFC) task with no feedback assessed generalization of category learning to novel exemplars not experienced in training (Fig 1C).

A separate training task involved categorizing exemplars drawn from all four alien identity categories as ‘big’ or ‘small’ according to stimulus amplitude (which varied equiprobably along a continuum from 62−80 dB), *without* regard to the spectrotemporal dimensions associated with alien identity (Fig 1C, see **Methods-Procedure;** all reported dB referenced to 2*10^−5^ and measured in Praat; Boersma [50]). Two independent samples of participants completed alien size category training, one learning alien identity and size categories (*Alien Identity+Size*) and the other learning only size categories (*Alien Size*).

This permitted us to test (a) whether identity-training-induced changes in cortical activation associated with the frequency band carrying identity-diagnostic information exist; (b) whether these changes are lacking in identity-naïve listeners; and (c) whether they persist when task demands are redirected to category decisions based on amplitude. The *Alien Size* group is also important for identifying any bottom-up salience differences that may differentiate sounds from specific alien identity categories, independent of category expertise. By design, the stimulus exemplars (and their order) in the identity and size categorization blocks during the fMRI session were identical to avoid potential stimulus confounds.

A subset of expert *Alien Identity+Size* participants (N = 49) who achieved at least 75% 2AFC accuracy in reporting alien identity across both high- and low-frequency diagnostic bands by Day 5 returned an average of 9 days later for a single functional magnetic resonance imaging (fMRI) session (Fig 1A, 1B). Overall, these experts exhibited above-chance 2AFC alien identity training performance on Day 1, t(48)=15.7, p = 7.35 x 10^−21^, with improvements from Day 1 to Day 5, t(48) = 7.82, p = 2.03 x 10^−10^, to reach near-ceiling alien identity categorization (95.5%; SE = 0.7%, Fig 3A**, left panel**). Among these experts, 4AFC generalization to novel exemplars exceeded chance on Day 1, t(48)=5.64, p = 4.41 x 10^−7^; M = 68.5%, SE = 3.3%) and improved significantly from Day 1 to Day 5 (M = 87.7%, SE = 1.9%, t(48) = 6.56, p = 1.73 x 10^−8^; Fig 3A**, middle panel**). Confusion matrices (Fig 3B) from the 4AFC generalization task on the first and last day of training show that category identification sharpens over training. On Day 1, expert listeners made errors in identifying novel exemplars of all categories and tended to confuse novel exemplars that shared a diagnostic band. By Day 5, listeners’ categorization of novel alien exemplars had sharpened considerably across the board. In addition, the learning trajectories across which alien identity experts reached criterion expertise were heterogeneous; we used this variability to subgroup learners for further analysis (see below).

**Fig 3:**
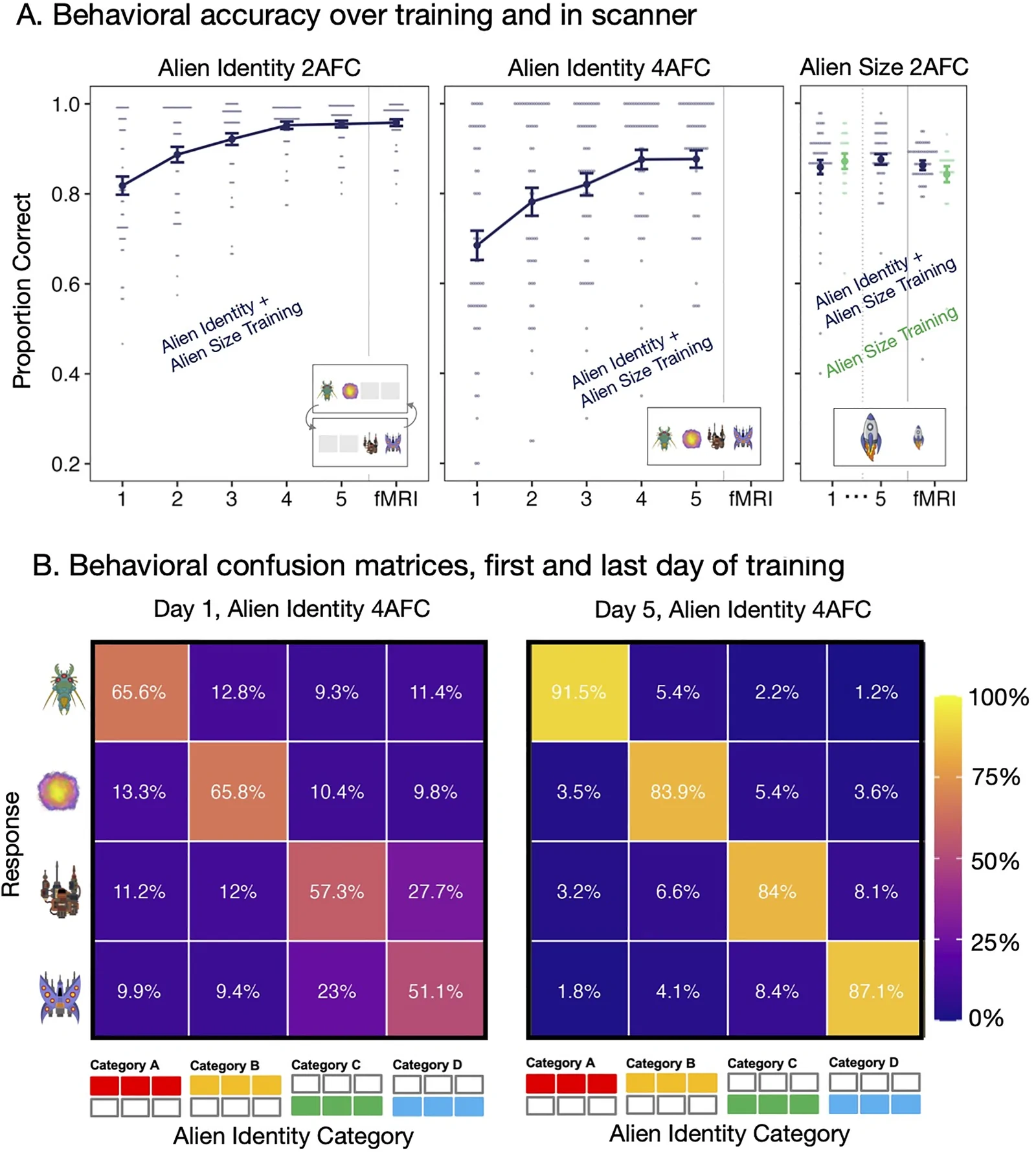
Behavioral Data. **(A)** Alien Identity Training (2AFC with feedback). Proportion correct 2AFC categorization across Days 1-5 (and in the fMRI scanner, with no feedback) for the N = 49 *Alien Identity+Size* participants who achieved 75% accuracy across all alien identity categories by Day 5. **Alien Identity Generalization (4AFC no feedback).** The same *Alien Identity+Size* participants’ 4AFC categorization of novel category exemplars improved across Days 1-5. **Alien Size Training (2AFC with feedback).** A separate training task involved categorizing exemplars drawn from all 4 alien identity categories as ‘big’ or ‘small’ according to stimulus amplitude. The *Alien Identity+Size* participants who trained on alien identity trained on alien size on Day 1 and Day 5 of training, and in the scanner without feedback. A separate *Alien Size* group (N = 20) never trained on alien identity; this group trained on alien size (defined by amplitude) for one day and performed the same task in the scanner with no feedback. **(B) Confusions in 4AFC Alien Identity Generalization.** Confusion matrices comparing category ground truth with response for Day 1 vs. Day 5 reveal increasing category generalization and specificity. Like Fig 2, x-axis rectangles for confusion matrices show diagnostic frequency band for each category (warm colors: high band, cool colors: low band). The data and scripts underlying this figure can be found at https://doi.org/10.5522/04/33974425, and direct links for Fig 3A and 3B are as follows: https://rdr.ucl.ac.uk/ndownloader/files/69528888 (Fig 3A-Panel1-SourceData); https://rdr.ucl.ac.uk/ndownloader/files/69528885 (Fig 3A-Panel2-SourceData); https://rdr.ucl.ac.uk/ndownloader/files/69528894 (Fig 3A-Panel3-SourceData); https://rdr.ucl.ac.uk/ndownloader/files/69528882 (Fig 3B-SourceData).

Alien identity experts also trained on the alien size task on Days 1 and 5 to a similar level of accuracy (M = 87.5%, SE = 1.2%; Fig 3A**, right panel**). The *Alien Size* participants (N = 20, sample size matched to the highest-performing ‘Early Expert’ subgroup of *Alien Identity+Size* group, see below) achieved 87.1% (SE = 1.7%) alien size categorization accuracy at the end of one training session (Fig 3A, **right panel**).

Both groups took part in a single fMRI session following training. In-scanner behavior echoed patterns of training performance (Fig 3A, **right panel)**. Those who trained on both alien identity and alien size successfully categorized alien identity according to patterns evolving in the diagnostic frequency bands (2AFC task, M = 95.8%, SE = 0.7%) and according to stimulus amplitude in the size task (M = 86.2%, SE = 1.1%). Participants trained only on alien size performed with similar accuracy (on alien size) during scanning (M = 84.2%, SE = 1.8%), compared to the group trained on both tasks.

### Alien identity learning drives frequency-band-selective activation differences in tonotopic cortex

We first present functional MRI data from the *Alien Identity+Size* participant group trained on both alien identity and alien size tasks. To identify regions likely to differentially respond to category-diagnostic frequency bands, and to test differences in neural responses as a function of categorization task demands, we first characterized each participant’s unique tonotopic organization on a voxel-wise basis across bilateral auditory-responsive cortex. Here, participants listened to concatenated series of 4-tone mini-sequences that stepped periodically over a 60-semitone range (175–5,286 Hz, two runs ascending and two descending, see Dick et al. [43] and Fig 1C, **Methods-Procedure**). To maintain task focus, participants performed a 1-back task, reporting infrequent mini-sequence repeats (in-scanner d’: M = 3.55, SE = 0.08). From these data we compute voxel-wise activation to mini-sequences grouped into the low and high frequency bands that conveyed alien identity information. The difference in beta coefficients across high-minus-low frequency bands quantifies voxels’ frequency selectivity. As expected from prior work [41], we observe coarse alignment of tonotopic organization across individuals (S1 Fig), but cross-individual heterogeneity in the mosaic arrangement of fine-grained frequency selectivity.

The ‘concordance map’ approach [43] described in the introduction allows us to capture consistent regional differences in responses across participants while retaining frequency sensitivity at a voxel-wise level, despite tonotopic map inter-subject heterogeneity. Here, each individual’s auditory-responsive cortices are parcellated into a set of small, roughly equal-area ROIs that are projected via curvature-aligned spherical registration from a participant surface in Dick et al. [43], onto each individual’s auditory cortical surface (Fig 4A, see **Methods-Analyses**). (The ROI borders are not designed to align to gyral anatomy or functional fields). Each ROI exhibiting frequency selectivity at the group level (as estimated using the group-average tonotopic map) is sampled into the cortical ribbon in the individual’s native volumetric EPI space (Fig 4B). Analogous to the high-minus-low frequency selectivity contrast from the tonotopy runs, voxel-by-voxel beta coefficients relating to activation during alien identity categorization (involving the high- and low-frequency category-diagnostic bands) are computed and a high-minus-low frequency band difference in beta coefficients is calculated for each voxel. These parallel difference scores permit us to fit a regression model across all participants within each ROI (with participant as a random factor, Fig 4C) revealing the ROI-wise concordance between the voxelwise frequency selectivity evoked by tonotopy stimuli, and the hypothesized, category-specific frequency-band selectivity driven by the alien identity learning task (Fig 4D and **Methods-Analyses**). (All of the inferential statistics for each subgroup, as well as cross-subgroup comparisons, are reflected in the statistical maps, with outlines showing FDR-corrected significance in the same way as with the full groups).

**Fig 4:**
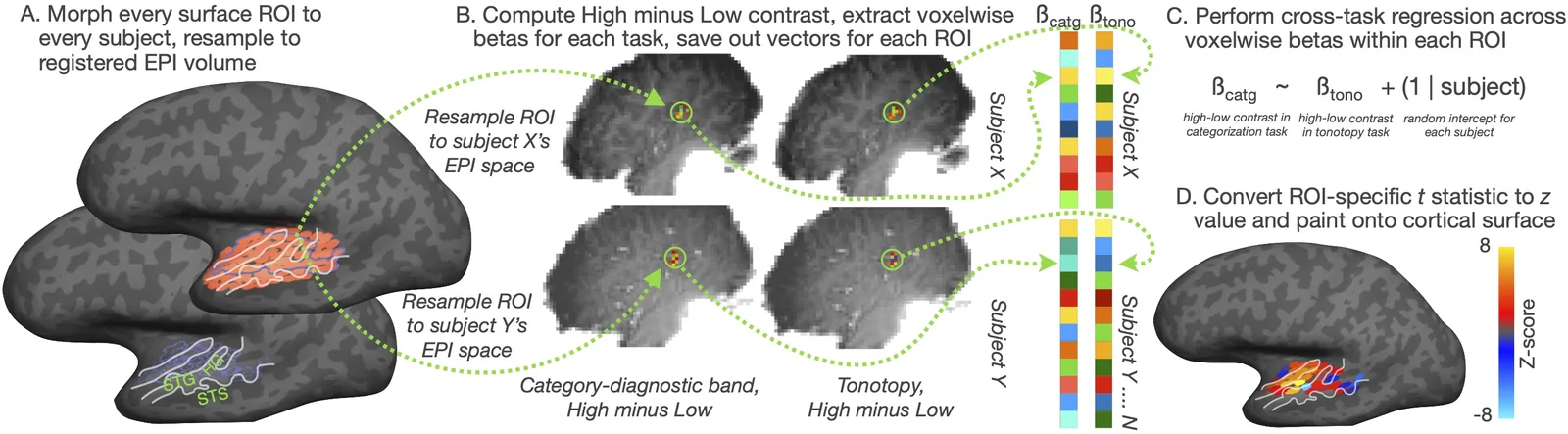
Approach to concordance analyses. To quantify the similarity in activation patterns across tasks, we defined a set of regions of interest (ROIs) and conducted cross-task concordance analyses within each ROI. **(A)** First, we took a set of cortical surface ROIs tiling the superior temporal plane; the ROIs were created to be roughly equivalent in area, with borders quasi-arbitrarily positioned relative to cortical landmarks (ROIs shown in orange; Dick et al. [43]). For the current work, we only considered ROIs that fell within tonotopically organized auditory cortex as determined by a group average map; these ROIs are outlined in purple, with gyral boundaries traced in white and anatomical labels indicated in green (HG: Heschl’s Gyrus; STG: Superior Temporal Gyrus; STS: Superior Temporal Sulcus). Each ROI was morphed via cortical-surface-based registration to the individual participant’s surface and then sampled to their native volumetric EPI space. **(B)** For the two categorization tasks, we always examined the contrast between the high-band-diagnostic and the low-band-diagnostic identity categories. We used this contrast even when the task was to categorize stimuli based on overall amplitude, because we hypothesized that, for the *Alien Identity+Size* group, relatively intensive training would result in acquired attentional gain for the identity-diagnostic frequency band, even when performing an unrelated task. For the tonotopy task, we used the contrast between bottom-up sensory input from high vs. low frequencies, and for the attention-tonotopy task, we used the contrast between explicit, directed attention to high vs. low frequencies in the context of bottom-up sensory input from both frequency bands. Within each volumetric ROI, we extracted voxel-by-voxel beta coefficients for the high vs. low contrast in each task. **(C)** To assess the similarity of activation across tasks, we submitted voxelwise betas to a regression analysis for each ROI, separately; the goal in each regression analysis was to predict the beta coefficients from one task using the betas from another. Regressions included by-subject random intercepts; note that each participant will contribute slightly different number of voxels to each regression model due to interindividual anatomical differences. **(D)** The resultant ROI z-statistic, which indicates the strength and direction of the relationship between the two sets of beta coefficients, was painted on a single cortical surface for interpretation and comparison with other maps.

In other words, this approach allows us to ask whether categorization decisions guided by chirp patterns embedded in high versus low frequency bands yield activation in corresponding frequency-selective cortical regions. Since each category exemplar possesses rich acoustic energy in each frequency band as well as amplitude variation (signaling size), an exaggerated neural response in the identity-diagnostic versus non-diagnostic frequency band provides evidence for expertise-driven changes in cortical activation to the category exemplars.

We first established that performing category tasks with these novel ‘alien’ sounds evoked broad activation across much of auditory and auditory-related cortex bilaterally, as well as across a number of other brain networks and regions, including inferior, prefrontal, inferior parietal, and ventral and lateral occipital cortex (Fig 5, **top**). We then turned to the ROI-based regression approach using concordance maps. (Note that all maps show group averages or group comparisons, and do not include any behavior-activation correlation measures). As seen in Fig 5B, we found that, across multiple ROIs, categorization decisions requiring use of information in high versus low frequency bands drives differential activation in voxels exhibiting preferential responses to these acoustic frequencies. Most left hemisphere (LH) auditory cortical ROIs along the swath of the planum temporale – from Heschl’s gyrus posteriorly to the anterior-most aspect – show a strong positive concordance between experience-dependent and category-diagnostic versus stimulus-evoked voxelwise frequency selectivity. Some LH posterolateral ROIs along the superior temporal gyrus (STG) also show positive concordance. In right hemisphere (RH) auditory cortex, we observe significant positive cross-task concordance in multiple clusters of ROIs; in general, FDR-corrected groupings are separated by ROIs showing positive but subthreshold category-tonotopy associations. Across both hemispheres, the few ROIs with negative relationships across tasks tended to be situated in regions exhibiting relatively weak group-level tonotopic selectivity (S1 Fig).

**Fig 5:**
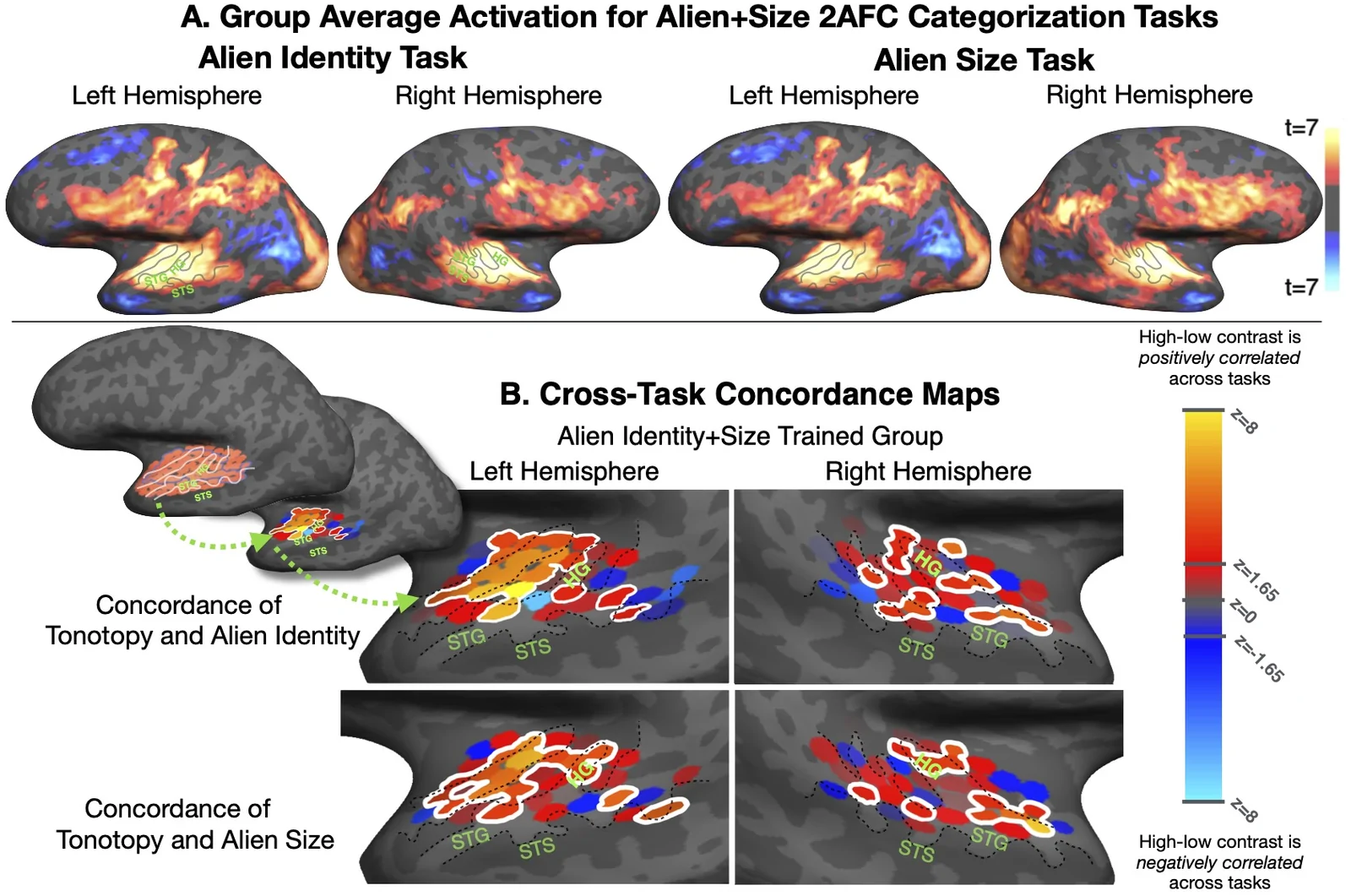
Group average activation and cross-task concordance maps. **(A)** Cortical-surface-based group averages of BOLD response to the alien identity and alien size 2AFC categorization tasks compared to resting baseline, for *Alien Identity+Size* experts. Average brain-wise responses to both tasks with the auditory category exemplars show extensive bilateral activation of auditory-related cortex in temporal, inferior parietal, and frontal regions, as well as lateral occipital cortical regions typically associated with visual object recognition. The activation maps are leniently thresholded at a t > 2 to show full activation patterns. The colorbar shows mapping of t-statistics; the grey tracing shows underlying curvature patterns, and anatomical labels are as in Fig 4. **(B)** Following the approach illustrated in Fig 4, we assessed the concordance among the tonotopy, frequency-diagnostic identity categorization and amplitude-diagnostic size categorization tasks. Each inset figure shows a blow-up of a given concordance map rendered on the left or right temporal lobe. In general, there was a strong positive relationship among betas, indicated by warm-colored ROIs. ROIs outlined in white are statistically significant (p < 0.05 after applying FDR correction, see **Methods-Analyses**; dotted black tracing shows underlying curvature patterns). The top row shows that there is greater cortical activation for category-diagnostic, compared to non-diagnostic, frequency bands during alien identity categorization among the *Alien Identity+Size* group. The second row shows that this pattern of activation persists even when categorization according to alien size demands reliance on an orthogonal dimension (amplitude). The bottom row shows the concordance map between alien identity and alien size tasks. Note that all maps show group averages or group comparisons, and do not include any behavior-activation correlation measure. The summary data used for this figure are available at https://doi.org/10.5522/04/33974425.

Thus, across expert participants actively engaged in the alien identity category task, we observe that voxels in multiple auditory regions show greater cortical activation associated with the category-diagnostic versus non-diagnostic frequency band in a way that is predicted by the same voxels’ stimulus-driven preferences for one or the other frequency band.

### Greater cortical activation in voxels preferring category-diagnostic frequency bands persists even in behavioral contexts that do not demand this information

We hypothesized that learning may cause identity-diagnostic dimensions to acquire salience, such that frequency-selective recruitment may persist even when task demands shift to categorization across an orthogonal dimension. To test this, in different blocks, participants also categorized exemplars from all four alien categories according to whether they were ‘big’ or ‘small’ aliens based on amplitude. (Note that sound exemplars varied in amplitude in both alien identity and size tasks, but amplitude was task-relevant only in the size task). As shown in Fig 3A **(right panel)**, in-scanner alien size categorization was accurate, but well below ceiling performance. This indicates that participants successfully relied upon the amplitude dimension for categorization, and that the alien size task was non-trivial.

Although task demands directed participants’ overt attention to an orthogonal acoustic dimension (amplitude), concordance between voxels’ stimulus-evoked frequency preference and exemplars’ frequency-diagnostic band for the over-practiced and more challenging *identity* task persisted (Fig 5B). Across all *Alien Identity+Size* participants, in the LH, we observed significant concordance across most of the ROIs in the planum temporale, as well as two ROIs in the posterior STS. In the RH, we observed concordance in three small groups of ROIs in the middle inferior circular sulcus, the anterolateral STG, and two isolated ROIs along the posterior crown of the STG.

The differential cortical activation across the high versus low frequency bands is consistent with an ‘acquired salience’ for category-diagnostic dimensions that reflects stable changes in cortical activation elicited by category exemplars that are persistent across changing task demands. Prioritization of category-diagnostic dimensions is present even in contexts that do not demand reliance on them. This effect is challenging to account for via a ‘bottom-up’ acoustic salience account, as all stimuli possessed complex information in each frequency band, in addition to amplitude variation. Further, results from the repetition suppression fMRI literature would generally suggest that the acoustic difference between the category diagnostic- and non-diagnostic bands - namely the higher-level regularity in pitch contour across the three individual tokens in the diagnostic band – would diminish activation in auditory regions sensitive to that information [51,52], but cf. Segaert et al. [53]). In the present results, we observe the opposite pattern. To examine this question further, in the next two sections we make more direct tests of the impact of alien identity category expertise on activation to the category-diagnostic band.

### Cortical activation mirrors individuals’ trajectories of learning

All *Alien Identity+Size* participants who completed the fMRI scan (N = 49) had achieved high levels of expertise in a novel domain. Yet their paths to expertise varied (Fig 6A). To understand how these learning trajectory differences might affect neural representations, we grouped participants based on the speed and depth of learning over behavioral training. Early Experts (N = 17) achieved at least 90% accuracy on Day 1 for 2AFC training on both high- and low-frequency diagnostic bands. Late Experts (N = 16) started at lower accuracies but showed an accuracy increase of at least 20% over the course of 2AFC training and achieved accuracy similar to the Early Experts at the end of training.

**Fig 6:**
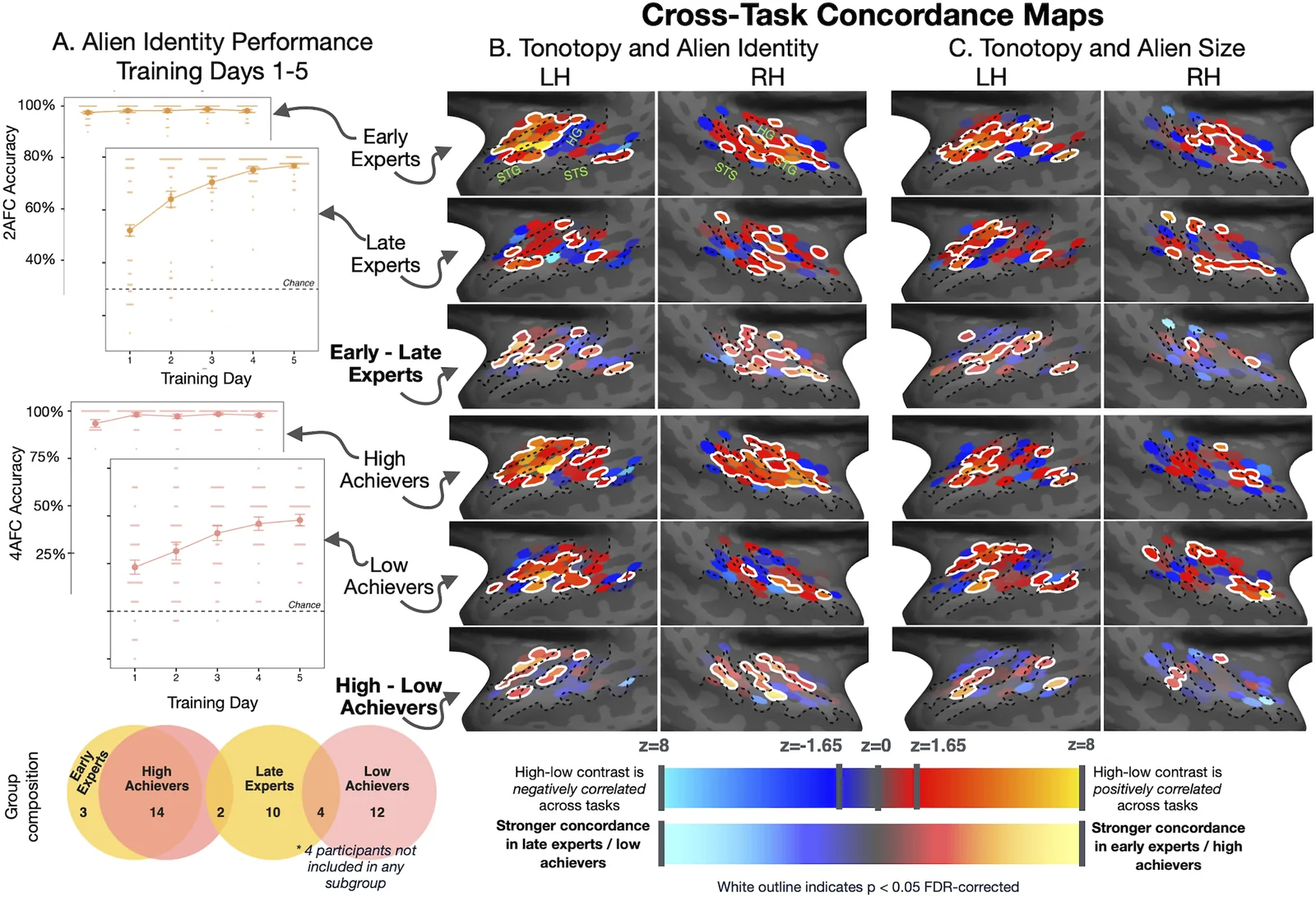
Sub-Group analyses tested for differences in cross-task concordance as a function of frequency-diagnostic categorization performance during the course of training. **(A)** One set of analyses considered differences as a function of *how quickly participants mastered the 2AFC Alien Identity+Size categorization task*: *Early experts* demonstrated mastery for both high-band-diagnostic and low-band-diagnostic categorization on Day 1, whereas *late experts* demonstrated substantial improvement over the course of training. A second set of analyses considered differences as a function of *generalization ability*, separately analyzing data from the top tercile (*highest achievers*) and bottom tercile (*lowest achievers*) on the 4AFC generalization task. Behavioral performance and Venn diagrams for these sub-groups are shown in the left panel. Light dots indicate individual participants (no datapoints are obscured by graph overlay), solid points indicate mean performance for each day, and error bars indicate standard error. **(B-C).** We assessed cross-task concordance between the tonotopy task and the alien identity (**B**) and alien size task **(C)**; the top two rows show Early and Late Experts concordance maps, with the third row showing the difference between Early vs. Late Experts maps. Parallel concordance maps for High and Low Achievers are shown in the bottom three rows. In the difference maps, warm colors indicate ROIs where stronger cross-task concordance was seen in early experts/highest achievers, and cool colors indicate ROIs where stronger cross-task concordance was seen in late experts/lowest achievers. As in Fig 5, z-statistics are shown in the colorscale, the white outline surrounds ROIs with FDR-corrected p-values at p < 0.05 (see also **Methods-Analyses**), and the black dotted outline shows underlying cortical curvature. The data underlying this figure can be found in the repository found at https://doi.org/10.5522/04/33974425; the direct link for the source data in Fig 6A is https://rdr.ucl.ac.uk/ndownloader/files/69528891.

We also estimated ultimate depth and generalizability of learning by creating separate groups based on generalization performance in the 4AFC task, with the High Achievers (N = 16) and Low Achievers (N = 16) defined across the top and bottom generalization terciles. These paired subgroups capture (partially overlapping) sets of participants with distinct learning trajectories.

We observe the fingerprints of these trajectories on subsequent cortical responses to category exemplars (Fig 6B – note that these maps show concordance maps and do not include any measure of correlation between individual participants’ behavioral performance and neural activation). Compared to Late Experts, Early Experts exhibit significantly stronger concordance between alien-identity-categorization-driven and stimulus-driven voxelwise frequency selectivity in ROIs within anterior temporal regions, as well as posterior STG/STS (Fig 6B, **top three panels**). In the RH, Early Experts show significantly greater concordance in ROIs along much of the STG and postero-medially. During the Alien Size task, where spectral band was category-irrelevant, Early Experts also showed significantly stronger concordance in ROIs along much of the medial part of the left STG, with very limited group differences in the right hemisphere.

Similarly, in the Alien Identity task, the High Achievers exhibited significantly greater concordance than the Low Achievers in anterior temporal and STG LH ROIs, with similar group differences in the right hemisphere. (Fig 6B, **bottom three panels**). In the Alien Size task, High and Low Achievers differed less, with High Achievers showing greater anterior and posterior LH STG concordance, as well as in a few posterior STG and one medial anterior ROI in the right hemisphere.

This indicates that, despite their shared ultimate success in learning and in-scanner task performance, and quite similar overall patterns of concordance (as seen in Fig 6B, rows 1,2, 4,5) *Alien Identity+Size* participants’ *degree and rate of learning* of identity category expertise is associated with specific patterns of brain activation that reflect the degree of tuning to category-diagnostic perceptual dimensions. An important caveat to these findings is that these behaviorally-defined subgroups are relatively small compared to the full sample size, so follow-ups with larger N samples will be helpful for interpreting the strength of these effects.

### Learning-driven attentional gain for a diagnostic dimension depends on the demands of learning

As described above, expert-level categorization drove activation of auditory areas tuned to the frequency band diagnostic of alien identity, even when *Alien Identity+Size* experts were successfully categorizing alien size based on stimulus amplitude. To make a critical test of the importance of learning to weight or attend to the identity-diagnostic frequency band in driving these results, we compared the *Alien Size* trained group (N = 20) - which remained naïve to alien identity and *never* trained to learn categories based on frequency-band-delimited acoustic information - to the Early Expert subset (N = 17) of the *Alien Identity+Size* trained group.

As would be expected, alien-size-trained participants exhibited a qualitatively equivalent degree of frequency selectivity and tonotopic organization in auditory cortex (S1 Fig), relative to the Early Expert group that trained on both alien identity and size. They also showed high behavioral accuracy on the tonotopic mapping task (in-scanner d’: M = 2.68, SE = 0.13). Having established typical patterns of tonotopic mapping, we next tested whether alien-size-only-trained participants’ auditory cortex would show differential activation for stimuli with identity-disambiguating information in high and low frequency bands when they were performing the amplitude-based alien size task. If there is a systematic activation preference for the alien-identity-diagnostic frequency band among participants trained only on alien size – particularly with a similar pattern of sensitivity to alien identity experts – this would suggest that the spectrotemporal regularities present in the identity-diagnostic frequency band are driving at least some of the results we have observed with alien identity experts, potentially via learning-induced attentional capture (Zhao et al. [54]).

Though the alien-size-trained participants demonstrated high behavioral accuracy on amplitude-based size categorization during scanning (M: 84.2%, SE: 1.8%, Fig 3A right panel) they exhibited little sign of activation for the identity-diagnostic frequency band that matched underlying tonotopy, with only a few ROIs showing significant concordance, and only in the most peripheral regions that are inconsistently and weakly frequency-selective across participants (Fig 7A). By comparison, as shown previously in Fig 6C (top right panel), the Early Experts trained on both identity and size tasks showed strong concordance across ROIs between the difference in activation to high- versus low-frequency *identity-diagnostic* band stimuli during alien-size categorization blocks, and high- versus low-frequency tone mapping. A direct statistical comparison between the two groups showed that most LH ROIs just medial to and along the STG, and multiple patches in the RH STG and more medially showed significantly greater frequency-selective modulation in the amplitude-conveyed alien size task among Early *Alien Identity+Size* Experts compared to those trained on alien size only (Fig 7B).

**Fig 7:**
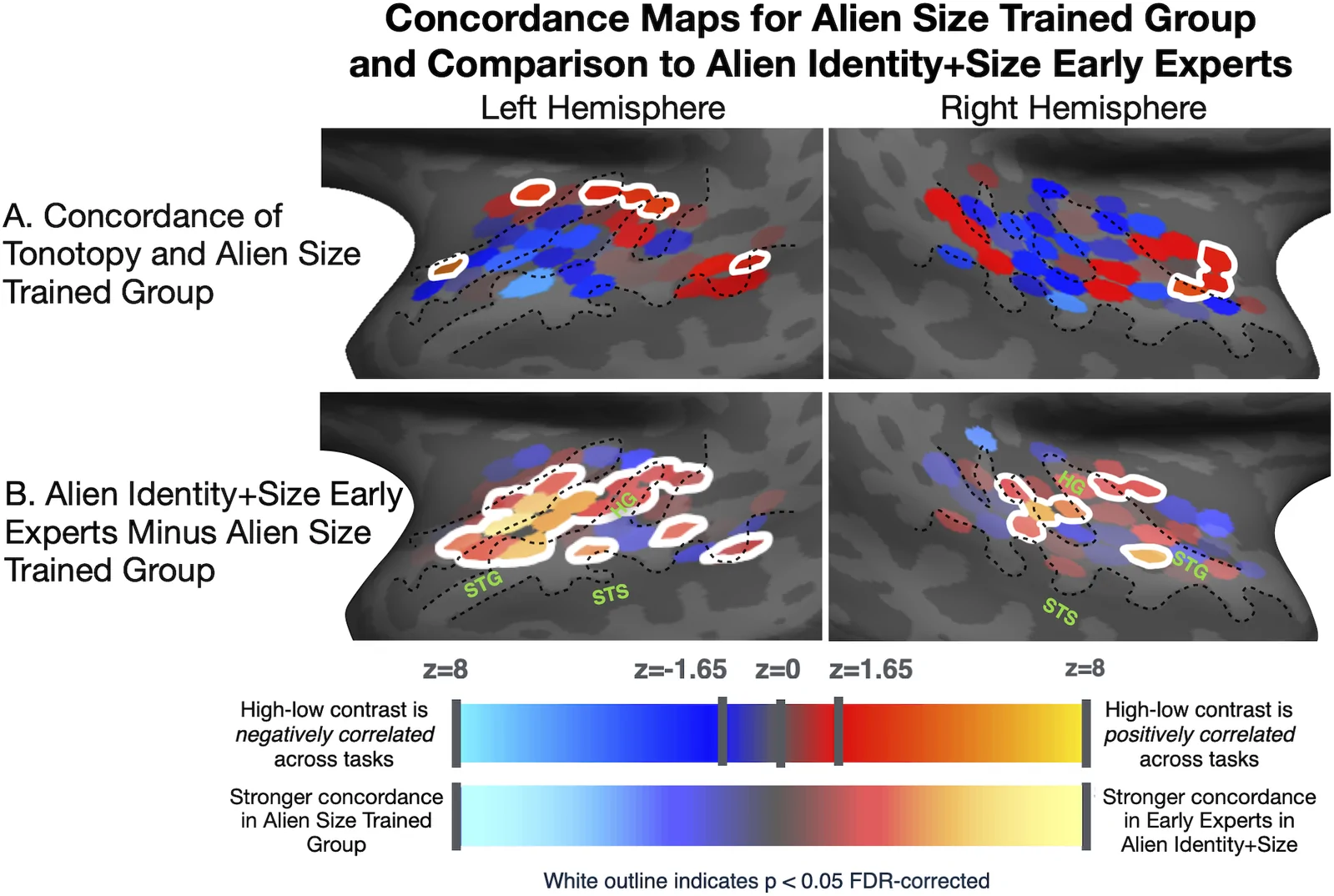
Concordance Maps for *Alien Size* Trained Group and Comparison to Early Experts in *Alien Identity+Size* Trained Group. (**A**) Shows the *Alien Size* trained participants (N = 20) concordance map between high-versus-low-frequency responses from tonotopy, and response differences in the alien size fMRI task when comparing activation for alien sounds with *alien identity* information in high vs. low spectral bands. (Recall that this participant group was naïve to alien identity.) With the exception of a few medial ROIs, participants did not recruit auditory cortex in a frequency-selective way when performing amplitude-based categorization. **(B)** shows the difference between the analogous concordance map for the Early Expert group of *Alien Identity+Size* trained group (top right panel of Fig 6C) vs. the *Alien Size* only trained participants. This difference map suggests that the frequency-selective recruitment of auditory cortex that *Alien Identity+Size* experts exhibited in the amplitude categorization task was not driven by properties of the acoustic signal per se. The summary data used for this figure are available at https://doi.org/10.5522/04/33974425.

To test the generality of this result, we ran the same comparison between the *Alien-Size-Trained* cohort and the complete *Alien Identity+Size* cohort (N = 49). As seen in S2 Fig, we observe a similar difference in concordance maps, where the Alien Identity+Size cohort shows significantly more concordance in most of the LH ROIs from the middle of the STG extending medially, as well as long the posterior STG. In the RH, the greater concordance in the full *Alien Identity+Size* cohort is again very similar to that observed in the comparison of Early *Alien Identity+Size* experts versus Size experts, with greater concordance in multiple ROIs extending laterally to medially from the middle of the STG, as well as in anterior STG. This adds further support for the hypothesis that learning categories that are dependent upon statistically structured auditory information restricted to one frequency band drives the acquired salience of that structured information [55], such that identity categorization experts (but not identity-naïve participants) exhibit frequency-band-specific recruitment of auditory cortex, even when categorizing stimuli along an orthogonal dimension (stimulus amplitude).

### Frequency-selective activation patterns driven by category learning align with those evoked by overt attention

We have observed that when categorization implicitly hinges on information in a delimited frequency band, voxels more responsive to this band show increased activation compared to when diagnostic information is conveyed via the less preferred frequency band, despite simultaneous acoustic energy in each band. Motivated by theoretical accounts suggesting that perceptual weighting reflects attentional gain, we next asked whether the pattern of increases in activation for category-diagnostic frequency bands is aligned with activation evoked by *explicit* attention to the same frequency band. Among participants trained on *Alien Identity+Size*, we compared the activation difference between high- and low-frequency identity-diagnostic bands with the parallel activation differences evoked by explicit, sustained attention to one of two simultaneously presented streams of sinewave tones situated within the same high- and low-frequency bands.

In addition to the tonotopy and alien categorization tasks, the *Alien Identity+Size* participants also completed a sustained auditory selective attention task based on the approach of Dick et al. [43], who found that overt attention directed at one of two sequences of tones positioned in specific frequency bands elicits an attention-driven map across auditory-responsive cortex. As shown in Fig 1C, participants heard two simultaneously presented sequences of sinewave tones composed of concatenated 4-tone mini-sequences that varied in amplitude; mini-sequences were played simultaneously in higher and lower frequency bands, corresponding to the diagnostic bands of the alien identity task (see **Methods-Procedure**). At the beginning of each task block, explicit instructions directed participants to detect mini-sequence repeats in the high frequency band, the low frequency band, or repeats of the overall relative amplitude of the mini-sequence (1-back task d’: M = 2.28, SE = 0.13). As anticipated given previous studies of frequency-selective attention [42–45], voxelwise differences in activation when attending to high versus low frequency bands were strongly associated with stimulus-evoked frequency preference (from the tonotopy scans) across most LH auditory cortex ROIs except in the anterior lateral and posterior STG (Fig 8A). In the RH, spectrally directed attention evoked similar activation differences as tonotopic scans along the lateral planum temporale and STG.

**Fig 8:**
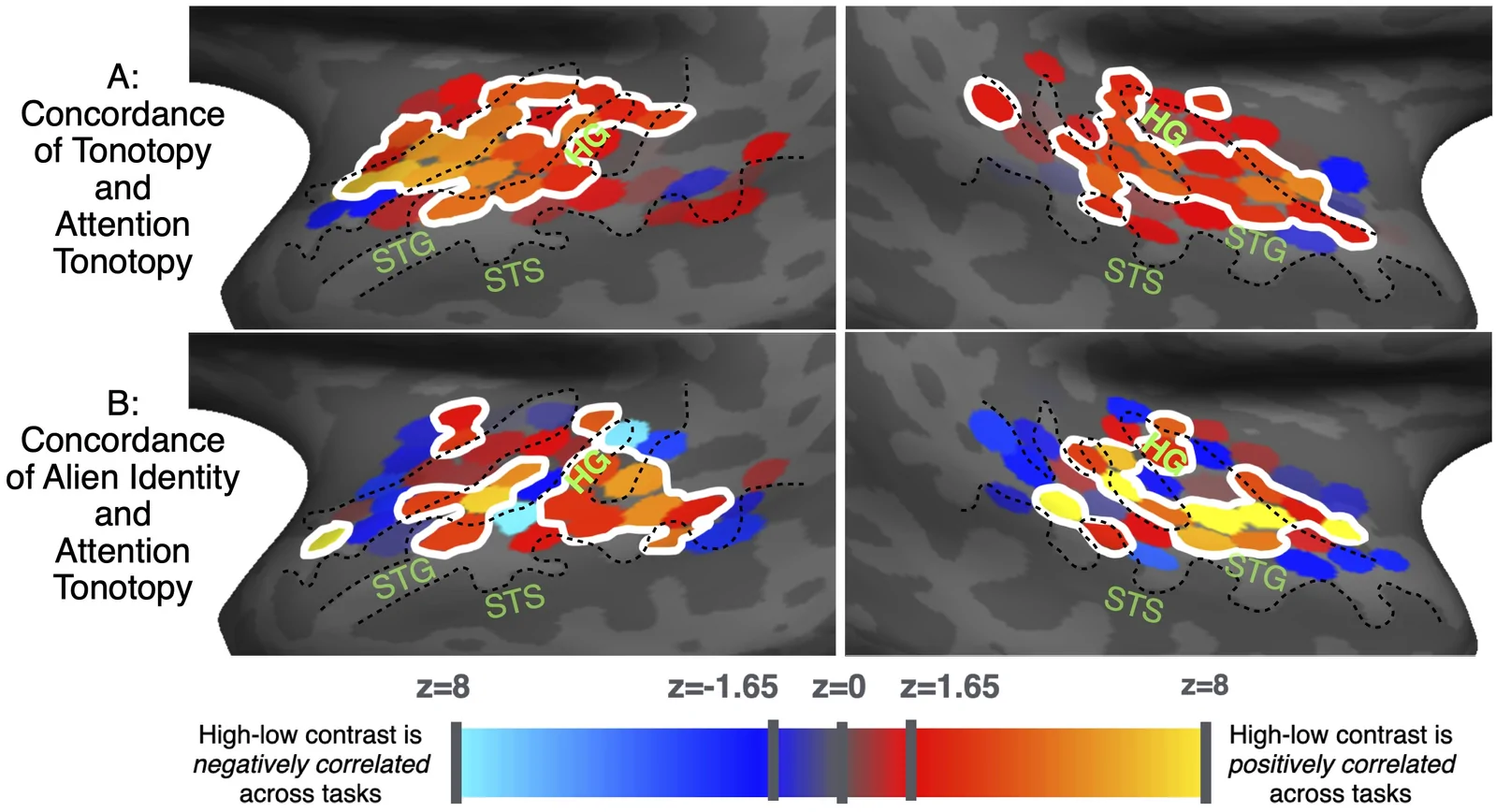
Frequency-selective activation patterns driven by category learning align with those evoked by overt attention. Theoretical accounts suggest that the neural selectivity that emerges with learning is driven by increased attention to diagnostic dimensions. To test this hypothesis, we assessed cross-task concordance with the attention tonotopy task (Fig 1C), where participants were presented with two streams of short tone sequences, and each stream was in a high- or low-frequency band corresponding to the high and low identity-category-diagnostic frequency bands in the *Alien Identity+Size* task. Tone sequences also differed in overall amplitude. Participants were instructed to detect repeated 4-tone sequences in one or the other band or to overall perceived loudness, depending on block. **(A)** Analyses considering all participants showed strong concordance between attention-driven activation to high vs. low frequency bands and the high vs. low frequency band activation difference elicited by the tonotopy task (i.e., stimulus-driven activation). **(B)** Concordance between alien identity categorization-driven activation to high vs. low diagnostic frequency bands, and explicit attention to the same frequency bands populated with simple sequences of tones was observed bilaterally along much of the lateral aspect of tonotopically mappable auditory cortex. The summary data used for this figure are available at https://doi.org/10.5522/04/33974425.

We then asked whether patterns of cortical activation elicited by *explicit, directed attention* to the frequency bands align with activation patterns that *arise in category decisions that are implicitly reliant on information* in these same frequency bands. As shown in Fig 8B, cross-task concordance maps showed that analogous differences in activation to high versus low frequency bands were observed laterally in both hemispheres, in a swath of ROIs along the central portions of the STG and abutting ROIs in the planum temporale.

In summary, lateral aspects of auditory cortex that show differential activation when listeners explicitly attend to a delimited region of the frequency spectrum also show correspondingly differential activation when categorization *implicitly* hinges on information situated in these same frequency bands. This holds true despite the considerable acoustic differences between the sequences of pure tone stimuli used for the explicit attention task and the spectrotemporally more complex and structured alien sound category exemplars.

## Discussion

How does perception come to weight the most diagnostic patterns of input for the task at hand? We used fMRI to probe the neural consequences of learning to categorize across a novel soundscape defined by multiple, complex acoustic dimensions evolving in time. To do this, we situated category-diagnostic patterns into frequency-delimited bands – thereby coarsely segregating category information into different channels in tonotopically organized auditory cortex. This allowed us to make targeted predictions about: (a) how categorization-driven modulation of cortical activity would intersect with existing tonotopic functional regionalization; (b) how different trajectories of learning to expert-level performance would be reflected in these representations; and (c) how a neural signature of ‘learned salience’ and/or attentional gain for the category-diagnostic information might align with overt attention directed toward distinct stimuli in the same frequency bands.

We observe that category learning drives prioritization or gain across a category-relevant dimension. This is reflected in greater cortical activation in voxels whose frequency response is associated with the category-diagnostic frequency band, compared to the simultaneously presented non-diagnostic band. This prioritization persists even when the in-scanner task shifts to categorization based on a completely different auditory dimension, amplitude. Further, it partially aligns with the patterns of cortical activation that emerge when listeners explicitly direct sustained attention to unrelated sounds situated in the same frequency band. This is consistent with attentional gain accounts of advantaged perceptual processing of category-diagnostic dimensions. These neuroimaging results support the notion that learning complex categories can drive the emergence of acquired attentional gain implicitly directed to category-relevant perceptual dimensions.

The question of whether - let alone how - sensory cortical representations are impacted by the acquisition of category expertise has not had a clear answer. Evidence of cortical modulation with category expertise comes predominantly from visual cortex under conditions of active categorization of static images [14,56]. However, these results are mixed, with some studies not observing effects of categorization on visual cortex [57–60] potentially consistent with category representations arising in flexible, more ‘amodal’ cortical areas with little lasting influence on sensory cortex ([57,61,62], but cf. Hausfeld et al. [63] and Henderson et al. [12]). Jiang et al. [64] report auditory category learning results consistent with this two-stage model, and others have made a case for prefrontal involvement in auditory categorization [65]. In contrast, our results indicate stable, training-induced changes in auditory cortex that persist even when task demands direct attention away from the category-diagnostic dimension (see also Folstein et al. [66], and Van Culick and Cauthier [16] for evidence from repetition suppression across visual categories).

These changes were driven by the specific demands of learning, and not by mere exposure to statistically structured sounds. Participants who trained to use frequency-band-diagnostic information to categorize alien identity exhibited neural selectivity consistent with acquired attentional gain to the diagnostic frequency bands. But those who learned the amplitude-signaled size categories across the very same sounds, thus experiencing the identity-diagnostic spectral information without learning to use it to categorize, did not. One caveat to this conclusion is that *Alien Identity+Size*-trained participants had overall much more exposure to alien sounds than *Alien Size*-trained participants, which could potentially drive differences. However, the fact that neural selectivity differed with learning trajectory and ultimate behavioral expertise within the *Alien Identity+Size*-trained group would argue against this explanation.

Although humans and other organisms exhibit an ability to learn across passive exposure to statistically structured input [67] [68], studies have observed that complex acoustic exemplars that model the complexity of speech are not well learned over passive exposure [69,70]. Yet alignment of these statistically structured sounds with active behavior – even incidentally and without overt feedback – can rescue category learning [35,71,72]. The cross-group differences in the present study demonstrates that the alignment of feedback with a diagnostic dimension played a role in eliciting the cortical selectivity toward those dimensions. These patterns of acquired attentional gain in human auditory cortex connect with nonhuman animal electrophysiological studies that observe that the task-relevance of an acoustic dimension drives changes in receptive fields consistent with attentional gain [73,74].

In this vein, Yin and colleagues [75] present evidence for top-down modulation of auditory cortex by frontal cortex in ferrets, along with induction of intrinsic auditory cortical patterns related to acquired categories that persist even in passive listening. Though we limited our investigation specifically to tonotopically mappable temporal cortex, the regions we observe to be modulated by category learning receive rich top-down feedback inputs, and exhibit modulation by behavioral context that extends beyond basic representation of perceptual dimensions (e.g., Zhong et al. [76]). Such top-down modulation has been well-documented in nonhuman animal electrophysiology (particularly in the visual system, e.g., Freeman et al. [57]; DeCutis and D’Esposito [77]) and may interact with the persistent changes to auditory cortical representation arising from category learning that we observe. However, as Addleman and Jiang [78] note, the neural mechanisms underlying shifts of experience-driven attention may or may not be supported by the same frontoparietal regions as goal-driven attention. The lack of reliably definable tonotopic maps in frontal and other non-auditory regions using 3T fMRI makes it challenging for the present study to adjudicate this question. However, very recent human intracerebral stereoelectroencephalography (sEEG) reports prefrontal and inferior frontal spectrotemporal selectivity at multiple electrode sites [79] suggesting that ultra-high-resolution fMRI using the present approach may yield fruitful insights.

In the present study, we see that participants - above all the most expert - show selectively greater activation for the category-diagnostic frequency band. Importantly, this effect is widespread across bilateral auditory cortex, particularly in anterior and lateral portions. This degree of auditory cortical modulation by the category-diagnostic dimension - even when task-irrelevant - is striking by comparison with other studies of sensory cortical category sensitivity which tend to show greater category readout in frontal regions (e.g., Jiang et al. [59,64]). In situating category-diagnostic dimensions into discrete frequency bands, our approach may have afforded greater sensitivity by aligning learning-driven cortical changes with the primary representational axis of auditory cortex.

The question of whether such category-learning-related modulation of neural activity is a consequence of learned attentional gain to diagnostic dimensions also has surprisingly little direct support, despite the centrality of this proposition in theories of visual [80–82] and speech [83] categorization. It is clear that explicit attention to stimulus features modulates representations in both visual [84–89] and auditory (Da Costa et al. 2013; [43,45,63,90]) cortical response. Here, we asked whether patterns of cortical gain driven by explicit attention are concordant with patterns that emerge implicitly across category-diagnostic dimensions in categorization.

Consistent with our prior research [43], sustained auditory selective attention across sequences of tones presented simultaneously in two frequency bands results in greater activation across the attended frequency band. Here, in the same listeners, these patterns aligned with the prioritization of frequency-selective cortical activity driven by categorization demands, but primarily in more lateral auditory cortical regions. Of note, this concordance is not driven by task demands; prioritization of the diagnostic frequency band persists even when task demands change and frequency band is no longer task-relevant. This suggests that prioritization of diagnostic information has become a stable, lasting part of how category exemplars are represented in experts that is absent in category-naïve listeners. To our knowledge, this presents the first empirical evidence linking acquired gain to category-diagnostic dimensions of acoustic input and overt, directed selective attention.

We used a novel soundscape that participants would not have previously encountered to minimize interactions with existing auditory representations. This soundscape was designed to model the core learning demands of natural auditory categories, such as speech: listeners must identify the dimensions most diagnostic of category identity and learn their acoustically variable patterns amid competing dimensions, amplitude fluctuations, and background noise. We find that expert listeners who have successfully met these demands display cortical response signatures consistent with implicit, automatic attentional gain for category-diagnostic dimensions. This provides a mechanistic basis for a foundational, yet previously untested, theoretical tenet of speech categorization [83], with implications for understanding and potentially remediating learning-related communication disorders like dyslexia that have been linked to auditory learning [91] and auditory category learning [92,93] challenges. Furthermore, our finding that the specific learning trajectories by which listeners achieve categorization expertise leave a lasting imprint on cortical activation patterns suggests a neural basis for individual differences in speech categorization that persist even in neurotypical adults [94].

We should emphasize that auditory cortical fMRI activation differences driven by categorizing alien identity are decidedly not identical to those evoked by overt attention to pure tones with frequencies in the higher or lower category-diagnostic spectral bands. Although tonotopy concordance with alien identity and attention tonotopy activation is overall quite similar (with both showing particularly strong concordance in left anterior ROIs, and to a lesser extent along the right STG), direct concordance between alien identity and attention tonotopy high-low frequency difference maps is greatest along bilateral STG, and not the anterior-most left ROIs. While our previous work [43] did show particularly consistent attentional tonotopic modulation along many of these STG regions, it is unclear what is driving this left anterior difference.

One limitation for interpretation lies in attention tonotopy task performance; the attention task was difficult, particularly for some participants, and lapses in sustained attention to one or both frequency bands by a subset of participants could influence the robustness of results. (The task was also run at the end of the long fMRI session, so some participants were somewhat fatigued). Follow-up studies would benefit from a longer training regime for the attention task, similar to that for the alien identity task, to assure consistent performance across runs. A further limitation is that the ROIs were created and morphed on a hemisphere-specific basis, thereby precluding quantitative cross-hemisphere comparisons of qualitative differences, such as the qualitatively higher concordance between attention tonotopy and alien identity tasks in the right compared to left STG.

More globally, the current results shed light on what processes might be leading to expertise-driven changes in neural recruitment [22,35,95–97]. Often, experts show selectively increased sensory cortical activation for the object of expertise (e.g., [98,99]), but the causal change in neural or cognitive processing leading to this activation can be challenging to ascertain [100,101]. Here we show that selective activation changes in experts are systematically related to the locus of category-disambiguating information, even when that information is not immediately task-relevant. This, along with the relative similarity of category- and attention-selective frequency modulation maps (Fig 8), lends support to accounts suggesting learned expertise drives a lasting attentional bias for category-relevant dimensions (Folstein et al. [102]). Alternative accounts emphasize dimensional stretching (Folstein et al. [102], or attention-gated plasticity Sasaki et al. [62]). It is important to note that extensive experience with perceptual categories or objects, such as one’s own name or ringtone, can promote automatized overall attentional capture (e.g., [103]). However, the neural signature of such global ‘attention-grabbing’ effects will differ from those evoked by dimension-selective attention when the category-diagnostic perceptual dimension is topographically mapped in cortex (for instance across retinotopic space, or acoustic frequency as in the present study), and the presence of category-informative information may be differentially distributed within subregions of this space [99,104].

Disambiguation of different accounts could be furthered through explicit modelling of neural response functions that would efficiently partition category exemplars (e.g., Liu et al. [105]). Such modelling may help to shed light on why there is greater category-selective modulation of activation in anterior, versus posterior, auditory cortical regions, particularly in the most expert listeners. Additionally, data that disambiguate signals from upper versus lower cortical layers would potentially reveal more fine-grained information about neural encoding of category-relevant information [106] as well the role of learned attention in such encoding [107,108].

In future work, it will be important to show that the increased activation to identity-category-relevant frequency regions that is evident when they are not task-relevant (e.g., during size judgments) is not resulting from carry-over across experimental blocks. While we believe this is unlikely given the difficulty of the amplitude-conveyed alien size task (where weighting by spectrotemporal cues is counterproductive), it is possible that the activation patterns indicating acquired gain of the identity-diagnostic frequency band may diminish when not regularly reinforced via interleaving with blocks of the identity task.

Use of a more naturalistic or captivating training paradigm [71,109,110] would promote extended training and consolidation, which would be particularly revealing in tracking slower learners like those who did not achieve full mastery in the present study. Greater training time, along with a larger sample for fuller examination of individual differences, would allow for better understanding of potential non-linearities in the relationship between learning and changes in category-related neural sensitivity. As well, since the present study establishes expertise-driven attentional gain to diagnostic dimensions, future studies could observe the emergence of this attentional gain as participants learn categories during scanning. This will reveal how category learning shapes neural activation on a minute-by-minute or session-by-session basis (in the style of Lim et al. [71] and Ittipuripat et al. [111]). We also predict increasing learned salience such that identity category experts would exhibit further increases in gain for the identity-diagnostic band in the size categorization task, despite the task-irrelevant nature of frequency-band-delimited alien identity information in this task (see also Wang et al. [55]).

In all, learning categories results in prioritization of category-relevant perceptual information across a representational dimension reflected in systematic changes in neural response to complex sounds. This category-selective difference in activation persists even when the dimension is irrelevant to the task at hand and aligns closely with patterns of activation that emerge with explicit, sustained attention to the dimension across unrelated sounds. Thus, acquiring new categories can drive the emergence of acquired attentional salience to category-diagnostic dimensions.

## Methods

### Ethics statement

This study was approved by the Institutional Review Board of Carnegie Mellon University (approval STUDY2019_00000322), and by the University College London (UCL) Psychological And Language Sciences Ethics Committee (approval number fMRI_2022_002). All participants provided written consent. The study has been conducted according to the principles expressed in the Declaration of Helsinki.

### Participants

Our study involved two groups. The *Alien Identity+Size* group trained to categorize sounds according to alien identity (based on complex spectrotemporal patterns) and, separately, to categorize the same exemplars according to alien size (based on amplitude). An *Alien Size* group trained only on alien size. All participants were fluent English speakers with normal hearing, had normal or corrected-to-normal vision and no history of neurological impairment or speech/language disorder, and reported no experience with a tonal language.

Fig 1A shows the participant groups. The *Alien Identity+Size* group included participants from the Pittsburgh, PA, USA and London, UK communities who completed a five-day training protocol (N = 95; 18–40 yrs). Individuals who met criterion levels of expertise in alien identity categorization (75% across both diagnostic frequency bands on the 2AFC alien identity task on Day 5) were invited to participate in a subsequent MRI session, with data collected from N = 54 experts. Five participants’ data were excluded (motion, non-compliance with task) resulting a final sample of N = 49 experts (18–37 yrs, M = 23.9 yrs; 29 female, 18 male, 2 non-binary; 59% White; 32 Pittsburgh, 17 London).

The *Alien Size* group (N = 20, 18–43 yrs, M = 28.1 yrs; 12 female, 8 male; 55% White; 20 London) trained only on the alien size task and participated in an abbreviated MRI session.

### Stimuli

Four auditory categories were sampled from a highly complex perceptual space (see Obasih et al. [40]). As shown in Fig 2, each stimulus was composed of patterned acoustic energy in two non-overlapping acoustic frequency bands (~80–750 Hz and ~1,000–9,000 Hz). Each band was populated with three 400-ms tonal ‘chirps’ (100-ms ISI) varying in frequency contour. In the *alien-identity-diagnostic* frequency band (high-frequency for Categories A, B and low-frequency for Categories C, D), the three acoustically variable chirps possessed an underlying regularity that defined alien identity category membership. The three temporally aligned chirps in the non-diagnostic frequency band (low for Categories A, B; high for Categories C, D) were also acoustically variable but possessed no coherent underlying regularity.

To create a large set of naturally varying category exemplars, we derived chirps from four native Mandarin Chinese talkers (two female) uttering single-syllable utterances in each of Mandarin’s four lexical tones, which convey variable, but structured, fundamental frequency (F0) contours. We extracted the F0 contour from each and resynthesized the contour to create a non-speech chirp, time-normalizing each to be 400 ms. We pitch-shifted each chirp +33 semitones, then applied a 1,000 Hz high-pass filter to create a pool of chirps to populate the high frequency band. Equivalently, to create chirps for the low frequency band, we pitch-shifted the F0 contour −1 semitone and applied a 500-Hz low-pass filter.

Acoustic information in the category-diagnostic band consisted of three distinct chirps derived from the same talker and the same Mandarin lexical tone category, thereby conveying an acoustically variable but structured regularity in frequency contour. Acoustic information in the non-diagnostic band consisted of three different chirps drawn from three *different* Mandarin lexical tone categories (from among any of the four) of the same talker such that there was no underlying acoustic regularity across chirps. In all, this created a stimulus pool of 36,000 novel category exemplars [40] from which we sampled 592 unique stimuli.

In addition, we also imposed orthogonal amplitude variation (between 62−80 dB) on copies of each of these 592 sounds. We manipulated amplitude on an exemplar-wise basis after the two spectral bands were combined, so amplitude manipulations scaled equally over the entire spectrum. This met two goals: to increase variability and task difficulty, and to permit creation of two *Alien-Size* categories*,* with ‘Big Alien’ exemplars falling between 72−80 dB sound intensity, and ‘Small Alien’ exemplars falling between 62−70 dB (all reported dB referenced to 2*10^−5^ and measured in Praat). Thus, each exemplar could be categorized along identity (spectrotemporal, frequency-band-specific) or size (amplitude) dimensions. Not only were stimuli acoustically identical across Big and Small alien size categories aside from amplitude, they were also identical across the Size and Identity tasks, with the only difference being the task that participants performed on the stimuli.

Collectively, these stimulus features impose several key demands on learning: (1) Because chirp triplets appear in two distinct frequency regions, but statistically structured patterns diagnostic of alien identity occur in only one, listeners must first discover which frequency band carries meaningful regularities; (2) Because two distinct alien identities are conveyed by different patterns within the statistically structured frequency band, listeners must learn how specific regularities map onto identity. Beyond frequency-based demands, identity-category learning requires listeners to generalize across identity-irrelevant amplitude variation, whereas size-category learning requires them to rely on amplitude as the relevant cue. Identity and size category training take place against a background of scanner noise, modelling learning under adverse listening conditions.

### Procedure

Online categorization training was implemented using Gorilla software (Anwyl-Irvine et al. [112]), the Google Chrome browser, wired headphones (with headphone compliance screened as in Milne et al. [113] and wired headphone usage reinforced in recruitment instructions), and a computer/laptop (not tablet/phone). Stimuli were created with Matlab version 2021b (The MathWorks), Praat [50] and SoX version 14.4.2 (www.sourceforge.net).

**Behavioral Training Prior to fMRI.** Fig 1B-1C illustrate the approach to training. *Alien Identity+Size* participants learned to associate 1,400 ms novel sounds with one of four alien images via category-informative feedback identifying the correct alien after each response. Each of 5 daily sessions involved four blocks, each composed of 120 alien identity training trials followed 20 trials to test generalization of learning (Fig 1C). Alien identity categorization training involved 2AFC trials in which participants indicated alien identity with a keypress, and feedback (1,500 ms) indicated the correct alien. In each 120-trial training block, trials were segregated according to the category-diagnostic high or low frequency band (Categories A versus B and Categories C versus D). Every 20 trials, the category pair, and thus diagnostic frequency band, alternated; the other two alien-identity response options were blanked out as response alternatives (Fig 1C), with order counterbalanced across participants. Training stimuli were randomly selected from a pool of 512 exemplars (128/category). Amplitude varied from 62–80 dB across trials and, over the entirety of training, was balanced across this range.

Each 2AFC alien identity training block was followed by a 20-trial 4AFC generalization block with all aliens as response options, and no feedback (Fig 1C). These trials involved a separate pool of 20 exemplars/category that were never presented in training to assess generalization of learning (Fig 1B-1C). Across four 2AFC training and 4AFC generalization blocks, category exemplars were mixed with a fixed level of scanner noise (57 dB) to familiarize participants with categorization in an MRI environment. The visual position of aliens (and associated keypress) was fixed during the first two days of training, then allowed to vary starting on Day 3. (Note that Obasih et al. [40] found that alien category learning speed and attainment were surprisingly unaffected by different training regimes, including manipulations of exemplar variability, blocking versus interleaving categories in training, and overt instructions to attend to the diagnostic band.)

On Day 1 and Day 5, these participants also trained on alien size categorization (Fig 1B-1C). Across the first eight trials, one exemplar from each of the four categories was presented at the highest amplitude (80 dB), then one exemplar from each alien category was presented at the lowest amplitude (62 dB). The amplitude difference diminished in regularly incremented dB steps every 4 trials for a total of 40 trials, with 71 dB defining the category boundary between ‘big’ and ‘small’ aliens. In a second 40-trial block, amplitude values were fully randomized with equiprobable big/small exemplars and an equal number of exemplars from each alien category. A fixed level of scanner noise (again 57 dB) was mixed with stimuli to mimic the MRI sound environment. Additionally, a separate *Alien Size* participant group completed a single session of 2AFC alien size category training over 80 trials (Fig 1B).

**Tonotopy.** Participants reported infrequent mini-sequence repeats in a 1-back task to characterize voxel-wise frequency selectivity (Fig 1C). Individuals were scanned as they listened to sequences of 178-ms sinewave tones (0 ms ISI, 5 ms ramp at on/offset), organized in 4-tone mini-sequences, each separated by 356 ms. The frequency range of the four tones composing the mini-sequences increased or decreased in frequency incrementally in 10 logarithmically scaled steps to sweep across 175–5286 Hz (60 semitones), each with 6 mini-sequences. Across the 60 mini-sequences comprising each 64-sec sweep, 3 mini-sequence repeats occurred quasi-randomly. Participants reported repeats with a button press. The swept frequency permitted fMRI analysis using a) phase-encoded mapping, where voxels responsive to a particular frequency should respond at a consistent phase delay [43] (Dick et al. [114]; Sereno et al. [115]) and b) multiple regression analysis to identify voxels responsive to the range of the lower (< 500 Hz, lowest three steps) and higher (≥1000 Hz, highest five steps) identity-category-diagnostic frequency bands.

For analysis of training and in-scanner behavior, the response window for each mini-sequence was defined from the onset of the final tone to the onset of the final tone in the subsequent sequence (1068-ms response window). For calculation of d-prime scores, extreme hit and false alarm rates of 0 or 1 were adjusted following the approach of Stanislaw and Todorov [116]. As an introduction to the task, participants were familiarized with one up-sweep and one down-sweep on Day 4 of online training (or at the end of the single training session for the *Alien Size* group), with mini-sequence repeats indicated on-screen (Fig 1B). As with category training, scanner EPI noise (57 dB) was mixed in with the tone stimuli (~80 dB) used to map tonotopy.

**Attention tonotopy.** Participants who trained on the *Alien Identity+Size* categories completed a task to characterize voxelwise frequency-selective and amplitude-selective overt attentional responses in individual participants. Following the approach of Dick and colleagues [43], participants heard 4-tone mini-sequences evolving simultaneously in two frequency bands with instructions to “attend low” or “attend high” and report mini-sequence repeats from the attended frequency band (Fig 1C). To parallel the amplitude differences signaling alien size categories, mini-sequences alternated between higher and lower amplitude levels, which differed on average by ~10dB. A different spoken instruction directed participants to “attend volume,” reporting 1-back matches on the attended amplitude dimension; data from the "attend volume" condition were not used for the present study. Mini-sequences were composed of 180-ms sinewave tones drawn from a pool of four frequencies (1, 3, 5 and 7 semitones above a ‘base’ frequency). Tones were drawn with replacement, with the constraint that a sequence could never be a single tone played four times. Simultaneous tones from high and low frequency bands were temporally aligned. Mini-sequences were separated by 360 ms silence in short blocks that concluded with 2 sec of silence. Each block included two mini-sequence repeats in the high-frequency band, two repeats in low-frequency band, and two amplitude repeats. The 1080-ms response window for each mini-sequence was defined from the onset of the final tone to the onset of the final tone in the subsequent sequence.

Participants were familiarized with the task on Day 3 of categorization training and first experienced mini-sequences in single frequency bands of fixed amplitude (Fig 1B). Next, amplitude variability was introduced (first only with the task of reporting tone sequence repeats), then finally, participants practiced with dual-frequency-band amplitude-variable stimuli. On-screen messages alerted participants to mini-sequence repeats in the attended band. There was no scanner noise mixed with stimuli during familiarization. Participants then completed 24 attention tonotopy trials (3 blocks/trial, 11–17 mini-sequences/block), with on-screen feedback provided for the first half of the trials. For half of the trials, the dual-band stimuli had base frequencies of 280 and 1,253 Hz, and for the other half, the base frequencies were 400 and 2,123 Hz. “Attend high,” “attend low” and “attend volume” trials were equiprobable. The amplitude of the stimuli was set so that the two frequency bands were approximately equated on perceptual loudness (~73 dB average), and a fixed level of scanner noise (57 dB) was presented throughout these trials.

**MRI procedure.** Images were acquired in a single 90-min session with 3T Siemens Prisma scanners and 32-channel head coils (at the CMU-Pitt BRIDGE Center in Pittsburgh, RRID:SCR_023356 and at the Birkbeck-UCL Centre for Neuroimaging in London). Identical pulse sequences were used across sites, and very similar QA procedures were in place by the respective MRI center teams to monitor scanner performance. Head movement among London participants was minimized with a head stabilization prototype (MR Minimal Motion System). Both sites delivered audio over Sensimetrics earbuds with pre-filtering to accommodate the earbuds’ response profile, presented tasks using PsychoPy (v. 2022.2.4), and recorded responses with a button box.

Participants in the *Alien Identity+Size* group who qualified for the MRI session completed a 15-min online refresher one day prior to the scan. This involved the tonotopy task (one 64-sec up-sweep, one 64-sec down-sweep), 80 2AFC categorization trials (48 alien identity, 32 alien size, drawn from the pool of 80 generalization exemplars with no feedback), and two short 9-block attention-tonotopy trials with parameters mirroring the MRI task. Participants in the *Alien Size* group completed a refresher within one day of their scan. It included the tonotopy task (one 64-sec up-sweep, one 64-sec down-sweep) and 32 trials of 2AFC alien size categorization, with no feedback.

At the scanning session, structural images were acquired using a T1-weighted magnetization-prepared rapid acquisition gradient echo (MPRAGE) sequence (TR = 2,300 ms, TE = 2.98 ms, FOV = 256 mm, flip angle = 9°) with 1 mm sagittal slices. Functional echo planar images were acquired using a CMRR [117] T2*-weighted sequence (TR = 1.0 s, TE = 30 ms, 44 slices, 2.0 mm thickness, in-plane resolution = 2 mm × 2 mm with 6/8 partial Fourier encoding, FOV = 212 mm, flip angle = 62°, multiband acceleration factor = 4); we included eight dummy volumes at the start of each scan to allow the scanner to reach B1 equilibrium. Functional images were acquired with anterior-to-posterior phase encoding; following each task, we collected two additional volumes with reversed phase encoding to correct for image distortion caused by B0 inhomogeneities.

The scanning session for *Alien Identity+Size* participants started with MPRAGE acquisition. Next, participants completed four runs of the tonotopy task (4 sweeps/run; 1st and 3rd up-sweep, 2nd and 4th down-sweep) during which they fixated on a central cross overlaid on a small landscape image to minimize eye movements associated with the acoustic sweep; images changed over the course each run (5 images/run; random duration to avoid aliasing with the acoustic sweep rate).

Next, for *Alien Identity+Size* participants, there were six runs (10 blocks each, 8 trials/block) of 2AFC categorization. Across runs, 20 blocks involved categorization of alien identity across the high diagnostic frequency band, 20 involved identity categorization across the low frequency band, and 20 involved size categorization across amplitude. Use of the 2AFC (rather than 4AFC) categorization task in the scanner afforded several benefits: (a) increasing power to differentiate across higher and lower frequency bands by minimizing switches within a block; (b) reducing saccades across distinct visual aliens; and (c) constraining each block to two categories making identity and size tasks completely parallel. Our prior behavioral study demonstrated no learning advantage for even explicit instruction indicating the diagnostic frequency band [40]. A pseudorandomized block order common across participants assured that blocks reliant on the same diagnostic dimension did not occur consecutively. Overall block order in runs 1–3 repeated in runs 4–6, but with trial order shuffled, and with the amplitude set to a different level within the ‘big’ or ‘small’ amplitude range. In all, there were 320 alien identity categorization trials (80/category, with equal exemplars from the two size categories) and 160 alien size categorization trials (with equal exemplars from the four identity categories). Note that the 2AFC categorization fMRI stimuli were drawn from the pool of 80 generalization exemplars encountered in training. Prior to the fMRI experiment, participants had received no feedback with these stimuli, and never made a 2AFC decision with them. Each of the exemplars was encountered at two different amplitudes.

To facilitate comparisons between the alien identity and alien size categorization blocks, 20 of the 40 blocks that appeared during the 2AFC alien identity task also appeared in the 2AFC alien size task, with the same fixed trial order within the block. By design, the exemplars (and their order) in the identity and size task blocks were identical to avoid potential stimulus, context, and order confounds. Half of the alien size blocks appeared before their corresponding alien identity blocks, and half appeared after. Each block began with a 3-sec visual display of the task (Alien Identity or Alien Size) followed by 1.5 sec for the audio and a 1.5 sec response window.

Finally, there were two runs of the attention-tonotopy task (one with base frequencies of 280 and 1,253 Hz, one with base frequencies of 400 and 2,123 Hz). Each run involved 15 blocks (22–34 sequence/block), with 12–15 sec of rest after every 3 blocks (except after the final block in a run). Auditory instructions at the beginning of each block indicated the locus of attention (“high,” “low,” “volume,” “rest”). As in the tonotopy task, participants fixated on a central cross overlaid on changing landscape images to avoid high- or low-band-attention-related eye movement (5 images/run).

The *Alien Size* participant group completed an abbreviated MRI protocol that included structural imaging, four runs of the tonotopy task, and two runs of 2AFC size categorization. Critically, the 2AFC categorization task trials followed the same presentation of alien size categorization trials (in the same order) as the *Alien Identity+Size* group.

### Analyses

**MRI analyses.** Cortical surfaces were reconstructed for each participant from the T1-weighted MPRAGE using FreeSurfer (Dale and Sereno, [118]; Dale et al. 1999; Fischl, [119]).

Functional images from the tonotopy task were minimally preprocessed using AFNI (Cox [120]) to account for saturation by discarding the first 8 volumes, to unwarp images using phase-reversed images collected at the end of each task, and to align all volumes to a reference volume from the middle of the first run. Next, the reference volume was aligned to the cortical surface in Freesurfer using boundary-based registration (Greve and Fischl [121]).

To establish the regions of auditory cortex showing frequency selectivity within and across subjects, we calculated tonotopic maps using Fourier-based analyses on the preprocessed functional data from the tonotopy task in csurf (https://pages.ucsd.edu/~msereno/csurf), following standard phase-encoded mapping approaches [43] (Sereno et al. 1995; Dick et al. 2012). Relying on the fact that each frequency step occurs at a consistent time within a sweep, Fourier analysis allowed us to compute a set of F-statistics indicating how each voxel responds at the frequency of stimulus cycling (4 cycles per run, 4 64-sec sweeps per run) relative to other frequencies; the phase lag (i.e., the delay relative to the start of the cycle) of the maximal response can be used to determine the frequency preference of each voxel. We performed Fourier analysis of each run (up-sweeps time-reversed for phase averaging with down-sweeps).

Finally, we painted the phase of the signal (indicating frequency preference) as a color map and visualized the data on each participant’s cortical surface reconstruction. Projecting each subject’s data onto an icosahedral surface and averaging across participants created a group map that was projected to a single participant’s surface reconstruction for visualization. Subsequent region-of-interest (ROI) analyses, described below, were limited to regions in auditory cortex where tonotopic organization was observed at the group level in participants expert in alien-identity categorization.

We next performed a regression-based analysis on the preprocessed functional data from each of the three tasks using 3dREMLfit in AFNI (Chen et al. [122]). For each analysis, we constructed regressors by convolving a vector of the onset times for each frequency step with a square wave of the appropriate duration. Rigid-body motion parameters (movement in the x-, y- and z-axis directions, as well as pitch, roll, and yaw) were included as regressors of no interest. The resultant beta estimates were used to compute, for each task, the difference in activation observed according to higher versus lower frequency band.

In the categorization tasks, this corresponded to the difference in blocks involving categorization of alien identity across the higher frequency diagnostic band versus those involving the lower frequency diagnostic band. In the attention tonotopy task, this corresponded to the difference between explicitly attending to high- versus low-frequency mini-sequences. Results were mapped to the cortical surface using the mri_vol2surf Freesurfer command. To generate group-level contrast maps, subject-level maps were projected onto an icosahedral mesh and averaged, the result of which was displayed on a single subject’s surface.

Although there is a macroscopic cross-participant similarity in large-scale tonotopic gradients, there is considerable cross-participant variability in frequency preference at a finer grain, particularly when voxels are aligned by cortical folding patterns [41,123]. Thus, to measure regional encoding and cross-task concordance in frequency selectivity while taking this variability into account, we calculated the degree of concordance in cross-task voxel-wise frequency selectivity in a set of small cortical surface ROIs defined in a previous study (Dick et al. [43], see Fig 4). Here, we limit our analysis to those ROIs where tonotopic selectivity was observed at the group level in our alien-identity categorization experts. For each participant, the ROIs that tessellate tonotopically organized auditory cortex (37 per hemisphere) were projected from a single participant to the unit icosahedron (sphere_reg), then projected again to each curvature-aligned participant’s surface, and finally to the bbregister-aligned native space 2 x 2 x 2 mm EPI space using Freesurfer’s mri_label2vol.

For each ROI, cross-task concordance was evaluated via regression analysis, where all subject-wise beta coefficients in one task (e.g., *b*_tonotopy(high-low)_) were used to predict the high-low betas in another task (e.g., *b*_attention-tonotopy(high-low)_); each regression model included random intercepts for each participant. In other words, there is a regression model fit for each ROI, where for each participant, the number of betas per task equals the number of voxels in the native-space projection of that ROI. The voxelwise beta coefficients for the tonotopy task are from the general linear test (GLT) of high minus low frequency bands; correspondingly, the coefficients for the alien identity task are from the GLT of high minus low category diagnostic bands. Betas are always paired voxelwise for all cross-task concordance maps.

The resultant *t* values from the mixed effect models were transformed to *z* statistics, and these *z* values were painted onto the cortical surface. Significance (alpha < 0.05) was assessed using one-tailed tests, as we had *a priori* hypothesized a positive relationship between betas across tasks. To control for multiple comparisons, we applied a false discovery rate (FDR) correction on our *p* values using the 3dFDR command in AFNI. Note that because 3dFDR performs a two-tailed adjustment, *p* values associated with negative *z* statistics were inverted (i.e., 1-p) both prior to and following FDR correction, yielding FDR-corrected values that reflect one-tailed tests. Similar analyses were performed for different subgroups of participants defined based on behavioral performance during training.

We also conducted a series of analyses to compare cortical responses between subgroups of *Alien Identity+Size* participants (e.g., in participants who acquired category expertise early in training versus those who acquired expertise over the course of training; see Fig 6) as well as compared to *Alien Size* participants. These regression analyses were akin to the ones described above but also included a main effect of Group, as well as its interaction with Task; this interaction term allows us to assess whether cross-task concordance differed as a function of group. The associated *t* values from the interaction term were converted to *z* values and projected onto a single subject’s cortical surface, providing an index of which group demonstrated stronger cross-task concordance. As with other analyses, significance was assessed using one-tailed tests and FDR corrections were applied to control for multiple comparisons.

## Supporting information

S1 Fig Cortical-surface-based group averages of phase-encoded tonotopic maps from the alien identity + size training group (top) and alien size training group (bottom). Color mapping shows phase of maximal fMRI response at 4 cycles/run; this indexes preferred auditory frequency via the high-to-low or low-to-high frequency sweep that repeats 4 times during each tonotopy run. (See Dick et al. 2012 and **Methods**-**Procedure** for details).
(TIFF)

S2 Fig Differences in Concordance Maps for *Alien Identity + Size* Trained Group (N = 49) compared to *Alien Size* Trained Group (N = 20). S2 Fig shows the difference between the tonotopy and alien-size-task concordance map for all *Alien Identity+Size* trained experts (shown in bottom panel of Fig 5B) versus that for the *Alien Size* trained participants (shown in Fig 7, panel A). The summary data used for this figure are available at https://doi.org/10.5522/04/33974425.
(TIFF)

## Abbreviations

2AFC: two-alternative forced choice
FDR: false discovery rate
4AFC: four-alternative forced-choice
fMRI: functional magnetic resonance imaging
HG: Heschl’s Gyrus
LH: left hemisphere
ROIs: regions of interest
RH: right hemisphere
sEEG: stereoelectroencephalography
STG: Superior Temporal Gyrus
STS: Superior Temporal Sulcus
UCL: University College London
