# Supplementary material for: Category learning drives cortical attentional gain to diagnostic dimensions of perceptual information
Source: PLoS Biol. 2026 Nov 12;24(11):e3004076. doi: 10.1371/journal.pbio.3004076 (PMC13759068; doi:10.1371/journal.pbio.3004076)
Supplement: Supplementary file 1 [file pbio.3004076.s005.pdf]

Dear Dr. Schnell,

Thanks to you and the four reviewers for the time and thought on our manuscript (PBIOLOGY-D-25-04029R, *Category learning drives cortical attentional gain to diagnostic dimensions*). The critiques have been very useful in improving the paper scientifically, and in helping us to see where we have not clearly explained key aspects of this rather complicated study. We hope that we have made good use of everyone's input and have made the paper both more convincing in its presentation of findings, and a more straightforward read.

Below, we have copied in each of Reviewer 1-4's (**R1**, etc.) points verbatim, numbering them for clarity (e.g., **R1.1, R1.2**). Author responses (***AR***) are in *italics* and are numbered accordingly (***AR-R1.1**,* etc.).

Responses to Reviewer 1 (R1)

R1.0 (Chris Petkov signed his report): Below, I follow a structured peer-review format in the layout of my review report. As you'll see, I have more to say about study strengths than what could be improved to strengthen the manuscript (only a handful of relatively minor suggestions).

Summary: This is a tour-de-force study studying the interface of category learning and attentional gain in the auditory system. The authors conduct a well powered (n = 69) an category learning study with 5 days of training prior to fMRI. Two different groups of participants take part in either the alien identity and size task or just the size task; size of the alien selected on the screen is dependent on the amplitude of the sounds heard, and identity is related to a combination of frequency and spectro-temporal features that the participant extracts from a large set of exemplars. After five days of training the participants are scanned with fMRI at 3T and the results contrasting the category (identity and/or size) information with concordance maps determined from tonotopic mapping individually but transformed to a common space and attention to tonotopy task effects. The results demonstrate that category learning harnesses mechanisms more commonly attributed to selective attention effects, and that even though there are regularities in the sounds, the effects related to attention are strongest when the category information is task relevant. The results have strong implications for understanding expert learning, and may be relevant for developing further to diagnose learning related difficulties in combination with behavior and neuroimaging, although this could be better developed in the discussion.

Study strengths: There are several strengths of this study, including the large sample sizes and dedication to multi-day training prior to fMRI. The strength of the large sample sizes mean that the research team can do sub-group comparisons of identity and size with size learning groups as well as early versus late experts or high-low achievers. The benefit of the task being non-linguistic is that the sounds and aliens are not readily verbalizable to reduce language-related

confounds. The fMRI activity maps are registered to the subject's specific tonotopic map. The size task is not trivial and the authors have done a tremendous job matching task difficulty between the identity and size task performance for the scanning session, to rule out task difficulty as a potential confound. Moreover, the study is very well written and the narrative with figures and results elegantly relayed to the reader with few unaddressed questioned within the relevant sections.

AR-R1.0: *We are very grateful for Dr. Petkov's generous comments, and also for the helpful and concrete suggestions that have helped us improve the manuscript.*

R1.1: Auditory cortex labels and quantification would be important to see: To auditory cortex non-experts, it would be important to add labels to identify the subregions of auditory cortex. More importantly, it would be useful to quantify the effects attributed to STG, HG or other subregions of auditory cortex in the discussion.

AR-R1.1: *Thank you for pointing out the serious lack of anatomical labelling. In looking anew at the figures, we now see how challenging it is to see the underlying anatomy in all of the 'ROI quilt'-based figures, in particular. We have addressed this in a few different ways. First, we have included an insert in the first cortical surface figure (Fig 4) labelling the relevant sulci and gyri on the surface alone, as well as with the ROI outlins shown. Second, in Figures 5-8, we include a light tracing and labelling of Heschl's gyrus along with the superior temporal gyrus. These figures were already quite information-dense, so we have erred on the side of clarity and only put in a few guidepost labels that the reader can also use as reference points across figures.*

Additionally, the revised text now more clearly highlights that the ROI tiling doesn't precisely align with anatomical landmarks or areal boundaries. As a result, we have been circumspect when drawing conclusions about how these ROIs are related to structural boundaries. Each figure's colorscale shows the statistical values for each small ROI. Thus, in the Discussion, we describe the relative activation differences in the sets of ROIs that roughly correspond to the anatomical regions. If it's ok, we would opt to keep showing the quantitative effects in the figure at an ROI-wise level without doing any statistical tests on ROI anatomical groupings, since these would have to be post hoc. If R1 thinks it would be better to run those tests, or depict activation differences by region, we can definitely do that and just note in the manuscript that the analyses were suggested by a reviewer (as opposed to something we had initially hypothesized).

> *<u>Associated text from the Discussion</u>: "We should emphasize that auditory cortical fMRI activation differences driven by categorizing alien identity are decidedly not identical to those evoked by overt attention to pure tones with frequencies in the higher or lower category-diagnostic spectral bands. Although tonotopy concordance with alien identity and attention tonotopy activation is overall quite similar (with both showing particularly strong concordance in left anterior ROIs, and to a lesser extent along the right STG), direct*

concordance between alien identity and attention tonotopy high-low frequency difference maps is greatest along bilateral STG, and not the anterior-most left ROIs. While our previous work (Dick et al., 2017) did show particularly consistent attentional tonotopic modulation along many of these STG regions, it is unclear what is driving this left anterior difference. A limitation for interpretation lies in attention tonotopy task performance; the attention task was difficult, particularly for some participants, and lapses in sustained attention to one or both frequency bands by a subset of participants could influence the robustness of results. (The task was also run at the end of the follow-up studies would benefit from a longer training regime for the attention task, similar to that for the alien identity one, to assure consistent performance across runs."

R1.2: Brain wide effects underspecified: I understand the importance of focusing on effects over tonotopic cortex altered by category learning and influenced by selective attention, but it would be useful to have at least one main manuscript figure that shows the brain wide effects of identity and size category comparisons.

AR-R1.2: *We agree entirely and have included the activation maps for both conditions in Fig 5. Because of the lack of reliably definable tonotopic maps in frontal and other non-auditory regions using fMRI (especially with 2x2x2mm voxel sizes), we would not be able to measure attentional modulation that is toggled to the category-diagnostic frequency band. However, in the discussion, we point to very recent sEEG work by Hullett et al. (2025) showing prefrontal and inferior frontal spectrotemporal selectivity at multiple sEEG electrode sites, suggesting that follow-on studies focusing on these regions with ultra-high-resolution fMRI may be a fruitful continuation of this work. We are really excited about this possibility, but given it will require quite a different analytical approach, we will put in a promissory note for upcoming work, if that is acceptable to R1.*

<u>Associated text from the Discussion</u>: "The lack of reliably definable tonotopic maps in frontal and other non-auditory regions using 3T fMRI makes it challenging for the present study to adjudicate this question. However, very recent human intracerebral stereoelectroencephalography (sEEG) reports prefrontal and inferior frontal spectrotemporal selectivity at multiple electrode sites (Hullett, Leonard, Gorno-Tempini, Mandelli, & Chang, 2026) suggesting that ultra-high-resolution fMRI using the present approach may yield fruitful insights."

R1.3: Basis for categorization learning is unclear: I am pleasantly surprised that the high/low categorization was not as easy to learn as I would have thought (e.g., mentally draw a category boundary between the low and high frequency space - done deal). Therefore, it seems the identity categorization relied also on the spectro-temporal features within the high/low categories. It would be useful to at least comment in the discussion on how the category learning also relied on features within the high/low frequency space that were diagnostic for the alien identity.

AR-R1.3: *Thank you. This point, raised in other reviews as well, has helped us to recognize that we did not explain our stimuli sufficiently well.*

We addressed this in the revised manuscript with a new figure that schematizes the full task structure of the study (Fig 1, wisely suggested by R3).

We also emphasize the key features of our categorization task: (1) each acoustic stimulus is composed of three chirps situated in a high frequency band, and three simultaneous chirps situated in a low frequency band; (2) the chirps in one band are statistically structured because they are extracted from the pitch contour of three distinct utterances of a single Mandarin tone category; three simultaneous chirps in the other frequency band lack this statistical structure because they are extracted from three <u>*different*</u> *Mandarin tone categories; (3) the frequency band with statistical structure is category-diagnostic because it is associated (through feedback) with alien identity; chirps in the other, unstructured, band are irrelevant to alien identity; (4) in all, there are four alien identity categories – two with category-diagnostic patterns in the high frequency band and two with category-diagnostic patterns in the low frequency band; (5) across all sounds, the relative amplitude varies, but is uninformative to alien identity.*

These features impose several key demands on learning: (1) For each alien identity category, one frequency band is category-diagnostic and the other is irrelevant, so listeners must discover the spectral region that conveys category-diagnostic information in the context of highly similar acoustic input in non-diagnostic band; (2) Listeners must also learn the statistical regularity present across the chirps situated in the category-diagnostic frequency band because two distinct alien identity categories are conveyed by different regularities (derived from different Mandarin tones) in each band; (3) Due to amplitude variation, identity-category learning requires listeners to generalize across identity-irrelevant amplitude variation; (4) However, this amplitude variation is informative to size-category learning; (5) Notably, both identity and size category learning take place against a background of scanner noise, modelling learning under adverse listening conditions.

We now emphasize these points in the revised Methods, at the start of Results, and in the Discussion.

R1.4: I didn't think memorizing the alien identities would help since there were so many exemplars, but as you can tell I was a little confused on the basis for the alien identity learning besides the high/low frequency dimension that seems trivial to learn. Related to this, I wasn't clear how amplitude size was manipulated to ensure the task was not overly easy for participants (needing a little more clarity than I found in the methods).

AR-R1.4: *Again, we really did not make this clear enough by a long shot, as demonstrated by questions by multiple reviewers (and indeed our own re-reading of the descriptions). We've tried to rectify this in a few ways. First, we have added a new figure that schematizes the full task*

structure of the study (a new Fig 1, wisely suggested by R3). Second, we have revised the Methods to describe how stimulus amplitude varies in size category training, emphasizing that amplitude range varies incrementally (in dB) and that amplitude always varies across trials. Please also see the revisions to the Methods section for clarity, and the addition of text at the beginning of the Results section.

> *Associated text from the Results section: "These stimulus characteristics impose several key demands on learning: (1) because chirp triplets appear simultaneously in two distinct frequency bands but statistically structured patterns diagnostic of alien identity occur in only one band, listeners must discover the relevant regularity in the context of competing sounds; (2) chirp triplets within the category-diagnostic frequency band are acoustically variable yet possess an underlying spectrotemporal regularity that maps onto alien identity through feedback; (3) because this regularity differs across aliens, listeners must not only identify the diagnostic frequency band but also learn to map specific spectrotemporal patterns within each frequency band onto alien identity; (4) overall amplitude varies across sounds, such that learning alien identity requires generalization across identity-irrelevant amplitude variation; (5) learning takes place under adverse listening conditions, with sounds embedded in scanner noise throughout, including during behavioral training outside the scanner."*

R1.5: Broader impact discussion could be useful: The discussion is interesting in relating to the relevant literature, but I thought there was a missed opportunity to couch the observations in the utility of either the approach or the observations for diagnosing or ameliorating learning difficulties. Certainly there is interesting discussion on the non-attended category information not eliciting as strong of a modulation of auditory cortex as one might expect given statistical learning effects can be evident in the brain. But I wondered whether the current approach and results, such as those relating to statistical learning, could be further developed to understand auditory expertise (early or late learners) and those with language and/or learning difficulties, which of course are outside of the scope of this study but might benefit from some discussion with regards to the potential future impact of this work.

AR-R1.5: *We had frankly not considered this, which is a bit embarrassing given work in our labs on highly related topics - thank you for raising this point. We have added a brief discussion to the Discussion.*

> *Associated text from the Discussion: "This provides a mechanistic basis for a foundational, yet previously untested, theoretical tenet of speech categorization (Heald & Nusbaum, 2014), with implications for understanding and potentially remediating learning-related communication disorders like dyslexia that have been linked to auditory learning (Gertsovski & Ahissar, 2022) and auditory category learning (Gabay & Holt, 2015; Gabay, Roark & Holt, 2023) challenges. Furthermore, our finding that the specific learning trajectories by which listeners achieve categorization expertise leave a lasting imprint on*

cortical activation patterns suggests a neural basis for individual differences in speech categorization that persist even in neurotypical adults (Kim, McMurray, Sorenson, & Oleson, 2025).”

R1.6: In figure 4 I wasn't sure if Tonotopy 'vs.' Alien Identity/Size, was really 'versus' or a concordance of tonotopy and alien size, which is what I think you mean to relay with the labels.

AR-R1.6: *Thanks for flagging this - other reviewers also pointed out that this was ambiguous. We’ve now replaced all descriptions with your suggestion of ‘concordance of x with y’.*

R1.7: I was curious about the prefrontal involvement in the task, but have already commented on the brain wide results that would be useful for readers interested in seeing what is happening outside of auditory cortex. It would be good to have some discussion of these results if the authors deem them important to include in their study.

AR-R1.7: *We are excited about the prospect of the potential frontal and prefrontal involvement. But, as we mention above, examining this will require a different approach due to the lack of clear tonotopic organization in frontal regions. We have raised this in the Discussion (please see the text copied in* ***AR-R1.2****).*

R1.8:[...] I hope the authors and editors find the structured review useful, with only a few eminently addressable suggestions for improving an already strong manuscript.

AR-R1.8: *Thank you again for such a thorough and helpful critique, we hope that we have used the points well in improving the paper.*

Responses to Reviewer 2 (R2)

R2.0: This study investigates the neural mechanisms underlying auditory category learning, specifically examining how the auditory cortex adapts to diagnostic acoustic features. The study employed a novel "Alien" soundscape paradigm in which participants were trained to categorize complex sounds based on spectrotemporal patterns in specific frequency bands (High vs. Low). Using a "Concordance" metric that correlates voxel-wise frequency preference (tonotopy) with task-evoked activation, the study aims to determine whether learning selectively amplifies gain in category-relevant frequency channels. The primary finding is that category learning sharpens neural tuning: voxels preferring a specific frequency band show increased activity when that band contains diagnostic information. Furthermore, the authors report that this enhanced sensitivity persists even when the diagnostic dimension is irrelevant to the immediate task (i.e., during a "Size" judgment task), suggesting a stable, plasticity-driven change in cortical representation

rather than transient attentional modulation. Overall, I like the study ideas, and the study shows promising evidence in support of the interconnection. However, potential confounders or a lack of clarity may undermine the robustness of the evidence.

AR-R2.0: *Thank you for the supportive comments; as you rightly highlight (and as echoed by other reviewers), our description of methodological details and choices was insufficiently detailed and clear, which in turn made key decisions and results much harder to understand. We hope that new figures and explanations across the manuscript help to clarify the logic of the experimental approach.*

R2.1: My primary concern relates to the structural similarity between the localizer contrasts and the experimental contrasts, and whether the observed "concordance" is truly an effect of category learning or a result of shared acoustic sensitivities. The tonotopy localizer contrasts High vs. Low frequencies. Similarly, the category training contrast compares High-Diagnostic vs. Low-Diagnostic trials. Consequently, the "Concordance" metric essentially measures the correlation between these two very similar contrasts. While the authors attribute this overlap to learned attention, there is a risk that this is driven by shared sensitivity to frequency modulation (FM) or spectrotemporal complexity rather than tonotopic reorganization. If the diagnostic patterns contain specific FM sweeps that drive neurons in the corresponding frequency regions, the correlation could be a bottom-up acoustic effect. The authors need to rigorously demonstrate that the concordance is not simply due to the fact that the "High-Diagnostic" stimuli physically contain more acoustic energy or FM complexity in the high band, which would naturally correlate with a high-frequency tonotopy map regardless of learning.

AR-R2.1: *This comment really highlighted our lack of clarity in describing and illustrating a complicated stimulus set. We now emphasize the following points in revised figures, captions, and main text descriptions: (1) all alien sounds have simultaneously presented acoustic content <u>in both frequency bands</u>, where each band is made of a series of 3 'tone chirps'; (2) the chirps in the category-diagnostic frequency band are three acoustically different exemplars drawn from the same class of chirp (Fig 2A) and thereby possessing statistically structured regularity despite the variability; 3) the non-diagnostic band also contains 3 acoustically distinct chirp exemplars, but they are drawn pseudorandomly from all four classes of chirps (Fig 2B) and so there is no underlying regularity; 4) all chirps were drawn from the same pool of exemplars, with the only difference between frequency bands being the pitch shift and bandpass filtering applied to move them into the low or high band. In short, the spectrotemporal modulation characteristics are extremely similar between simultaneously-presented low and high frequency bands, and between category-diagnostic and non-diagnostic tone chirp triplets within those bands.*

As the reviewer rightly notes, it is also important to show that the statistical regularities in the alien-identity-diagnostic band do not immediately drive activation differences. This is demonstrated by the lack of identity-related activation differences in the 'alien size' trained cohort who had no experience in alien identity categorization. We re-emphasize this key point by adding

a new Figure 1 to better convey the tasks, revising the Figure 2 (formerly Figure 1) caption, adding a new text at the start of the Results section, and a revised Stimulus section in Methods.

The tonotopy mapping stimuli are very different from the alien sounds. They are created by concatenating 4-element 'mini-sequences' of sinewave tones into segments which slowly step up or down through the audible spectrum. The new Figure 1 now illustrates a spectrogram of these stimuli to highlight their acoustic composition.

R2.2: The study relies exclusively on post-training fMRI data. While the comparison between the "Identity-trained" and "Size-trained" (control) groups allows for between-subject inferences, the lack of a pre-training baseline makes it difficult to definitively claim that the observed effects represent plasticity or a change in organization. Without pre-training data, inherent differences in auditory cortical organization might have influenced results. A longitudinal design (Pre- vs. Post-scan) could have tracked this emergence within individuals. The authors should more transparently discuss this limitation and justify why a cross-sectional approach supports claims of cortex "reshaping'.

AR-R2.2: *Thanks to the reviewer for bringing up these questions. Here again, we did not make the hypotheses and relationship to experimental manipulation and training sufficiently clear, so have revised with this in mind.*

First, the alien identity training is designed to elicit 'learned salience' of the frequency-band-specific category information contained in the chirp triplets. Importantly category-disambiguating information is present in <u>both</u> frequency bands (spanning much of the audible spectrum), so we would not anticipate any overall change in sensitivity to a particular frequency band, nor changes in organization of auditory map topography. (This is unlike the classic studies of Kilgard, Merzenich, and others, where massed training with a single behaviorally-relevant frequency can evoke changes in the proportion of the tonotopic map represented by that frequency). Thus, even if the topography of tonotopic maps in the two groups showed substantial differences (which doesn't seem to be the case, as shown in Fig S1, this would not affect our ability to detect increased sensitivity to category-diagnostic information in these frequency bands, as the first-level (individual) analysis takes into account the voxelwise frequency preferences when calculating the concordance maps.

Second, despite very similar tonotopic mapping between the two training groups, across tonotopically mapped auditory cortex, the alien-size-trained group shows essentially no preferential activation for the frequency band with diagnostic alien identity information (Fig 7). By contrast, the matched alien identity+size trained group shows a very strong effect, with significant group differences in tonotopy/diagnostic frequency band activation concordance across all ROIs that show strong spectral preferences in tonotopy. (The subgroup analyses related to training performance also provide additional evidence).

The reviewer is of course correct that any group-based comparison can never definitively rule out potential pre-existing cohort effects (it is worth pointing out that pre-post group designs can also fall prey to such effects). Following the reviewer's suggestion, we have added a discussion of limitations to the discussion, as well as motivation for further work, in particular using neuroimaging to unveil the trajectory along which category learning shapes neural sensitivity on a minute-by-minute or session-by-session basis (in the style of Lim, Fiez, & Holt, 2019 and Ittipuripat, Cha, Byers, & Serences, 2017).

> *Associated text from the Discussion: "In future work, it will be important to show that the increased activation to identity-category-relevant frequency regions that is evident when they are not task-relevant (e.g. during size judgments) is not resulting from carry-over across experimental blocks. While we believe this is unlikely given the difficulty of the amplitude-conveyed alien size task (where weighting by spectrotemporal cues is counterproductive), it is possible that the activation patterns indicating acquired gain of the identity-diagnostic frequency band may diminish when not regularly reinforced via interleaving with blocks of the identity task.*
>
> *Use of a more naturalistic or captivating training paradigm (Deveau, Lovcik, & Seitz, 2014; Lim, Fiez & Holt, 2019; Whitton, Hancock, Shannon, & Polley, 2017) would promote extended training and consolidation, which would be particularly revealing in tracking slower learners like those who did not achieve full mastery in the present study. As well, since the present study establishes expertise-driven attentional gain to diagnostic dimensions, future studies could observe the emergence of this attentional gain as participants learn categories during scanning. This will reveal how category learning shapes neural activation on a minute-by-minute or session-by-session basis (in the style of Lim, Fiez & Holt, 2019 and Ittipuripat, Cha, Byers, & Serences, 2017). We also predict increasing learned salience such that identity category experts would exhibit further increases in gain for the identity-diagnostic band in the size categorization task, despite the task-irrelevant nature of frequency-band-delimited alien identity information in this task (see also Wang, Jendrichovsky, & Kanold, 2024)."*

R2.3: The manuscript includes analyses regarding individual differences, specifically splitting participants into subgroups (e.g., based on learning performance or strategy) to correlate behavior with neural concordance. Given the likely modest total sample size (typical of fMRI studies), subdividing the cohort raises significant concerns about statistical power. When the sample is split, the N for each subgroup is likely small. Correlations from small samples are unstable and influenced by outliers. The authors should provide scatterplots to confirm robustness. If subgroup N falls below thresholds for stable correlations, interpret those analyses cautiously or move them to supplementary materials as exploratory.

AR-R2.3: *Again, we have not communicated the analysis method effectively here. As the reviewer notes, we subdivide the entire alien identity expert sample into several groups based on*

behavioral learning outcome or trajectory. However, we never run correlation analyses between individual participant's behavior and neural concordance metrics (for exactly the reason the reviewer mentions). The concordance maps for each subgroup in Fig 6 are calculated as in the full sample, without any behavioral covariate; the group difference maps show the statistical comparison (FDR-corrected) of the two maps, using the method described by Hays (1994) and included at the very end of the Methods section.

In the revised manuscript, we now underline that these are group average or group comparison maps and do not include any behavior-activation correlation measure. Thank you for highlighting this.

> Associated text from the Results section: *"We then turned to the ROI-based regression approach using concordance maps. (Note that all maps show group averages or group comparisons, and do not include any behavior-activation correlation measure)."*

R2.3: Minor Concerns

R2.3.1: Logic Visualization: The logic underpinning the "Concordance" analysis and the relationships among the three tasks (Categorization, Tonotopy, Attention-to-Tonotopy) are complex. It is somewhat difficult to follow the progression of the analysis in the text alone. I strongly recommend adding a schematic figure or flowchart that visually depicts: How the maps were generated for each task. How the "Concordance" value is mathematically derived from the overlap of these maps. The specific hypothesis for the Experimental vs. Control group within this schematic.

AR-R2.3.1: *This is indeed a complex analysis, and likely not made easier to understand by having the relevant figures at the very end of the document. (The senior author at least finds this can be a major challenge for reviewing!). Figure 4 shows the analysis flow for map creation, including the regression equation for the concordance maps, and the subsequent conversion into the Z-maps. (The full description is near the end of Methods).*

*Reviewer 3 also mentioned Figure 3 (now Figure 4) needed work: we have followed your and his advice here, and have also highlighted the hypotheses for the group comparisons in the section "**Category expertise develops across training, with generalization to novel exemplars".***

> Associated text from the Results section ("Category expertise…"): *"This permitted us to test whether identity-training-induced changes in cortical activation associated with the frequency band carrying identity-diagnostic information exist, whether these changes are lacking in identity-naïve listeners, and whether they persist when task demands are redirected to category decisions based on amplitude. The Alien Size group is also important for identifying any bottom-up salience differences that may differentiate sounds from specific alien identity categories, independent of category expertise. By design, the*

> *stimulus exemplars (and their order) in the identity and size categorization blocks during the fMRI session were identical to avoid potential stimulus confounds."*

R2.3.2: ROI Definition: Please clarify exactly how the Regions of Interest (ROIs) were defined. Were they defined anatomically or functionally? If functional, were the data used to define the ROI independent of the data used for the main analysis to avoid circularity (double-dipping)?

AR-R2.3.2: *The full ROI creation and morphing procedure was a bit hidden in the depths of the Methods section as well as the Figure 3 caption, with only a brief mention in the Results. The revised manuscript now has more information in the main text (see below) and the figure caption (now Figure 4), with additional pointers to the full method.*

In short, we used a pre-existing set of cortical surface ROIs that tile the superior temporal plane (from Dick et al., 2017). These ROIs have were created to have roughly equivalent surface area, akin to a repeating quilt pattern; the individual ROI borders do not align with gyral landmarks or functional areas. We only included ROIs that lay within tonotopically organized auditory cortex, as determined by the spatial extent of the full cross-subject-average tonotopic map (this is shown by a purple outline in the figure). Each ROI in the set was morphed from a single participant's surface (from the Dick et al. 2017 study) to the cortical surface of each participant in the present study via cortical-surface-based spherical registration (Fischl, Sereno, Tootell, & Dale,1999) in Freesurfer. Finally, each ROI was sampled back to the individual's affine-registered native EPI space.

Therefore, ROI shape and selection was completely independent of the contrasts of interest, and also included all of 'classic' tonotopically mappable cortex.

> *<u>Associated text from the Results section</u>: "The 'concordance map' approach (Dick et al., 2017) described in the introduction allows us to capture consistent regional differences in responses across participants while retaining frequency sensitivity at a voxel-wise level, despite tonotopic map inter-subject heterogeneity. Here, each individual's auditory-responsive cortices are parcellated into a set of small, roughly equal-area ROIs that are projected via curvature-aligned spherical registration from a participant surface in Dick et al., (2017) onto each individual's auditory cortical surface (**Fig 4A,** see **Methods-Analyses**). (The ROI borders are not designed to align to gyral anatomy or functional fields). Each ROI exhibiting frequency selectivity at the group level (as estimated using the group-average tonotopic map) is sampled into the cortical ribbon in the individual's native volumetric EPI space (**Fig 4B)**. Analogous to the high-minus-low frequency selectivity contrast from the tonotopy runs, voxel-by-voxel beta coefficients relating to activation during alien identity categorization (involving the high- and low-frequency category-diagnostic bands) are computed and a high-minus-low frequency band difference in beta coefficients is calculated for each voxel. These parallel difference scores permit us to fit a regression model across all participants within each ROI (with participant as a random factor, **Fig 4C**) revealing the ROI-wise concordance between the voxelwise*

> *frequency selectivity evoked by tonotopy stimuli, and the hypothesized, category-specific frequency-band selectivity driven by the alien identity learning task (**Fig 4D** and **Methods-Analyses**). (All of the inferential statistics for each subgroup, as well as cross-subgroup comparisons, are reflected in the statistical maps, with outlines showing FDR-corrected significance in the same way as with the full groups).”*

R2.3.3: Acoustic Controls: In the "Size" task, were the stimuli acoustically identical between the "Big" and "Small" categories aside from amplitude? It would be helpful to see a brief analysis confirming that the High and Low frequency bands were energetically balanced across the different conditions to ensure no low-level acoustic confounds influenced the "Size" task neural data.

AR-R2.3.3: *Amplitude was manipulated on an exemplar-wise basis after the two frequency bands were combined, so amplitude manipulations scaled equally over the entire spectrum. Not only were stimuli acoustically identical across Big and Small alien size categories aside from amplitude, but they were also identical across the Size and Identity tasks, with the only difference being the task that participants performed on the stimuli. We now emphasize when amplitude scaling takes place during acoustic processing in the Stimulus section of Methods.*

Thanks much to the reviewer for the detailed comments, and for the significant time it must have taken to review this involved manuscript.

> *Associated text from Methods (Stimuli): “In addition, we also imposed orthogonal amplitude variation (between 62-80 dB) on copies of each of these 592 sounds. We manipulated amplitude on an exemplar-wise basis after the two spectral bands were combined, so amplitude manipulations scaled equally over the entire spectrum. This met two goals: to increase variability and task difficulty, and to permit creation of two Alien-Size categories, with "Big Alien" exemplars falling between 72-80 dB sound intensity, and “Small Alien” exemplars falling between 62-70 dB (all reported dB referenced to $2*10^{-5}$ and measured in Praat). Thus, each exemplar could be categorized along identity (spectrotemporal, frequency-band-specific) or size (amplitude) dimensions. Not only were stimuli acoustically identical across Big and Small alien size categories aside from amplitude, they were also identical across the Size and Identity tasks, with the only difference being the task that participants performed on the stimuli.”*

Response to Reviewer 3 (R3)

R3.0 (Patrik Wikman signed his report): The manuscript studies how learned category membership, driven by frequency specific diagnostic information, affects the auditory cortex representation of the learned categories. One participant group learned that spectral attributes in one of two frequency channels (high vs. low) differentiated novel sound objects called "space aliens", while the other frequency range did not. Participants, thus, learned by experience driven learning in a 2ACF space alien identity task to pay attention to either high frequency spectral attributes or low spectral attributes of the objects. In addition the same participants performed a space alien size task to evaluate the size of the alien based on amplitude. In the size task the identity of the alien was irrelevant.

During fMRI tonotopic gradients of auditory cortex parcels were evaluated using two tasks, passive frequency selectivity and attention-driven frequency selectivity. The same participants also performed the 2ACF task in the scanner. The main result of the study is that the frequency selectivity during the space alien identity classification was predicted by the parcels frequency selectivity probed both with the passive and active tonotopy task. This effect was found both in the identity task, where the information was task relevant, and in the size task, where it was irrelevant. Furthermore, the level of this correlation depended on the achieved expertise and the learning rate of the participant. Importantly, similar findings were not found in another group of participants that had not learned to pay attention to the high vs. low frequency bands that contained the diagnostic information about identity. Thus, the authors argue that an important facet of category learning is to learn to pay attention to the category differentiating low-level features and that this produces task-dependent and plastic responsiveness in the auditory cortex.

My assessment is that the results are interesting and novel. However, the design and analysis methods are complex and consequently the authors could help the reader with adding in-text citations throughout the manuscript (e.g., referring to the relevant chapter in the methodology in the results). Below I have outlined my specific suggestions:

AR-R3.0: *Thank you for such a clear summary of the work, and for the positive comments. We outline the revisions in response to your helpful suggestions below, and have also placed pointers throughout to the relevant sections.*

R3.1: The tasks, training days, tasks in scanner of the groups etc. are scattered through the Methods and Results text, a flow chart in the beginning of the Results would help the reader to understand the complex design.

AR-R3.1: *We have wholeheartedly taken up this excellent suggestion, and have created a new schematic (Fig 1) showing the entire design and procedure, including a flow chart.*

R3.2: Figure 3 is key to understanding the analysis approach. However, I find the figure difficult to follow. I understand that the task-concordance analyses have been used in the group's prior work, but since it is a fairly novel approach I would make it clearer here.

AR-R3.2: *Reviewer 2 also found the description and figure hard to understand; we outline our specific changes in* ***AR-R2.3.2.****, copied here for ease:*

We used a pre-existing set of cortical surface ROIs that tile the superior temporal plane (from Dick et al., 2017). These ROIs have were created to have roughly equivalent surface area, akin to a repeating quilt pattern; the individual ROI borders do not align with gyral landmarks or functional areas. We only included ROIs that lay within tonotopically organized auditory cortex, as determined by the spatial extent of the full cross-subject-average tonotopic map (this is shown by a purple outline in the figure). Each ROI in the set was morphed from a single participant's surface (from the Dick et al. 2017 study) to the cortical surface of each participant in the present study via cortical-surface-based spherical registration (Fischl, Sereno, Tootell, & Dale,1999) in Freesurfer. Finally, each ROI was sampled back to the individual's affine-registered native EPI space.

Therefore, ROI shape and selection was completely independent of the contrasts of interest, and also included all of 'classic' tonotopically mappable cortex.

R3.2.1: Especially I did not understand how many category-betas per participant were extracted and how many tonotopy-betas were extracted and how they were paired. A vector image showing how the regression was conducted would help in understanding this, you can use RSA studies as a template for this.

AR-R3.2.1: *Using the RSA model is a great idea; we've tried to incorporate this approach into the figure (now Figure 4). Regarding the specific question about the number of betas extracted per participant: there is a regression model fit for each ROI, where for each participant, the number of betas per task equals the number of voxels in the native-space projection of that ROI. The voxelwise beta coefficients for the tonotopy task are from the general linear test (GLT) of high minus low frequency bands; correspondingly, the coefficients for the alien identity task are from the GLT of high minus low category diagnostic bands. Betas are always paired voxelwise for all cross-task concordance maps. We have inserted this explanation into the Methods section, thanks for pointing out that this was not clear.*

> *Associated text from the Methods section (Analyses):* *"Although there is a macroscopic cross-participant similarity in large-scale tonotopic gradients, there is considerable cross-participant variability in frequency preference at a finer grain, particularly when voxels are aligned by cortical folding patterns (Besle et al., 2018; Moerel et al., 2014). Thus, to measure regional encoding and cross-task concordance in frequency selectivity while*

*taking this variability into account, we calculated the degree of concordance in cross-task voxel-wise frequency selectivity in a set of small cortical surface ROIs defined in a previous study (Dick et al., 2017, see **Fig 4**). Here, we limit our analysis to those ROIs where tonotopic selectivity was observed at the group level in our alien-identity categorization experts. For each participant, the ROIs that tessellate tonotopically organized auditory cortex (37 per hemisphere) were projected from a single participant to the unit icosahedron (sphere_reg), then projected again to each curvature-aligned participant's surface, and finally to the bbregister-aligned native space 2 x 2 x 2 mm EPI space using Freesurfer's mri_label2vol.*

For each ROI, cross-task concordance was evaluated via regression analysis, where all subject-wise beta coefficients in one task (e.g., $b_{tonotopy(high\text{-}low)}$) were used to predict the high-low betas in another task (e.g., $b_{attention\text{-}tonotopy(high\text{-}low)}$); each regression model included random intercepts for each participant. In other words, there is a regression model fit for each ROI, where for each participant, the number of betas per task equals the number of voxels in the native-space projection of that ROI. The voxelwise beta coefficients for the tonotopy task are from the general linear test (GLT) of high minus low frequency bands; correspondingly, the coefficients for the alien identity task are from the GLT of high minus low category diagnostic bands. Betas are always paired voxelwise for all cross-task concordance maps."

R3.2.2: Note also that since the directed attention task has not been introduced in the text the author cannot understand what you are referring to. Thus, I would add a in-text reference and introduce all tasks in the beginning of the results (see also comment 8).

AR-R3.2.2: *Fully agreed - the frequency-selective attention task is now presented in the Introduction, and in the Figure 1 task flow schematic at the beginning of the Results section.*

R3.3: Page 3. please define what you mean by endogenous attention. I would also contrast it with exogenous attention, as you suggest that after learning the frequency information may cause learned salience. Regarding such effects you could also discuss prior studies that have shown similar experience driven salience effects (see e.g., Addleman, Douglas A Jiang, Yuhong V, 2019, Experience-driven auditory attention, Trends in Cognitive Sciences)

AR-R3.3: *Thanks for flagging; we've reworked the presentation of attention in the Discussion, and included the Addleman et al. article which is helpful. Regarding endogenous and exogenous attention, we decided to edit out mention of these terms because they were not essential for the theoretical basis here, and we also did not include a specific exogenous attention condition as a contrast.*

<u>Associated text from the Discussion:</u> "The cross-group differences in the present study demonstrates that the alignment of feedback with a diagnostic dimension played a role in eliciting the cortical selectivity toward those dimensions. These patterns of acquired attentional gain in human auditory cortex connect with nonhuman animal electrophysiological studies that observe that the task-relevance of an acoustic dimension drives changes in receptive fields consistent with attentional gain (Atiani et al. 2014; Bagur et al., 2018).

In this vein, Yin et al. (2020) present evidence for top-down modulation of auditory cortex by frontal cortex in ferrets, along with induction of intrinsic auditory cortical patterns related to acquired categories that persist even in passive listening. Though we limited our investigation specifically to tonotopically mappable temporal cortex, the regions we observe to be modulated by category learning receive rich top-down feedback inputs, and exhibit modulation by behavioral context that extends beyond basic representation of perceptual dimensions (e.g., Zhong et al. 2019). Such top-down modulation has been well-documented in nonhuman animal electrophysiology (particularly in the visual system, e.g., Freedman et al. 2003; DeGutis and D'Esposito, 2007) and may interact with the persistent changes to auditory cortical representation arising from category learning that we observe. However, as Addleson and Jiang (2019) note, the neural mechanisms of shifts of experience-driven attention may or may not be supported by the same frontoparietal regions as goal-driven attention. The lack of reliably definable tonotopic maps in frontal and other non-auditory regions using 3T fMRI makes it challenging for the present study to adjudicate this question. However, very recent human intracerebral stereoelectroencephalography (sEEG) reports prefrontal and inferior frontal spectrotemporal selectivity at multiple electrode sites (Hullett, Leonard, Gorno-Tempini, Mandelli, & Chang, 2026) suggesting that ultra-high-resolution fMRI using the present approach may yield fruitful insights."

R3.4: Page 4. The space alien identity and space alien size differentiation was not very clear while reading the Intro. First I thought space referred to spatial information, not space as a concept. Please add a couple of sentences explaining what is meant by these so that the reader understands the idea from the start.

AR-R3.4: *We can definitely see how the multiple senses of 'space' could lead to confusion - we now just call them 'aliens', and have also expanded on the alien identity versus size differences in the beginning of the Results section and in the Methods section.*

<u>Associated text from beginning of Results:</u> "These stimulus characteristics impose several key demands on learning: (1) because chirp triplets appear simultaneously in two distinct frequency bands but statistically structured patterns diagnostic of alien identity occur in only one band, listeners must discover the relevant regularity in the context of competing

sounds; (2) chirp triplets within the category-diagnostic frequency band are acoustically variable yet possess an underlying spectrotemporal regularity that maps onto alien identity through feedback; (3) because this regularity differs across aliens, listeners must not only identify the diagnostic frequency band but also learn to map specific spectrotemporal patterns within each frequency band onto alien identity; (4) overall amplitude varies across sounds, such that learning alien identity requires generalization across identity-irrelevant amplitude variation; (5) learning takes place under adverse listening conditions, with sounds embedded in scanner noise throughout, including during behavioral training outside the scanner."

R3.5: In the figures (e.g., Fig 4) you use the abbreviation vs. (versus) but if I understood correctly you mean that something has been used as an regressor for something, please change this as it is misleading (one thinks its a contrast between tasks).

AR-R3.5: *Multiple reviewers pointed out this was confusing; we've followed Reviewer 1's suggestion in referring to them simply as the concordance of the two tasks.*

R3.6: Page 9. Could you explain what the concordance between the identity and size tasks was supposed to indicate, currently I do not understand the added value of this regression analysis.

AR-R3.6: *Thanks for bringing this up. Upon some reflection, we also agree this analysis doesn't provide any additional information regarding the theoretical questions (and was not discussed further) so have taken out the few sentences and subfigure relating to it.*

R3.7: Page 11. Group 1 and Group 2 have different levels of exposure to the space aliens, thus, could it be that some of the discrepancies in the neural effects between the groups are explained by exposure. I would argue that the fact that task achievement modulated the correspondence in group 1 (fig 5) argues against this, but could be good to mention this in the limitations.

AR-R3.7: *We agree completely, and now explicitly acknowledge this as a limitation in the discussion, indeed borrowing some of your clear phrasing.*

<u>Associated text from the Discussion:</u> "These changes were driven by the specific demands of learning, and not by mere exposure to statistically structured sounds. Participants who trained to use frequency-band-diagnostic information to categorize alien identity exhibited neural selectivity consistent with acquired attentional gain to the diagnostic frequency bands. But those who learned the amplitude-signaled size categories across the very same sounds, thus experiencing the identity-diagnostic spectral information without learning to use it to categorize, did not. (One caveat to this conclusion is that Alien Identity+Size-trained participants had overall much more exposure to alien sounds than Alien Size-trained participants, which could potentially drive differences. However, the fact

that neural selectivity was strongly driven by degree of behavioral expertise within the Alien Identity+Size-trained group would argue against this explanation.)

Although humans and other organisms exhibit an ability to learn across passive exposure to statistically structured input (Saffran & Kirkham, 2018; Wilson et al., 2015), studies have observed that complex acoustic exemplars that model the complexity of speech are not well learned over passive exposure (Wade & Holt, 2005, Emberson, Liu, & Zevin, 2013). Yet alignment of these statistically structured sounds with active behavior – even incidentally and without overt feedback – can rescue category learning (Lim, Fiez & Holt, 2019; Roark, Lehet, Dick, & Holt, 2022). The cross-group differences in the present study demonstrates that the alignment of feedback with a diagnostic dimension played a role in eliciting the cortical selectivity toward those dimensions. These patterns of acquired attentional gain in human auditory cortex connect with nonhuman animal electrophysiological studies that observe that the task-relevance of an acoustic dimension drives changes in receptive fields consistent with attentional gain (Atiani et al. 2014; Bagur et al., 2018)."

R3.8: Page 12. The description of the attention driving task should briefly be outlined in the beginning of the results, now its a bit confusing see also comment 1.

AR-R3.8: *We definitely did not describe the attention tonotopy task sufficiently; we've added a new Figure 1 that provides a schematic overview of the study protocol at the start of the Results section.*

R3.9: Could you discuss briefly in the discussion why concordance in the tonotopy-identity, tonotopy-size, and tonotopy-attention driven as well as attention driven-identity yield different patterns of results, do they bear any theoretical significance, now in the discussion the analyses are described as yielding fairly similar patterns.

AR-R3.9: *Thanks for flagging this. We tread a bit too lightly on this topic for fear of over-interpreting qualitative map differences. We've now added some thoughts in the discussion, particularly regarding the attention task and current limitations for interpretation.*

<u>Associated text from the Discussion:</u> "We should emphasize that auditory cortical fMRI activation differences driven by categorizing alien identity are decidedly not identical to those evoked by overt attention to pure tones with frequencies in the higher or lower category-diagnostic spectral bands. Although tonotopy concordance with alien identity and attention tonotopy activation is overall quite similar (with both showing particularly strong concordance in left anterior ROIs, and to a lesser extent along the right STG), direct concordance between alien identity and attention tonotopy high-low frequency difference maps is greatest along bilateral STG, and not the anterior-most left ROIs. While our previous work (Dick et al., 2017) did show particularly consistent attentional tonotopic modulation along many of these STG regions, it is unclear what is driving this left anterior

difference. A limitation for interpretation lies in attention tonotopy task performance; the attention task was difficult, particularly for some participants, and lapses in sustained attention to one or both frequency bands by a subset of participants could influence the robustness of results. (The task was also run at the end of the Follow-up studies would benefit from a longer training regime for the attention task, similar to that for the alien identity one, to assure consistent performance across runs."

R3.10: Page 20. Please indicate whether the exemplars used during the fMRI experiment were novel or had been used in training and whether the exemplars for the size experiment were different from the ones in the identity experiment.

AR-R3.10: *We have clarified this in the Methods-MRI Procedure section of the revised manuscript: the 80 2AFC categorization fMRI stimuli were drawn from the pool of 80 generalization exemplars encountered in training. Participants had received no feedback with these stimuli, and never made a 2AFC decision with them. Each of the 80 exemplars was encountered at two different amplitudes.*

By design, the exemplars (and their order) in the identity and size task blocks were identical to avoid potential stimulus, context, and order confounds. This information was only tucked in the depths of the Methods section. We have reinforced this point in the Methods, as well as mentioning it in the relevant part of Results. (This also relates directly to the final comment below).

<u>Associated text from Methods (MRI Procedure):</u> "Next, for Alien Identity+Size participants, there were six runs (10 blocks each, 8 trials/block) of 2AFC categorization. Across runs, 20 blocks involved categorization of alien identity across the high diagnostic frequency band, 20 involved identity categorization across the low frequency band, and 20 involved size categorization across amplitude. Use of the 2AFC (rather than 4AFC) categorization task in the scanner afforded several benefits: a) increasing power to differentiate across higher and lower frequency bands by minimizing switches within a block; b) reducing saccades across distinct visual aliens; c) constraining each block to two categories making identity and size tasks completely parallel. Our prior behavioral study demonstrated no learning advantage for even explicit instruction indicating the diagnostic frequency band (Obasih et al., 2023). A pseudorandomized block order common across participants assured that blocks reliant on the same diagnostic dimension did not occur consecutively. Overall block order in runs 1-3 repeated in runs 4-6, but with trial order shuffled, and with the amplitude set to a different level within the 'big' or 'small' amplitude range. In all, there were 320 alien identity categorization trials (80/category, with equal exemplars from the two size categories) and 160 alien size categorization trials (with equal exemplars from the four identity categories). Note that the 2AFC categorization fMRI stimuli were drawn from the pool of 80 generalization exemplars encountered in training. Prior to the fMRI experiment, participants had received no feedback with these stimuli, and never made a 2AFC decision with them. Each of the exemplars was encountered at two different amplitudes."

R3.11: Page 20: "To facilitate comparisons between the alien identity and alien size categorization blocks, 20 of the 40 blocks that appeared during the alien identity task also appeared in the alien size task, with the same fixed trial order within the block." Can you explain how this facilitates comparison.

AR-R3.11: *As noted above, this allows us to keep the acoustic input identical and only change the task so that any potential frequency-band-selective effects in the 'size' task due to stimulus, context, or order properties will also be present in the 'identity' task. We now emphasize this point in the Results section as well as the Methods section.*

> *<u>Associated text from the Methods (MRI Procedure) section:</u> "To facilitate comparisons between the alien identity and alien size categorization blocks, 20 of the 40 blocks that appeared during the 2AFC alien identity task also appeared in the 2AFC alien size task, with the same fixed trial order within the block. By design, the exemplars (and their order) in the identity and size task blocks were identical to avoid potential stimulus, context, and order confounds. Half of the alien size blocks appeared before their corresponding alien identity blocks, and half appeared after. Each block began with a 3-sec visual display of the task ("Alien Identity" or "Alien Size") followed by 1.5 sec for the audio and a 1.5 sec response window."*

Finally, we would like to thank Dr. Wikman for the extremely helpful comments, and for taking such care in critiquing the manuscript and research.

Responses to Reviewer 4 (R4)

R4.0: This project addresses the important issue of auditory cortical processing correlates of acoustic category learning. Strengths include a large sample size, at least overall, strong and clear behavioral evidence of category learning. This is a rich data set, and well-worth publishing. In general, it is a good match to PLOS 1, and provides useful information to the field. Some weaknesses are described below.

AR-R4.0: *Thank you for these comments.*

R4.1. A major weakness is not having longitudinal measures of fMRI (baseline before training as well as post-training). This limits inferences about category learning-related changes in activation responses. The use of sub-groups is helpful, by dividing according to coarse differences in learning across days, but this is also only reflected in the one fMRI session after training.

AR-R4.1: *As we discuss in our response to Reviewer 2 (**AR-R2.2**), we did not describe the motivation for the design clearly enough. Our research question is centered on the outcomes of expertise, and on the emergence of auditory category representations – specifically, acquired attentional salience to category-diagnostic acoustic dimensions. We addressed these by using (as R1 and R3 highlighted) a well-powered and well-matched control group without this expertise, along with within-group analysis of high-density behavioral learning trajectory data. In the revised Discussion, we note that now that we have established the basic finding, we can investigate the learning mechanisms that drive the system to this point by observing the emergence of learning during scanning.*

> *Associated text from the Discussion: "In future work, it will be important to show that the increased activation to identity-category-relevant frequency regions that is evident when they are not task-relevant (e.g. during size judgments) is not resulting from carry-over across experimental blocks. While we believe this is unlikely given the difficulty of the amplitude-conveyed alien size task (where weighting by spectrotemporal cues is counterproductive), it is possible that the activation patterns indicating acquired gain of the identity-diagnostic frequency band may diminish when not regularly reinforced via interleaving with blocks of the identity task.*
>
> *Use of a more naturalistic or captivating training paradigm (Deveau, Lovcik, & Seitz, 2014; Lim, Fiez & Holt, 2019; Whitton, Hancock, Shannon, & Polley, 2017) would promote extended training and consolidation, which would be particularly revealing in tracking slower learners like those who did not achieve full mastery in the present study. As well, since the present study establishes expertise-driven attentional gain to diagnostic dimensions, future studies could observe the emergence of this attentional gain as participants learn categories during scanning. This will reveal how category learning shapes neural activation on a minute-by-minute or session-by-session basis (in the style of Lim, Fiez & Holt, 2019 and Ittipuripat, Cha, Byers, & Serences, 2017). We also predict increasing learned salience such that identity category experts would exhibit further increases in gain for the identity-diagnostic band in the size categorization task, despite the task-irrelevant nature of frequency-band-delimited alien identity information in this task (see also Wang, Jendrichovsky, & Kanold, 2024)."*

R4.2. I could not find a statistical analysis of the sub-group activation patterns, potentially due to low power. This limits inferences on how activations relate to behavior.

AR-R4.2: *All of the inferential statistics for each subgroup, as well as cross-subgroup comparisons, are reflected in the statistical maps, with outlines showing FDR-corrected significance in the same way as with the full groups. We now make sure to mention this in the main text. (The group statistical comparisons were probably hard to see and keep track of because the document for reviewers has all of the figures separated out at the very end of the*

document, requiring a lot of going back and forth across pages. We have included a 'reviewer-friendly version with embedded figures as well in hopes this helps reading).

> *Associated text from Results (Alien identity learning…): "(All of the inferential statistics for each subgroup, as well as cross-subgroup comparisons, are reflected in the statistical maps, with outlines showing FDR-corrected significance in the same way as with the full groups)."*

R4.3: The main analyses used the concordance measure, which is a difference score.

The Authors should more clearly consider other differences in stimuli besides frequency bands of interest (diagnostic vs. non-diagnostic) that may influence the concordance measure (e.g. amplitude modulation and frequency modulations). This issue probably receives some control by having categories based on high- or low-frequency information, but does not rule out other stimulus features that may covary with category. The Authors asserted that "The differential cortical activation across the high versus low frequency bands cannot easily be attributed to 'bottom-up' acoustic salience, as all stimuli possessed information in each frequency band in addition to amplitude variation." (p. 9). This may be true, but the burden of proof is on the Authors to make a more compelling argument. Having information in different frequency bands vs. not having information that covaries with category are two different ideas.

AR-R4.3: *As also became evident with the critiques of the other reviewers, this comment was very helpful in showing that we have not communicated the details and logic of the stimuli or analysis clearly. An initial clarification: the concordance measure is the result of a regression between two differences, for instance, the difference in activation evoked by high minus low frequency bands in the tonotopic task on the one hand, and the difference in activation evoked by alien sounds with identity information in the high-frequency band, minus those with identity information in the low-frequency band.*

Regarding the relationship of other acoustic features to alien identity categories, the revised manuscript now includes more explicit descriptions about how amplitude, and amplitude and frequency modulation are balanced across identity-diagnostic and non-diagnostic frequency bands and exemplars. In short, the three tone chirps from different categories in the category-non-diagnostic band are drawn from the same pool as the three from the same category assigned to the diagnostic band. By design, there is significant acoustic variability (e.g., amplitude, pitch, pitch excursion, etc) across exemplars from the same category as well as across categories, making the category learning particularly challenging. Because category identity depends on statistical regularities in tone contour (as can be observed in tone languages and birdsong, for instance), the consistency of the direction and shape of the tone contours will differ between diagnostic and non-diagnostic bands, but the same contours do exist across both bands.

The key finding here is that, in contrast to the alien identity + size trained group, the alien size only group - who have not learned the utility of these contours - shows essentially no sign of differential activation for the category-diagnostic band containing the category-diagnostic contours. Indeed, if anything, the majority of auditory cortex shows a slight numerical advantage for the non-diagnostic band, which is what we would expect from the many studies that show greater stimulus variability leads to greater activation.

R4.4: In general, the Discussion section needs to scrutinize the current results, including the above weaknesses. In its current form, the Discussion quickly moves to covering the research of others, without carefully considering what was learned from the present study.

AR-R4.4: *Thanks for the suggestion. We have added new paragraphs and descriptions synthesizing the key points from the present study in the Discussion. However, we have tried to retain links to several literatures throughout the discussion to make it clear what motivated the questions we asked.*

R4.6: The Authors may want to comment further on hemispheric differences, perhaps with quantitative comparison. There may be a connection with the diagnostic stimulus features being variations of F0 Mandarin chirps and left vs. right hemisphere auditory processing for segmental and suprasegmental information, respectively.

AR.R4.6: *Thanks for suggesting this idea. We now comment briefly on the hemispheric differences in the Discussion (see below). However, since we did not explicitly design our study to investigate hemispheric differences and have not made a priori predictions we have not conducted formal analyses. A careful follow-up investigation of hemispheric contributions would require controls for motor response demands and attentional as well as bottom-up acoustic controls known to contribute in past studies to hemispheric differences. Finally, because the ROIs were created and morphed on a hemisphere basis (rather than morphing from one hemisphere to the other), we cannot make quantitative ROI-specific comparisons across hemispheres.*

> *Associated text from Discussion: A further limitation is that the ROIs were created and morphed on a hemisphere-specific basis, thereby precluding quantitative cross-hemisphere comparisons of qualitative differences, such as the qualitatively higher concordance between attention tonotopy and alien identity tasks in the right compared to left STG.*

Incidentally, we would note that the origins of our stimulus components as Mandarin lexical tone contours don't really allow us to make hemispheric predictions based on the (still highly debated) literature on segmental, syllabic, and/or prosodic contours because (1) the original speech was filtered to strip away all acoustic information except the F0 contour, rendering it nonspeech; (2)

these contours were then spectrally shifted to fall well outside boundaries of speech F0; (3) exemplar duration (400ms) falls roughly in ‘syllabic’ duration, but listeners must integrate these contours across all three exemplars to extract alien identity and size; (4) most importantly, we do not allow any variability in exemplar length so have no means of contrasting contours that vary in duration within an experiment.

Finally, we would like to thank Reviewer 4 for the time and thought in writing this helpful review.
