# Supplementary material for: Category learning drives cortical attentional gain to diagnostic dimensions of perceptual information
Source: PLoS Biol. 2026 Nov 12;24(11):e3004076. doi: 10.1371/journal.pbio.3004076 (PMC13759068; doi:10.1371/journal.pbio.3004076)
Supplement: Supplementary file 2 [file pbio.3004076.s006.pdf]

August 27th, 2026

Dear Dr. Schnell,

Thank you for the opportunity to respond to the remaining points on our manuscript. We seriously appreciate the time and thought the reviewers have taken to help us improve it.

Below are pasted verbatim your editorial guidance **(Editor),** our author response to editor ***(AR-E)*,** then comments and responses to Reviewers 3, 4, and 2 in that order, with each reviewer comment indicated by reviewer number and comment (e.g., **R2.1**) followed by our author response in italics to that point (e.g., ***AR-R2.1***).

Editor: In light of the reviews, which you will find at the end of this email, we are pleased to offer you the opportunity to address the remaining points from the reviewers, in particular Reviewer 2 via a thorough discussion of the concerns raised by Reviewer 2 in the discussion section of the manuscript if you are unable to address them experimentally. We will then assess your revised manuscript and your response to the reviewers' comments with our Academic Editor aiming to avoid further rounds of peer-review, although we might need to consult with the reviewers, depending on the nature of the revisions.

AR-E: *We were pleased that Reviewers 3 and 4 were satisfied with our revisions, and thank you again for the opportunity to address these points from Reviewer 2, as laid out after each point in turn below.*

For convenience, here is an overview of manuscript revisions made in response to R2's remaining points:

1) *We revised the subgroup analyses comparing early versus late experts and higher versus lower achievers to use R2's suggested fully multilevel modelling approach, along with FDR correction as previously described. Both these analyses again show significant differences between groups.*
2) *We also revised the group analyses comparing alien identity+size-trained early experts to the alien-size trained group, now using multilevel modelling as suggested by R2. As previously described for the original analysis, this new analysis shows significant differences between groups (FDR-corrected).*
3) *Responding to R2's suggestion regarding group sizes and selection, we have performed and reported an additional multilevel model comparing the full group of alien identity+size trained cohort to the full cohort of alien-size-only trained participants. As with the subgroup comparison originally reported, there are significant and substantial group differences in concordance between alien-sound- and tonotopy-evoked activation. This serves as an additional piece of evidence that training on alien identity – which requires segregating auditory cues based on spectral position – has a differential effect on neural tuning and generalization.*
4) *We have added and/or revised text throughout the manuscript to tighten claims and descriptions regarding training effects and stimulus composition, added caveats about the strength of conclusions given subgroup sample sizes (from the individual differences*

analysis), and pointed to the literature on repetition priming that bears on potential effects of within-category versus between-category repetition inherent to our stimuli.

We hope that these changes along with the responses below will address remaining concerns, and would again like to thank you and the reviewers for such helpful and productive comments.

##

Reviewer 3 (signed by Patrik Wikman): I want to thank the authors for the significant amount of work they put into clarifying the manuscript. I feel that especially the changes to Figure 3 and the new figure 1 makes it much easier to understand the setup.

AR-R3: *We are really pleased that Dr. Wikman found these changes helpful. We would like to thank him again for such helpful and detailed suggestion.*

##
Reviewer #4: The authors did a good job with revisions. No additional comments

AR-R3: *We are grateful for the reviewer's previous suggestions and are happy that they feel the revisions were successful.*

##
Reviewer #2:

R2.1: I appreciate the authors' efforts to clarify the design and analyses. However, my central concerns remain unresolved and affect the main conclusions.

AR-R2.1: *We thank the reviewer for their comments on the initial submission, which helped to guide the changes we made. We address each of their concerns as laid out below, as well as the new analyses and text changes in response to the reviewer's new comments.*

R2.2: First, the learning-trajectory analyses remain underpowered. Dividing participants into Early/Late Experts or High/Low Achievers is an individual-differences analysis because the claim is that neural concordance varies with learning trajectory. These comparisons contain only 16-17 participants per subgroup, and the High/Low analysis discards the middle tercile. Many voxelwise observations cannot compensate for the small number of independent participants. Small task-fMRI samples yield unstable group effects, and categorizing continuous variables further reduces information and power.

AR-R2.2: *There are several points to address here. First, regarding power, we have shown significant, multiple-comparisons-corrected effects in each subgroup, as well as in the cross-group analyses, now newly analyzed as per the reviewer's suggestion below to model group in the mixed-level model. (Thank you for this suggestion, it is a much more straightforward approach). Thus, there is sufficient power to detect at least the most prominent subgroup differences, and*

the pattern of results from the subgroups are quite similar, even for the slower and less expert groups who have few participants in common.

Second, there are important design features that reduce uncontrolled cross-participant variability in behavioral performance and strategies in the scanner, and also reduce within-participant behavioral variability across the six runs of the alien identity & size tasks. The main fMRI cohort (N=54 => N=49 after basic fMRI quality control) was selectively drawn from 95 participants who completed 5 days of training on a well-characterized behavioral task (originated by Obasih et al., 2025, with ~300 participants tested with different experimental variants). Participants were only recruited for fMRI if they achieved very good performance (75% accuracy in both diagnostic spectral bands) on this difficult task by the end of training. Per participant, there were 320 trials of the alien-identity fMRI task (equally split between high- and low-diagnostic bands) and 160 trials in the alien-size fMRI task. (These N and trial numbers/condition also place the current study in the ~80-90th+ percentile of experimental fMRI studies in influential journals given a relatively recent survey of the field (Szucs & Ioannidis, 2020). In short, we have made considerable effort to control variability across participants at multiple design stages, which will increase power to detect.

Third, regarding a grouped versus continuous individual differences analysis, we deliberately chose to group into terciles because of the nonlinearity in the behavioral measures, the likely nonlinear relationship between behavior and our measure of category-relevant activation, and the difficulty in accurately modelling these nonlinear relationships with the N available to us. Another possibility would have been a split-half analysis, but then each group would be composed of participants with quite different learning trajectories and outcomes.

These points notwithstanding, we have done our best not to overstate any results based on these subgroups. We have also added additional caveats and future directions to the Discussion section to emphasize this:

Results: "An important caveat to these findings is that these behaviorally-defined subgroups are relatively small compared to the full sample size, so follow-ups with larger N samples will be helpful for interpreting the strength of these effects."

Discussion: "Greater training time, along with a larger N for fuller sampling of individual differences, would allow for better understanding of potential non-linearities in the relationship between learning and changes in category-related neural sensitivity."

R2.3: Second, the acoustic alternative has not been excluded. The diagnostic band contains chirps from the same category, whereas the nondiagnostic band combines chirps across categories; these conditions therefore differ in spectrotemporal regularity and coherence. Explaining that the chirps came from the same overall pool is insufficient without quantitative acoustic controls. The null result in the smaller control group is not evidence of acoustic equivalence.

AR-R2.3: *To address the last point first, the manuscript does not claim or argue that the diagnostic and non-diagnostic bands are acoustically equivalent, and we hope that the new figures and descriptions in the previous revision make it clear where the difference lies, namely whether there is a category relationship between the three pitch contours making up each*

exemplar. (The contours themselves occur in both diagnostic and non-diagnostic contexts). Basic acoustic analyses would not show this difference, but the diagnostic and non-diagnostic bands differ on the basis of this regularity. Therefore it is important to show both that participants trained on a task where spectral band is not diagnostic do not show significant cross-band differences and also that participants trained on a task that does use this information show significantly greater concordance. This is what we demonstrate in the cross-group comparison of early alien identity+size experts compared to alien size experts, and what we additionally now show comparing the full (N=49) set of identity+size experts versus the size-trained group, with very similar results. (We appreciate R2's suggestion to add this new analysis).

In reflecting upon the reviewer's points, we realized we had failed to bring up the repetition suppression literature and its relevance to this question. We have revised our interim discussion of the acoustics, and added the following paragraphs and analyses to remedy this (pg 13 and pg 18 in the track-changes document that includes figures):

"The differential cortical activation across the high versus low frequency bands is consistent with an 'acquired salience' for category-diagnostic dimensions that reflects stable changes in cortical activation elicited by category exemplars that persist across changing task demands. Prioritization of category-diagnostic dimensions is present even in contexts that do not demand reliance on them. This effect is challenging to account for via a 'bottom-up' acoustic salience account, as all stimuli possessed complex information in each frequency band, in addition to amplitude variation. Results from the repetition suppression fMRI literature would generally suggest that the acoustic difference between the category diagnostic- and non-diagnostic bands - namely the higher-level regularity in pitch contour across the three individual tokens in the diagnostic band – would diminish activation in auditory regions sensitive to that information (Brown et al., 2013; Barron, Garvert, & Behrens, 2016, but cf. Saegert et al., 2013). To examine this question further, in the next two sections, we make more direct tests of the impact of alien identity category expertise on activation to the category-diagnostic band."

...

*"To test the generality of this result, we ran the same comparison between the Alien-Size-Trained cohort and the complete Alien Identity+Size cohort (N=49). As seen in **Fig S2**, we observe a similar difference in concordance maps, where the Alien-Identity+Size cohort shows significantly more concordance in most of the LH ROIs from the middle of the STG extending medially, as well as long the posterior STG. In the RH, the greater concordance in the full Alien Identity+Size cohort is again very similar to that observed in the comparison of Early Alien Identity+Size Experts versus Size experts, with greater concordance in multiple ROIs extending laterally to medially from the middle of the STG, as well as in anterior STG. This adds further support for the hypothesis that learning categories that are dependent upon statistically structured auditory information restricted to one frequency band drives the acquired salience of that structured information (Wang, Jendrichovsky, & Kanold, 2024), such that identity categorization experts (but not identity-naïve participants) exhibit frequency-band-specific recruitment of auditory cortex, even when categorizing stimuli along an orthogonal dimension (stimulus amplitude)."*

R2.4: Third, the post-training-only design cannot demonstrate that learning caused the neural

effect. The comparison between separately trained groups establishes an association with expertise, not within-person neural change.

AR-R2.4: *Throughout the manuscript, we have been careful to couch these findings in a way that does not imply deterministic proof for effects of training. However, we do provide multiple lines of evidence that groups who were trained on tasks where category identity was or was not carried within a spectral band differ in activation in the manner predicted by theory, and that degree of expertise modulates this effect. This experimental approach is logically equivalent to that taken in drug and interventional trials. That said, and like the reviewer, we are very interested in the process of representational change across learning and are planning new studies to investigate this. However, it was important to demonstrate this effect before moving on to such a study.*

R2.5: Moreover, identity and size blocks were interleaved, so attentional carryover remains a plausible explanation for the apparent persistence during size judgments. Claims that learning "drives" or produces stable attentional gain therefore remain too strong.

AR-R2.5: We addressed this in a full paragraph (below, last paragraph on pg 24) but have also gone through carefully and edited to make sure we do not make any unsupported claims.

"In future work, it will be important to show that the increased activation to identity-category-relevant frequency regions that is evident when they are not task-relevant (e.g. during size judgments) is not resulting from carry-over across experimental blocks. While we believe this is unlikely given the difficulty of the amplitude-conveyed alien size task (where weighting by spectrotemporal cues is counterproductive), it is possible that the activation patterns indicating acquired gain of the identity-diagnostic frequency band may diminish when not regularly reinforced via interleaving with blocks of the identity task."

R2.6: Finally, the group-comparison procedure requires fundamental justification. The authors convert mixed-model t values to z statistics and then apply a formula containing $1/(n-3)$. That variance formula is defined for Fisher-transformed correlation coefficients, not generally for z statistics derived from mixed models. Group differences should instead be tested directly through a concordance-by-group interaction in a single hierarchical model.

AR-R2.6: *We had discussed the z-statistic comparison approach with several statisticians who had not raised any concerns. However, we think the reviewer's approach is more straightforward, defensible, and interpretable, and have redone all group analyses using this method, with somewhat less widespread significance but extremely similar results. We thank the reviewer for the suggestion.*

R2.7: These issues require additional participants, substantial reanalysis, and stronger experimental controls rather than further clarification. I therefore cannot recommend publication.

AR-R2.6: *We have made a case for the sufficiency of the design to address the questions posed, addressed the question of power and participant N, and have carried out the suggested statistical reanalyses.*
