# Supplementary material for: Category learning drives cortical attentional gain to diagnostic dimensions of perceptual information
Source: PLoS Biol. 2026 Nov 12;24(11):e3004076. doi: 10.1371/journal.pbio.3004076 (PMC13759068; doi:10.1371/journal.pbio.3004076)
Supplement: Supplementary file 3 [file pbio.3004076.s007.pdf]

September 11th, 2026

Dear Dr. Schnell,

Thank you for your letter, and we are delighted to hear that our article will be acceptable for publication pending these final revisions. We would also like to thank you again for such a positive and constructive review experience.

Below are the four points you mention (Editor comment (EC) 1-4) , with confirmation that we have made these changes.

EC1: We would like to suggest a different title to improve its accessibility for our broad audience: "Category learning drives cortical attentional gain to diagnostic dimensions of perceptual information"

Response-EC1: *We have now changed the title, thank you for the suggestion.*

EC2: Please add the links to the funding agencies in the Financial Disclosure statement in the manuscript details.

Response-EC2: *We have inserted the current NIH and NSF links in the disclosure statement, and also removed funding information from the Acknowledgements section***.**

EC3: Please include the approval/license number of the ethical approval for the experiments.

Response-EC3: *Now included in Methods.*

EC4: Please include information in the Methods section whether the study has been conducted according to the principles expressed in the Declaration of Helsinki.

Response-EC4: *Now included in Methods.*
