# Supplementary material for: Category learning drives cortical attentional gain to diagnostic dimensions of perceptual information
Source: PLoS Biol. 2026 Nov 12;24(11):e3004076. doi: 10.1371/journal.pbio.3004076 (PMC13759068; doi:10.1371/journal.pbio.3004076)
Supplement: Peer review history

# Editor report: Decision Letter 0

**DOI:** 10.1371/journal.pbio.3004076.r001

**Authors:** Christian Schnell, PhD

16 Dec 2025

Dear Dr Dick,

Thank you for submitting your manuscript entitled "Category learning drives cortical attentional gain to diagnostic dimensions" for consideration as a Research Article by PLOS Biology.

Your manuscript has now been evaluated by the PLOS Biology editorial staff [as well as by an academic editor with relevant expertise and I am writing to let you know that we would like to send your submission out for external peer review. Please note that it is unlikely that we will be able to recruit the required reviewers before our Christmas break next week, so there will be some delays in the peer review process during the holiday period.

However, before we can send your manuscript to reviewers, we need you to complete your submission by providing the metadata that is required for full assessment. To this end, please login to Editorial Manager where you will find the paper in the 'Submissions Needing Revisions' folder on your homepage. Please click 'Revise Submission' from the Action Links and complete all additional questions in the submission questionnaire.

Once your full submission is complete, your paper will undergo a series of checks in preparation for peer review. After your manuscript has passed the checks it will be sent out for review. To provide the metadata for your submission, please Login to Editorial Manager (https://www.editorialmanager.com/pbiology) within two working days, i.e. by Dec 18 2025 11:59PM.

If your manuscript has been previously peer-reviewed at another journal, PLOS Biology is willing to work with those reviews in order to avoid re-starting the process. Submission of the previous reviews is entirely optional and our ability to use them effectively will depend on the willingness of the previous journal to confirm the content of the reports and share the reviewer identities. Please note that we reserve the right to invite additional reviewers if we consider that additional/independent reviewers are needed, although we aim to avoid this as far as possible. In our experience, working with previous reviews does save time.

If you would like us to consider previous reviewer reports, please edit your cover letter to let us know and include the name of the journal where the work was previously considered and the manuscript ID it was given. In addition, please upload a response to the reviews as a 'Prior Peer Review' file type, which should include the reports in full and a point-by-point reply detailing how you have or plan to address the reviewers' concerns.

During the process of completing your manuscript submission, you will be invited to opt-in to posting your pre-review manuscript as a bioRxiv preprint. Visit http://journals.plos.org/plosbiology/s/preprints for full details. If you consent to posting your current manuscript as a preprint, please upload a single Preprint PDF.

Feel free to email us at plosbiology@plos.org if you have any queries relating to your submission.

Kind regards,

Christian

Christian Schnell, PhD

Senior Editor

PLOS Biology

cschnell@plos.org

# Peer review: Decision Letter 1

**DOI:** 10.1371/journal.pbio.3004076.r002

**Authors:** Christian Schnell, PhD

24 Feb 2026

Dear Fred,

Thank you for your patience while your manuscript "Category learning drives cortical attentional gain to diagnostic dimensions" was peer-reviewed at PLOS Biology. It has now been evaluated by the PLOS Biology editors, an Academic Editor with relevant expertise, and by several independent reviewers.

In light of the reviews, which you will find at the end of this email, we would like to invite you to revise the work to thoroughly address the reviewers' reports.

As you will see below, the reviewers agree that your study is interesting, but they also list a number of concerns about potential confounders, the lack of pre-training data, missing analyses and the lack of clarity.

Given the extent of revision needed, we cannot make a decision about publication until we have seen the revised manuscript and your response to the reviewers' comments. Your revised manuscript is likely to be sent for further evaluation by all or a subset of the reviewers.

In addition to these revisions, you will need to complete some formatting changes, which you will receive in a follow up email. A member of our team will be in touch with a set of requests shortly.

We expect to receive your revised manuscript within 3 months. Please email us (plosbiology@plos.org) if you have any questions or concerns, or would like to request an extension.

At this stage, your manuscript remains formally under active consideration at our journal; please notify us by email if you do not intend to submit a revision so that we may withdraw it.

**IMPORTANT - SUBMITTING YOUR REVISION**

Your revisions should address the specific points made by each reviewer. Please submit the following files along with your revised manuscript:

1. A 'Response to Reviewers' file - this should detail your responses to the editorial requests, present a point-by-point response to all of the reviewers' comments, and indicate the changes made to the manuscript.

*NOTE: In your point-by-point response to the reviewers, please provide the full context of each review. Do not selectively quote paragraphs or sentences to reply to. The entire set of reviewer comments should be present in full and each specific point should be responded to individually, point by point.

You should also cite any additional relevant literature that has been published since the original submission and mention any additional citations in your response.

2. In addition to a clean copy of the manuscript, please also upload a 'track-changes' version of your manuscript that specifies the edits made. This should be uploaded as a "Revised Article with Changes Highlighted" file type.

*Re-submission Checklist*

When you are ready to resubmit your revised manuscript, please refer to this re-submission checklist: https://plos.io/Biology_Checklist

To submit a revised version of your manuscript, please go to https://www.editorialmanager.com/pbiology/ and log in as an Author. Click the link labelled 'Submissions Needing Revision' where you will find your submission record.

Please make sure to read the following important policies and guidelines while preparing your revision:

*Published Peer Review*

Please note while forming your response, if your article is accepted, you may have the opportunity to make the peer review history publicly available. The record will include editor decision letters (with reviews) and your responses to reviewer comments. If eligible, we will contact you to opt in or out. Please see here for more details:

https://blogs.plos.org/plos/2019/05/plos-journals-now-open-for-published-peer-review/

*PLOS Data Policy*

Please note that as a condition of publication PLOS' data policy (http://journals.plos.org/plosbiology/s/data-availability) requires that you make available all data used to draw the conclusions arrived at in your manuscript. If you have not already done so, you must include any data used in your manuscript either in appropriate repositories, within the body of the manuscript, or as supporting information (N.B. this includes any numerical values that were used to generate graphs, histograms etc.). For an example see here: http://www.plosbiology.org/article/info%3Adoi%2F10.1371%2Fjournal.pbio.1001908#s5

*Blot and Gel Data Policy*

We require the original, uncropped and minimally adjusted images supporting all blot and gel results reported in an article's figures or Supporting Information files. We will require these files before a manuscript can be accepted so please prepare them now, if you have not already uploaded them. Please carefully read our guidelines for how to prepare and upload this data: https://journals.plos.org/plosbiology/s/figures#loc-blot-and-gel-reporting-requirements

*Protocols deposition*

To enhance the reproducibility of your results, we recommend that if applicable you deposit your laboratory protocols in protocols.io, where a protocol can be assigned its own identifier (DOI) such that it can be cited independently in the future. Additionally, PLOS ONE offers an option for publishing peer-reviewed Lab Protocol articles, which describe protocols hosted on protocols.io. Read more information on sharing protocols at https://plos.org/protocols?utm_medium=editorial-email&utm_source=authorletters&utm_campaign=protocols

Thank you again for your submission to our journal. We hope that our editorial process has been constructive thus far, and we welcome your feedback at any time. Please don't hesitate to contact us if you have any questions or comments.

Sincerely,

Christian

Christian Schnell, PhD

Senior Editor

PLOS Biology

cschnell@plos.org

------------------------------------

REVIEWS:

Reviewer #1 (Chris Petkov signed his report): I apologize for my delayed review. Below, I follow a structured peer-review format in the layout of my review report. As you'll see, I have more to say about study strengths than what could be improved to strengthen the manuscript (only a handful of relatively minor suggestions).

Summary: This is a tour-de-force study studying the interface of category learning and attentional gain in the auditory system. The authors conduct a well powered (n = 69) an category learning study with 5 days of training prior to fMRI. Two different groups of participants take part in either the alien identity and size task or just the size task; size of the alien selected on the screen is dependent on the amplitude of the sounds heard, and identity is related to a combination of frequency and spectro-temporal features that the participant extracts from a large set of exemplars. After five days of training the participants are scanned with fMRI at 3T and the results contrasting the category (identity and/or size) information with concordance maps determined from tonotopic mapping individually but transformed to a common space and attention to tonotopy task effects. The results demonstrate that category learning harnesses mechanisms more commonly attributed to selective attention effects, and that even though there are regularities in the sounds, the effects related to attention are strongest when the category information is task relevant. The results have strong implications for understanding expert learning, and may be relevant for developing further to diagnose learning related difficulties in combination with behavior and neuroimaging, although this could be better developed in the discussion.

Study strengths:

There are several strengths of this study, including the large sample sizes and dedication to multi-day training prior to fMRI. The strength of the large sample sizes mean that the research team can do sub-group comparisons of identity and size with size learning groups as well as early versus late experts or high-low achievers. The benefit of the task being non-linguistic is that the sounds and aliens are not readily verbalizable to reduce language-related confounds. The fMRI activity maps are registered to the subject's specific tonotopic map. The size task is not trivial and the authors have done a tremendous job matching task difficulty between the identity and size task performance for the scanning session, to rule out task difficulty as a potential confound. Moreover, the study is very well written and the narrative with figures and results elegantly relayed to the reader with few unaddressed questioned within the relevant sections.

Main suggestions:

1)Auditory cortex labels and quantification would be important to see: To auditory cortex non-experts, it would be important to add labels to identify the subregions of auditory cortex. More importantly, it would be useful to quantify the effects attributed to STG, HG or other subregions of auditory cortex in the discussion.

2)Brain wide effects underspecified: I understand the importance of focusing on effects over tonotopic cortex altered by category learning and influenced by selective attention, but it would be useful to have at least one main manuscript figure that shows the brain wide effects of identity and size category comparisons.

3)Basis for categorization learning is unclear: I am pleasantly surprised that the high/low categorization was not as easy to learn as I would have thought (e.g., mentally draw a category boundary between the low and high frequency space - done deal). Therefore, it seems the identity categorization relied also on the spectro-temporal features within the high/low categories. It would be useful to at least comment in the discussion on how the category learning also relied on features within the high/low frequency space that were diagnostic for the alien identity. I didn't think memorizing the alien identities would help since there were so many exemplars, but as you can tell I was a little confused on the basis for the alien identity learning besides the high/low frequency dimension that seems trivial to learn. Related to this, I wasn't clear how amplitude size was manipulated to ensure the task was not overly easy for participants (needing a little more clarity than I found in the methods).

4)Broader impact discussion could be useful: The discussion is interesting in relating to the relevant literature, but I thought there was a missed opportunity to couch the observations in the utility of either the approach or the observations for diagnosing or ameliorating learning difficulties. Certainly there is interesting discussion on the non-attended category information not eliciting as strong of a modulation of auditory cortex as one might expect given statistical learning effects can be evident in the brain. But I wondered whether the current approach and results, such as those relating to statistical learning, could be further developed to understand auditory expertise (early or late learners) and those with language and/or learning difficulties, which of course are outside of the scope of this study but might benefit from some discussion with regards to the potential future impact of this work.

Minor suggestions:

5)In figure 4 I wasn't sure if Tonotopy 'vs.' Alien Identity/Size, was really 'versus' or a concordance of tonotopy and alien size, which is what I think you mean to relay with the labels.

6)I was curious about the prefrontal involvement in the task, but have already commented on the brain wide results that would be useful for readers interested in seeing what is happening outside of auditory cortex. It would be good to have some discussion of these results if the authors deem them important to include in their study.

I again apologize for the delay in completing my review, and I hope the authors and editors find the structured review useful, with only a few eminently addressable suggestions for improving an already strong manuscript.

-Chris Petkov

Reviewer #2: This study investigates the neural mechanisms underlying auditory category learning, specifically examining how the auditory cortex adapts to diagnostic acoustic features. The study employed a novel "Alien" soundscape paradigm in which participants were trained to categorize complex sounds based on spectrotemporal patterns in specific frequency bands (High vs. Low). Using a "Concordance" metric that correlates voxel-wise frequency preference (tonotopy) with task-evoked activation, the study aims to determine whether learning selectively amplifies gain in category-relevant frequency channels. The primary finding is that category learning sharpens neural tuning: voxels preferring a specific frequency band show increased activity when that band contains diagnostic information. Furthermore, the authors report that this enhanced sensitivity persists even when the diagnostic dimension is irrelevant to the immediate task (i.e., during a "Size" judgment task), suggesting a stable, plasticity-driven change in cortical representation rather than transient attentional modulation. Overall, I like the study ideas, and the study shows promising evidence in support of the interconnection. However, potential confounders or a lack of clarity may undermine the robustness of the evidence.

1) My primary concern relates to the structural similarity between the localizer contrasts and the experimental contrasts, and whether the observed "concordance" is truly an effect of category learning or a result of shared acoustic sensitivities. The tonotopy localizer contrasts High vs. Low frequencies. Similarly, the category training contrast compares High-Diagnostic vs. Low-Diagnostic trials. Consequently, the "Concordance" metric essentially measures the correlation between these two very similar contrasts.

While the authors attribute this overlap to learned attention, there is a risk that this is driven by shared sensitivity to frequency modulation (FM) or spectrotemporal complexity rather than tonotopic reorganization. If the diagnostic patterns contain specific FM sweeps that drive neurons in the corresponding frequency regions, the correlation could be a bottom-up acoustic effect. The authors need to rigorously demonstrate that the concordance is not simply due to the fact that the "High-Diagnostic" stimuli physically contain more acoustic energy or FM complexity in the high band, which would naturally correlate with a high-frequency tonotopy map regardless of learning.

2) The study relies exclusively on post-training fMRI data. While the comparison between the "Identity-trained" and "Size-trained" (control) groups allows for between-subject inferences, the lack of a pre-training baseline makes it difficult to definitively claim that the observed effects represent plasticity or a change in organization. Without pre-training data, inherent differences in auditory cortical organization might have influenced results. A longitudinal design (Pre- vs. Post-scan) could have tracked this emergence within individuals. The authors should more transparently discuss this limitation and justify why a cross-sectional approach supports claims of cortex "reshaping'.

3) The manuscript includes analyses regarding individual differences, specifically splitting participants into subgroups (e.g., based on learning performance or strategy) to correlate behavior with neural concordance. Given the likely modest total sample size (typical of fMRI studies), subdividing the cohort raises significant concerns about statistical power. When the sample is split, the N for each subgroup is likely small. Correlations from small samples are unstable and influenced by outliers. The authors should provide scatterplots to confirm robustness. If subgroup N falls below thresholds for stable correlations, interpret those analyses cautiously or move them to supplementary materials as exploratory.

Minor Concerns

Logic Visualization: The logic underpinning the "Concordance" analysis and the relationships among the three tasks (Categorization, Tonotopy, Attention-to-Tonotopy) are complex. It is somewhat difficult to follow the progression of the analysis in the text alone. I strongly recommend adding a schematic figure or flowchart that visually depicts: How the maps were generated for each task. How the "Concordance" value is mathematically derived from the overlap of these maps. The specific hypothesis for the Experimental vs. Control group within this schematic.

ROI Definition: Please clarify exactly how the Regions of Interest (ROIs) were defined. Were they defined anatomically or functionally? If functional, were the data used to define the ROI independent of the data used for the main analysis to avoid circularity (double-dipping)?

Acoustic Controls: In the "Size" task, were the stimuli acoustically identical between the "Big" and "Small" categories aside from amplitude? It would be helpful to see a brief analysis confirming that the High and Low frequency bands were energetically balanced across the different conditions to ensure no low-level acoustic confounds influenced the "Size" task neural data.

Reviewer #3 (Patrik Wikman signed his report): The manuscript studies how learned category membership, driven by frequency specific diagnostic information, affects the auditory cortex representation of the learned categories. One participant group learned that spectral attributes in one of two frequency channels (high vs. low) differentiated novel sound objects called "space aliens", whole the other frequency range did not. Participants, thus, learned by experience driven learning in a 2ACF space alien identity task to pay attention to either high frequency spectral attributes or low spectral attributes of the objects. In addition the same participants performed a space alien size task to evaluate the size of the alien based on amplitude. In the size task the identity of the alien was irrelevant.

During fMRI tonotopic gradients of auditory cortex parcels were evaluated using two tasks, passive frequency selectivity and attention-driven frequency selectivity. The same participants also performed the 2ACF task in the scanner. The main result of the study is that the frequency selectivity during the space alien identity classification was predicted by the parcels frequency selectivity probed both with the passive and active tonotopy task. This effect was found both in the identity task, where the information was task relevant, and in the size task, where it was irrelevant. Furthermore, the level of this correlation depended on the achieved expertise and the learning rate of the participant. Importantly, similar findings were not found in another group of participants that had not learned to pay attention to the high vs. low frequency bands that contained the diagnostic information about identity. Thus, the authors argue that an important facet of category learning is to learn to pay attention to the category differentiating low-level features and that this produces task-dependent and plastic responsiveness in the auditory cortex.

My assessment is that the results are interesting and novel. However, the design and analysis methods are complex and consequently the authors could help the reader with adding in-text citations throughout the manuscript (e.g., referring to the relevant chapter in the methodology in the results). Below I have outlined my specific suggestions:

1. The tasks, training days, tasks in scanner of the groups etc. are scattered through the Methods and Results text, a flow chart in the beginning of the Results would help the reader to understand the complex design.

2. Figure 3 is key to understanding the analysis approach. However, I find the figure difficult to follow. I understand that the task-concordance analyses have been used in the groups prior work, but since it is a fairly novel approach I would make it clearer here. Especially I did not understand how many category-betas per participant were extracted and how many tonotopy-betas were extracted and how they were paired. A vector image showing how the regression was conducted would help in understanding this, you can use RSA studies as a template for this. Note also that since the directed attention task has not been introduced in the text the author cannot understand what you are referring to. Thus, I would add a in-text reference and introduce all tasks in the beginning of the results (see also comment 8).

3. Page 3. please define what you mean by endogenous attention. I would also contrast it with exogenous attention, as you suggest that after learning the frequency information may cause learned salience. Regarding such effects you could also discuss prior studies that have shown similar experience driven salience effects (see e.g., Addleman, Douglas A

Jiang, Yuhong V, 2019, Experience-driven auditory attention, Trends in Cognitive Sciences)

4. Page 4. The space alien identity and space alien size differentiation was not very clear while reading the Intro. First I thought space referred to spatial information, not space as a concept. Please add a couple of sentences explaining what is meant by these so that the reader understands the idea from the start.

5.In the figures (e.g., Fig 4) you use the abbreviation vs. (versus) but if I understood correctly you mean that something has been used as an regressor for something, please change this as it is misleading (one thinks its a contrast between tasks).

6. Page 9. Could you explain what the concordance between the identity and size tasks was supposed to indicate, currently I do not understand the added value of this regression analysis.

7.Page 11. Group 1 and Group 2 have different levels of exposure to the space aliens, thus, could it be that some of the discrepancies in the neural effects between the groups are explained by exposure. I would argue that the fact that task achievement modulated the correspondence in group 1 (fig 5) argues against this, but could be good to mention this in the limitations.

8. Page 12. The description of the attention driving task should briefly be outlined in the beginning of the results, now its a bit confusing see also comment 1.

9. Could you discuss briefly in the discussion why concordance in the tonotopy-identity, tonotopy-size, and tonotpopy-attention driven as well as attention driven-identity yield different patterns of results, do they bear any theoretical significance, now in the discussion the analyses are described as yielding fairly similar patterns.

10. Page 20. Please indicate whether the exemplars used during the fMRI experiment were novel or had been used in training and whether the exemplars for the size experiment were different from the ones in the identity experiment.

11. Page 20: "To facilitate comparisons between the alien identity and alien size categorization blocks, 20 of

the 40 blocks that appeared during the alien identity task also appeared in the alien size task,

with the same fixed trial order within the block." Can you explain how this facilitates comparison.

Reviewer #4: This project addresses the important issue of auditory cortical processing correlates of acoustic category learning. Strengths include a large sample size, at least overall, strong and clear behavioral evidence of category learning. This is a rich data set, and well-worth publishing. In general, it is a good match to PLOS 1, and provides useful information to the field. Some weaknesses are described below.

Major points

1.A major weakness is not having longitudinal measures of fMRI (baseline before training as well as post-training). This limits inferences about category learning-related changes in activation responses. The use of sub-groups is helpful, by dividing according to coarse differences in learning across days, but this is also only reflected in the one fMRI session after training.

2.I could not find a statistical analysis of the sub-group activation patterns, potentially due to low power. This limits inferences on how activations relate to behavior.

3.The main analyses used the concordance measure, which is a difference score. The Authors should more clearly consider other differences in stimuli besides frequency bands of interest (diagnostic vs. non-diagnostic) that may influence the concordance measure (e.g. amplitude modulation and frequency modulations). This issue probably receives some control by having categories based on high- or low-frequency information, but does not rule out other stimulus features that may covary with category. The Authors asserted that "The differential cortical activation across the high versus low frequency bands cannot easily be attributed to 'bottom-up' acoustic salience, as all stimuli possessed information in each frequency band in addition to amplitude variation." (p. 9). This may be true, but the burden of proof is on the Authors to make a more compelling argument. Having information in different frequency bands vs. not having information that covaries with category are two different ideas.

4.In general, the Discussion section needs to scrutinize the current results, including the above weaknesses. In its current form, the Discussion quickly moves to covering the research of others, without carefully considering what was learned from the present study.

5.The Authors may want to comment further on hemispheric differences, perhaps with quantitative comparison. There may be a connection with the diagnostic stimulus features being variations of F0 Mandarin chirps and left vs. right hemisphere auditory processing for segmental and suprasegmental information, respectively.

# Author comment: Author response to Decision Letter 2

**DOI:** 10.1371/journal.pbio.3004076.r003

17 Jun 2026

Attachment Submitted filename: ResponseLetter-SpaceNarhwhalsPLoSBio-R1-260615-FINAL.pdf

# Peer review: Decision Letter 2

**DOI:** 10.1371/journal.pbio.3004076.r004

**Authors:** Christian Schnell, PhD

22 Jul 2026

Dear Dr Dick,

Thank you for your patience while we considered your revised manuscript "Category learning drives cortical attentional gain to diagnostic dimensions" for consideration as a Research Article at PLOS Biology. Your revised study has now been evaluated by the PLOS Biology editors, the Academic Editor and three of the original reviewers.

In light of the reviews, which you will find at the end of this email, we are pleased to offer you the opportunity to address the remaining points from the reviewers, in particular Reviewer 2 via a thorough discussion of the concerns raised by Reviewer 2 in the discussion section of the manuscript if you are unable to address them experimentally. We will then assess your revised manuscript and your response to the reviewers' comments with our Academic Editor aiming to avoid further rounds of peer-review, although we might need to consult with the reviewers, depending on the nature of the revisions.

In addition to these revisions, you may need to complete some formatting changes, which you will receive in a follow up email. A member of our team will be in touch with a set of requests shortly. If you do not receive a separate email within a few days, please assume that checks have been completed, and no additional changes are required.

We expect to receive your revised manuscript within 1 month. Please email us (plosbiology@plos.org) if you have any questions or concerns, or would like to request an extension.

At this stage, your manuscript remains formally under active consideration at our journal; please notify us by email if you do not intend to submit a revision so that we withdraw the manuscript.

**IMPORTANT - SUBMITTING YOUR REVISION**

Your revisions should address the specific points made by each reviewer. Please submit the following files along with your revised manuscript:

1. A 'Response to Reviewers' file - this should detail your responses to the editorial requests, present a point-by-point response to all of the reviewers' comments, and indicate the changes made to the manuscript.

*NOTE: In your point-by-point response to the reviewers, please provide the full context of each review. Do not selectively quote paragraphs or sentences to reply to. The entire set of reviewer comments should be present in full and each specific point should be responded to individually.

You should also cite any additional relevant literature that has been published since the original submission and mention any additional citations in your response.

2. In addition to a clean copy of the manuscript, please also upload a 'track-changes' version of your manuscript that specifies the edits made. This should be uploaded as a "Revised Article with Changes Highlighted " file type.

*Resubmission Checklist*

When you are ready to resubmit your revised manuscript, please refer to this resubmission checklist: https://plos.io/Biology_Checklist

To submit a revised version of your manuscript, please go to https://www.editorialmanager.com/pbiology/ and log in as an Author. Click the link labelled 'Submissions Needing Revision' where you will find your submission record.

Please make sure to read the following important policies and guidelines while preparing your revision:

*Published Peer Review*

Please note while forming your response, if your article is accepted, you may have the opportunity to make the peer review history publicly available. The record will include editor decision letters (with reviews) and your responses to reviewer comments. If eligible, we will contact you to opt in or out. Please see here for more details:

https://blogs.plos.org/plos/2019/05/plos-journals-now-open-for-published-peer-review/

*PLOS Data Policy*

Please note that as a condition of publication PLOS' data policy (http://journals.plos.org/plosbiology/s/data-availability) requires that you make available all data used to draw the conclusions arrived at in your manuscript. If you have not already done so, you must include any data used in your manuscript either in appropriate repositories, within the body of the manuscript, or as supporting information (N.B. this includes any numerical values that were used to generate graphs, histograms etc.). For an example see here: http://www.plosbiology.org/article/info%3Adoi%2F10.1371%2Fjournal.pbio.1001908#s5

*Blot and Gel Data Policy*

We require the original, uncropped and minimally adjusted images supporting all blot and gel results reported in an article's figures or Supporting Information files. We will require these files before a manuscript can be accepted so please prepare them now, if you have not already uploaded them. Please carefully read our guidelines for how to prepare and upload this data: https://journals.plos.org/plosbiology/s/figures#loc-blot-and-gel-reporting-requirements

*Protocols deposition*

To enhance the reproducibility of your results, we recommend that if applicable you deposit your laboratory protocols in protocols.io, where a protocol can be assigned its own identifier (DOI) such that it can be cited independently in the future. Additionally, PLOS ONE offers an option for publishing peer-reviewed Lab Protocol articles, which describe protocols hosted on protocols.io. Read more information on sharing protocols at https://plos.org/protocols?utm_medium=editorial-email&utm_source=authorletters&utm_campaign=protocols

Thank you again for your submission to our journal. We hope that our editorial process has been constructive thus far, and we welcome your feedback at any time. Please don't hesitate to contact us if you have any questions or comments.

Sincerely,

Christian

Christian Schnell, PhD

Senior Editor

PLOS Biology

cschnell@plos.org

----------------------------------------------------------------

REVIEWS:

Reviewer #2: I appreciate the authors' efforts to clarify the design and analyses. However, my central concerns remain unresolved and affect the main conclusions.

First, the learning-trajectory analyses remain underpowered. Dividing participants into Early/Late Experts or High/Low Achievers is an individual-differences analysis because the claim is that neural concordance varies with learning trajectory. These comparisons contain only 16-17 participants per subgroup, and the High/Low analysis discards the middle tercile. Many voxelwise observations cannot compensate for the small number of independent participants. Small task-fMRI samples yield unstable group effects, and categorizing continuous variables further reduces information and power.

Second, the acoustic alternative has not been excluded. The diagnostic band contains chirps from the same category, whereas the nondiagnostic band combines chirps across categories; these conditions therefore differ in spectrotemporal regularity and coherence. Explaining that the chirps came from the same overall pool is insufficient without quantitative acoustic controls. The null result in the smaller control group is not evidence of acoustic equivalence.

Third, the post-training-only design cannot demonstrate that learning caused the neural effect. The comparison between separately trained groups establishes an association with expertise, not within-person neural change. Moreover, identity and size blocks were interleaved, so attentional carryover remains a plausible explanation for the apparent persistence during size judgments. Claims that learning "drives" or produces stable attentional gain therefore remain too strong.

Finally, the group-comparison procedure requires fundamental justification. The authors convert mixed-model t values to z statistics and then apply a formula containing 1/(n−3). That variance formula is defined for Fisher-transformed correlation coefficients, not generally for z statistics derived from mixed models. Group differences should instead be tested directly through a concordance-by-group interaction in a single hierarchical model.

These issues require additional participants, substantial reanalysis, and stronger experimental controls rather than further clarification. I therefore cannot recommend publication.

Reviewer #3 (Patrik Wikman signed his report): I want to thank the authors for the significant amount of work they put into clarifying the manuscript. I feel that especially the changes to Figure 3 and the new figure 1 makes it much easier to understand the setup.

Reviewer #4: The authors did a good job with revisions. No additional comments

# Author comment: Author response to Decision Letter 3

**DOI:** 10.1371/journal.pbio.3004076.r005

3 Sep 2026

Attachment Submitted filename: PloSBioR2-response-FINAL.pdf

# Editor report: Decision Letter 3

**DOI:** 10.1371/journal.pbio.3004076.r006

**Authors:** Christian Schnell, PhD

11 Sep 2026

Dear Dr Dick,

Thank you for your patience while we considered your revised manuscript "Category learning drives cortical attentional gain to diagnostic dimensions" for publication as a Research Article at PLOS Biology. This revised version of your manuscript has been evaluated by the PLOS Biology editors and the Academic Editor.

Based on our Academic Editor's assessment of your revision, we are likely to accept this manuscript for publication, provided you satisfactorily address the following data and other policy-related requests:

* We would like to suggest a different title to improve its accessibility for our broad audience: "Category learning drives cortical attentional gain to diagnostic dimensions of perceptual information"

* Please add the links to the funding agencies in the Financial Disclosure statement in the manuscript details.

* Please include the approval/license number of the ethical approval for the experiments.

* Please include information in the Methods section whether the study has been conducted according to the principles expressed in the Declaration of Helsinki.

* DATA POLICY:

You may be aware of the PLOS Data Policy, which requires that all data be made available without restriction: http://journals.plos.org/plosbiology/s/data-availability. For more information, please also see this editorial: http://dx.doi.org/10.1371/journal.pbio.1001797

Note that we do not require all raw data. Rather, we ask that all individual quantitative observations that underlie the data summarized in the figures and results of your paper be made available in one of the following forms:

1) Supplementary files (e.g., excel). Please ensure that all data files are uploaded as 'Supporting Information' and are invariably referred to (in the manuscript, figure legends, and the Description field when uploading your files) using the following format verbatim: S1 Data, S2 Data, etc. Multiple panels of a single or even several figures can be included as multiple sheets in one excel file that is saved using exactly the following convention: S1_Data.xlsx (using an underscore).

2) Deposition in a publicly available repository. Please also provide the accession code or a reviewer link so that we may view your data before publication.

Regardless of the method selected, please ensure that you provide the individual numerical values that underlie the summary data displayed in the following figure panels as they are essential for readers to assess your analysis and to reproduce it: 3A and 6A

NOTE: the numerical data provided should include all replicates AND the way in which the plotted mean and errors were derived (it should not present only the mean/average values).

Please also ensure that figure legends in your manuscript include information on where the underlying data can be found, and ensure your supplemental data file/s has a legend.

Please ensure that your Data Statement in the submission system accurately describes where your data can be found.

* CODE POLICY

Per journal policy, if you have generated any custom code during the course of this investigation, please make it available without restrictions. Please ensure that the code is sufficiently well documented and reusable, and that your Data Statement in the Editorial Manager submission system accurately describes where your code can be found. More information on our Code Policy, what and how to share can be found here: https://journals.plos.org/plosbiology/s/code-availability

Please note that we cannot accept sole deposition of code in GitHub, as this could be changed after publication. However, you can archive this version of your publicly available GitHub code to Zenodo. Once you do this, it will generate a DOI number, which you will need to provide in the Data Accessibility Statement (you are welcome to also provide the GitHub access information). See the process for doing this here: https://docs.github.com/en/repositories/archiving-a-github-repository/referencing-and-citing-content

As you address these items, please take this last chance to review your reference list to ensure that it is complete and correct. If you have cited papers that have been retracted, please include the rationale for doing so in the manuscript text, or remove these references and replace them with relevant current references. Any changes to the reference list should be mentioned in the cover letter that accompanies your revised manuscript.

In addition to these revisions, you may need to complete some formatting changes, which you will receive in a follow up email. A member of our team will be in touch with a set of requests shortly. If you do not receive a separate email within a few days, please assume that checks have been completed, and no additional changes are required.

We expect to receive your revised manuscript within two weeks.

To submit your revision, please go to https://www.editorialmanager.com/pbiology/ and log in as an Author. Click the link labelled 'Submissions Needing Revision' to find your submission record. Your revised submission must include the following:

- a cover letter that should detail your responses to any editorial requests, if applicable, and whether changes have been made to the reference list

- a Response to Reviewers file that provides a detailed response to the reviewers' comments (if applicable, if not applicable please do not delete your existing 'Response to Reviewers' file.)

- a track-changes file indicating any changes that you have made to the manuscript.

NOTE: If Supporting Information files are included with your article, note that these are not copyedited and will be published as they are submitted. Please ensure that these files are legible and of high quality (at least 300 dpi) in an easily accessible file format. For this reason, please be aware that any references listed in an SI file will not be indexed. For more information, see our Supporting Information guidelines:

https://journals.plos.org/plosbiology/s/supporting-information

*Published Peer Review History*

Please note that you may have the opportunity to make the peer review history publicly available. The record will include editor decision letters (with reviews) and your responses to reviewer comments. If eligible, we will contact you to opt in or out. Please see here for more details:

https://plos.org/published-peer-review-history/

*Press*

Should you, your institution's press office or the journal office choose to press release your paper, please ensure you have opted out of Early Article Posting on the submission form. We ask that you notify us as soon as possible if you or your institution is planning to press release the article.

*Protocols deposition*

To enhance the reproducibility of your results, we recommend that if applicable you deposit your laboratory protocols in protocols.io, where a protocol can be assigned its own identifier (DOI) such that it can be cited independently in the future. Additionally, PLOS ONE offers an option for publishing peer-reviewed Lab Protocol articles, which describe protocols hosted on protocols.io. Read more information on sharing protocols at https://plos.org/protocols?utm_medium=editorial-email&utm_source=authorletters&utm_campaign=protocols

Please do not hesitate to contact me should you have any questions.

Sincerely,

Christian

Christian Schnell, PhD

Senior Editor

cschnell@plos.org

PLOS Biology

# Author comment: Author response to Decision Letter 4

**DOI:** 10.1371/journal.pbio.3004076.r007

12 Sep 2026

Attachment Submitted filename: PloSBio_R3-response-FINAL.pdf

# Editor report: Decision Letter 4

**DOI:** 10.1371/journal.pbio.3004076.r008

**Authors:** Christian Schnell, PhD

2 Oct 2026

Dear Fred,

Thank you for your patience while we considered your revised manuscript "Category learning drives cortical attentional gain to diagnostic dimensions of perceptual information" for publication as a Research Article at PLOS Biology. As discussed, this revised version of your manuscript has been evaluated by the PLOS Biology editors.

Thank you for addressing most the editorial request. As discussed, there are a few items that still need addressing. Because I will be travelling over the next week, I am sending the manuscript back to you, so you can address the remaining points and resubmit your manuscript when you are done.

* Thank you for providing the DOI for your code and minimal dataset.

* As mentioned, please also provide the source data. These files can be either uploaded into the repository or provided as a supplementary file. The source data are the individual numerical values that underlie the summary data displayed in the following figure panels as they are essential for readers to assess your analysis and to reproduce it: 3A and 6A

The numerical data provided should include all replicates AND the way in which the plotted mean and errors were derived (it should not present only the mean/average values).

* Please also ensure that figure legends in your manuscript include information on where the underlying data can be found, and ensure your supplemental data file/s has a legend.

As you address these items, please take this last chance to review your reference list to ensure that it is complete and correct. If you have cited papers that have been retracted, please include the rationale for doing so in the manuscript text, or remove these references and replace them with relevant current references. Any changes to the reference list should be mentioned in the cover letter that accompanies your revised manuscript.

In addition to these revisions, you may need to complete some formatting changes, which you will receive in a follow up email. A member of our team will be in touch with a set of requests shortly. If you do not receive a separate email within a few days, please assume that checks have been completed, and no additional changes are required.

We expect to receive your revised manuscript within two weeks.

To submit your revision, please go to https://www.editorialmanager.com/pbiology/ and log in as an Author. Click the link labelled 'Submissions Needing Revision' to find your submission record. Your revised submission must include the following:

- a cover letter that should detail your responses to any editorial requests, if applicable, and whether changes have been made to the reference list

- a Response to Reviewers file that provides a detailed response to the reviewers' comments (if applicable, if not applicable please do not delete your existing 'Response to Reviewers' file.)

- a track-changes file indicating any changes that you have made to the manuscript.

NOTE: If Supporting Information files are included with your article, note that these are not copyedited and will be published as they are submitted. Please ensure that these files are legible and of high quality (at least 300 dpi) in an easily accessible file format. For this reason, please be aware that any references listed in an SI file will not be indexed. For more information, see our Supporting Information guidelines:

https://journals.plos.org/plosbiology/s/supporting-information

*Published Peer Review History*

Please note that you may have the opportunity to make the peer review history publicly available. The record will include editor decision letters (with reviews) and your responses to reviewer comments. If eligible, we will contact you to opt in or out. Please see here for more details:

https://plos.org/published-peer-review-history/

*Press*

Should you, your institution's press office or the journal office choose to press release your paper, please ensure you have opted out of Early Article Posting on the submission form. We ask that you notify us as soon as possible if you or your institution is planning to press release the article.

*Protocols deposition*

To enhance the reproducibility of your results, we recommend that if applicable you deposit your laboratory protocols in protocols.io, where a protocol can be assigned its own identifier (DOI) such that it can be cited independently in the future. Additionally, PLOS ONE offers an option for publishing peer-reviewed Lab Protocol articles, which describe protocols hosted on protocols.io. Read more information on sharing protocols at https://plos.org/protocols?utm_medium=editorial-email&utm_source=authorletters&utm_campaign=protocols

Please do not hesitate to contact me should you have any questions.

Sincerely,

Christian

Christian Schnell, PhD

Senior Editor

cschnell@plos.org

PLOS Biology

# Editor report: Decision Letter 5

**DOI:** 10.1371/journal.pbio.3004076.r009

**Authors:** Christian Schnell, PhD

12 Oct 2026

Dear Fred,

Thank you for the submission of your revised Research Article "Category learning drives cortical attentional gain to diagnostic dimensions of perceptual information" for publication in PLOS Biology. On behalf of my colleagues and the Academic Editor, Manuel Malmierca, I am pleased to say that we can in principle accept your manuscript for publication, provided you address any remaining formatting and reporting issues. These will be detailed in an email you should receive within 2-3 business days from our colleagues in the journal operations team; no action is required from you until then. Please note that we will not be able to formally accept your manuscript and schedule it for publication until you have completed any requested changes.

Please take a minute to log into Editorial Manager at http://www.editorialmanager.com/pbiology/, click the "Update My Information" link at the top of the page, and update your user information to ensure an efficient production process.

PRESS

We frequently collaborate with press offices. If your institution or institutions have a press office, please notify them about your upcoming paper at this point, to enable them to help maximize its impact. If the press office is planning to promote your findings, we would be grateful if they could coordinate with biologypress@plos.org. If you have previously opted in to the early version process, we ask that you notify us immediately of any press plans so that we may opt out on your behalf.

We also ask that you take this opportunity to read our Embargo Policy regarding the discussion, promotion and media coverage of work that is yet to be published by PLOS. As your manuscript is not yet published, it is bound by the conditions of our Embargo Policy. Please be aware that this policy is in place both to ensure that any press coverage of your article is fully substantiated and to provide a direct link between such coverage and the published work. For full details of our Embargo Policy, please visit http://www.plos.org/about/media-inquiries/embargo-policy/.

Thank you again for choosing PLOS Biology for publication and supporting Open Access publishing. We look forward to publishing your study.

Sincerely,

Christian

Christian Schnell, PhD

Senior Editor

PLOS Biology

cschnell@plos.org
